# Recent Progress in the Synthesis of Benzoxazin-4-Ones, Applications in N-Directed Ortho-Functionalizations, and Biological Significance

**DOI:** 10.3390/molecules29235710

**Published:** 2024-12-03

**Authors:** Ziad Moussa, Mani Ramanathan, Harbi Tomah Al-Masri, Saleh A. Ahmed

**Affiliations:** 1 Department of Chemistry, College of Science, United Arab Emirates University, Al Ain P.O. Box 15551, United Arab Emirates; ramanathanmani@uaeu.ac.ae; 2 Department of Chemistry, Faculty of Sciences, Al al-Bayt University, P.O. Box 130040, Mafraq 25113, Jordan; 3 Department of Chemistry, Faculty of Science, Umm Al-Qura University, Makkah 21955, Saudi Arabia; 4 Department of Chemistry, Faculty of Science, Assiut University, Assiut 71516, Egypt

**Keywords:** 4*H*-benzoxazine-4-ones, annulation, fused, regioselective, C-H activation, directing group

## Abstract

The development of efficient synthetic procedures to access fused N, O-heterocyclic skeletons has been a pivotal research topic in organic synthesis for several years. Owing to the applications of N, O-fused heterocycles in organic synthesis, material sciences, and medicinal chemistry, significant efforts have been dedicated to design novel methods for their construction. To this end, 1,3-benzoxazin-4-ones are privileged candidates for N, O-heterocyclic molecules often found in natural products, agrochemicals, and materials science applications. In this review, we aim to summarize the existing literature on the synthesis of 1,3-benzoxazin-4-ones from 2010 onwards. Moreover, 1,3-benzoxazin-4-ones have also been identified as an excellent native directing group for the ortho-functionalization via C-H activation, which is often a strenuous task requiring pre-functionalized substrates. In the latter part of this report, we compiled several interesting examples of *N*-directed functionalizations of 1,3-benzoxazin-4-ones. Additionally, to emphasize biological importance, recent developments on the anticancer evaluations of benzoxazine-4-one core are included. We believe that by harnessing the methodologies discussed herein, new possibilities could be unlocked for the synthesis of fused N, O-heterocycles, leading to the development of novel biologically active compounds and functional materials.

## 1. Introduction

**Figure 1:**
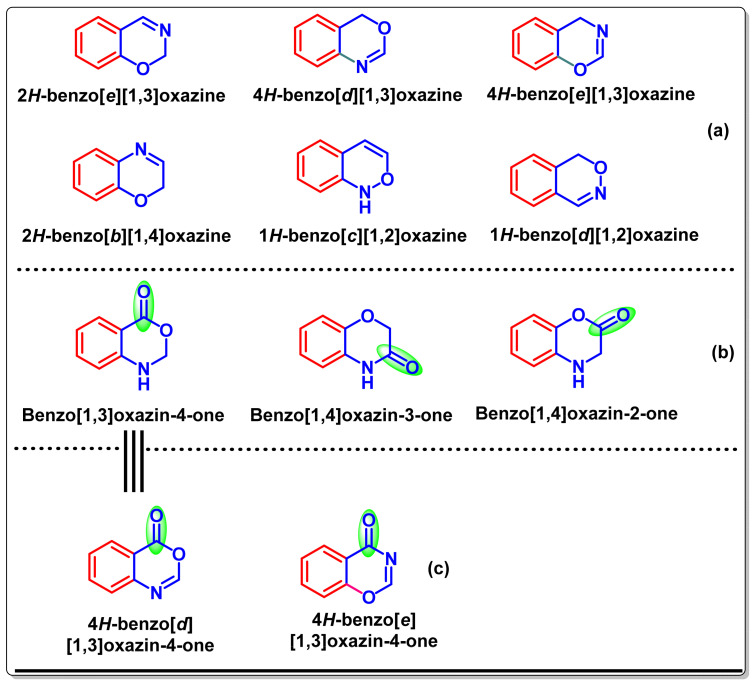
Chemical structures of benzoxazines and benzoxazinone congeners. (**a**). Isomeric structures of benzoxazines; (**b**). Structural isomers of benzoxazinones; (**c**). Two structural congeners of benzo [1,3]oxazin-4-ones.

Benzoxazines are an important class of isomeric bicyclic *N*, *O*-heterocyclic compounds with a general molecular formula, C_8_H_7_NO, containing an aromatic core fused to oxazine ring [1]. Based on the relative position of double bond and *N*- and *O*-atoms in the oxazine core, isomers are named as 1,2-, 1,3- and 1,4-benzoxazines (Figure 1a). Popular drugs such as etifoxine, elbasvir, and apararenone are known to have a benzoxazine structure in their skeleton [2,3]. Additionally, benzoxazine resins (polybenzoxazines) are utilized in fiber-reinforced plastics and adhesives [4,5,6]. On the other hand, based on the position of the carbonyl unit, three important structural congeners are known for benzoxazinones, including benzo [1,3]oxazin-4-ones, benzo [1,4]oxazin-3-ones and benzo [1,4]oxazin-2-ones (Figure 1b). 

**Figure 2:**
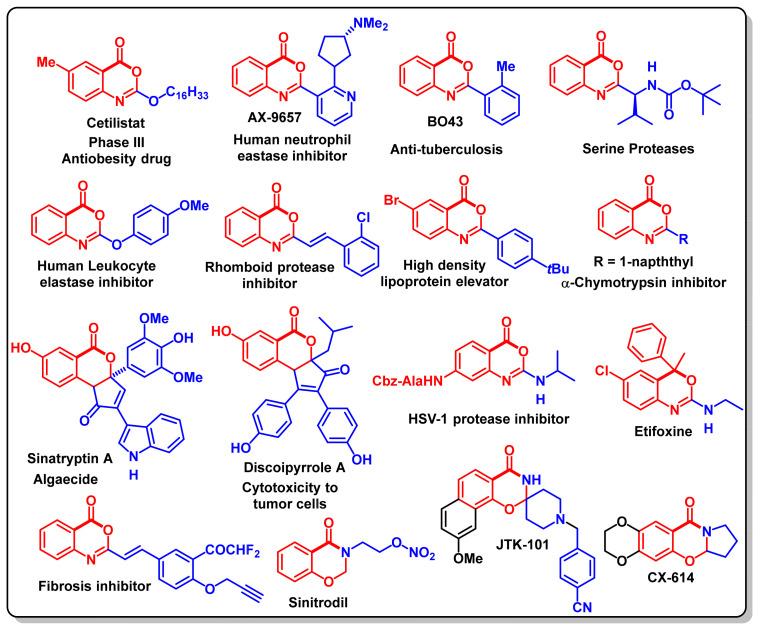
Biologically important benzoxazine-4-one derivatives.

Benzoxazinones are an important class of fused heterocycles with a broad spectrum of biological properties (Figure 2). They exhibit activities including anticancer [7], antibacterial [8], antiviral [9], antifungal [10], antimalarial [11], anti-Alzheimer [12], antiphlogistic [13], antidiabetic [14], antioxidant [15], antiobesity [16], human leucocyte elastase inhibition [17], and serine protease inhibition [18]. These ubiquitous cores are also present in numerous natural and pharmaceutical products, such as Cetilistat [19,20], AX-9657 [21], Sinatryptin A [22,23], Discoipyrrole A [24], Cephalandole A [25,26], Evodiamide A [27], and Flumioxazin [28,29,30,31]. Among these structural analogues, benzo[1,3]oxazin-4-ones are widely known for their extensive syntheses, reactivity, and applications [32]. Structurally, benzo [1,3]oxazin-4-ones are subdivided into two congeners, 4*H*-benzo [1,3][*d*]oxazin-4-ones and 4*H*-benzo [1,3][*e*]oxazin-4-ones (Figure 1c). 

**Figure 3:**
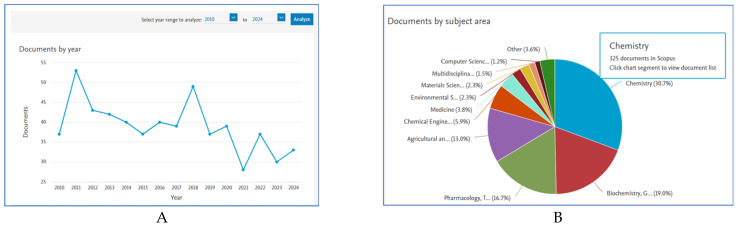
(**A**) Scopus results for the key term search “ benzoxazine 4 one” for the years 2010–2024; (**B**) Document by subject area analyzed by Scopus.

Their core structural framework contains two reactive sites (C2 and C4) with partial positive charges, thus making them more reactive. Vinyl- and alkynyl-substituted benzoxazinone-derived zwitter-ionic species are efficiently employed in the asymmetric preparation of aza-heterocycles via catalytic [4+1], [4+2] and [4+3] dipolar cycloaddition reactions [33]. Importantly, these compounds are employed as a building block for the synthesis of quinazolinones, 4-hydroxy-quinolinones, benzothiazin-4-thione, amidobenzoate, tetrazolyl benzoic acid, and imidazole carboxamide derivatives [34,35]. Synthetically useful multi-substituted indoles and 2,5-disubstituted oxazolines are also prepared from benzoxazine-4-ones under suitable conditions [36,37]. Furthermore, a regiospecific carbonyl oxygen-directed annulation of 2-arylbenzoxazin-4-ones with alkynes resulted in the annulated products [38]. Benzoxazin-4-ones can undergo ring opening by DBU (non-nucleophilic base) and lead to synthetically useful aminopropyl caprolactam [39]. In addition, 1,3-benzoxazin-4-ones are often utilized in the preparation of functional polymers [40], optoelectronic devices [41], and fluorescence emission materials [42]. As well-known plant allelochemicals, benzoxazinones exhibit potential phytotoxic activity in various plants [43] and have served as a raw material for the construction of insecticidal compounds [44]. In recent years, several novel procedures were developed to access 1,3-benzoxazin-4-ones with excellent selectivity and yields. As part of our ongoing research on 1,3-benzoxazin-4-one derivatives, we aim to summarize the synthetic developments and late-stage functionalization (directing group assisted functionalization) from 2010 onwards. This review is organized based on the types of substrates used, such as functionalized anthranilic acids, benzoic acids, amides, isatoic anhydrides, aryl halides, indoles, and isatins, and it describes a schematic representation, some selected examples, a mechanistic illustration, and some post-synthetic applications where applicable. In addition, the synthesis of fused derivatives and 1,3-benzo[*e*]oxazin-4-ones are also discussed. In the later part of the review, we emphasize the applications of 1,3-benzo[*d*]oxazin-4-ones as an efficient *N*-centered directing group for C-H activation based ortho-halogenation, acetoxylation, hydroxylation, benzoxylation, and more. Scopus search results for documents per year data further confirmed that the research on benzoxazine-4-ones is an active area of study in the chemical sciences (Figure 3A). Additionally, these cores are widely involved in chemistry (30.7%), biochemistry (19.0%), pharmacology (16.7%), and the rest in other multi-disciplinary branches (Figure 3B). To the best of our knowledge, there is no comprehensive review available on the synthesis and applications of benzoxazine-4-ones in the past ten years. Considering all this, this account would provide an extensive guidance towards the synthesis and applications of benzoxazine-4-ons. 

## 2. Preparation of Benzo [1,3]-Oxazin-4-One Derivatives from Anthranilic Acids 

The multifaceted reactivity and ease of availability of anthranilic acids enabled their wide utility in organic synthesis. In 1902, Heller and Fiesselmann reported the first synthesis of 2-aryl-4*H*-1,3-benzoxazin-4-ones by treating anthranilic acids with aroyl chlorides in the presence of excess pyridine [45]. Since then, numerous approaches (cascade annulations, oxidative condensations, cross-dehydrogenative approaches, transition-metal catalyzed coupling reactions) have been reported from anthranilic acid derivatives under various conditions.

**Scheme 1:**
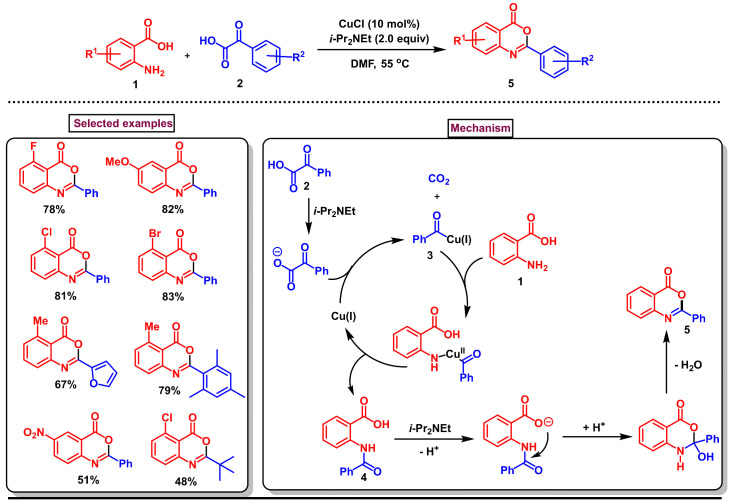
Preparation of benzo[*d*][1,3]oxazin-4-ones via CuCl-catalyzed decarboxylative coupling.

A one-pot CuCl-catalyzed decarboxylative coupling approach was reported to access 2-substituted-4*H*-benzo[*d*][1,3]oxazin-4-ones (**5**) from readily available α-keto acids (**2**) and anthranilic acids (**1**) under mild conditions via an amidation process (Scheme 1) [46]. This reaction proceeded well with various anthranilic acids and α-keto acids (alkyl, aryl, heteroaryl), furnishing the desired products up to 87%. The presence of an electron-withdrawing -NO_2_ group in the anthranilic acid resulted in lower yields (51%). Control experiments proved the formation of amide from anthranilic acid is the key step in this transformation. Mechanistically, the phenylglyoxylic acid anion generated in the presence of DIPEA was reacted with Cu(I)-species and decarboxylated to yield the intermediate (**3**). A further insertion of anthranilic acid generated amide (**4**). Deprotonation, intramolecular cyclization, and dehydration rendered the desired product (**5**). The scalability, utilization of readily available precursors, and economically affordable Cu(I)-catalyst are the merits of this report.

**Scheme 2:**
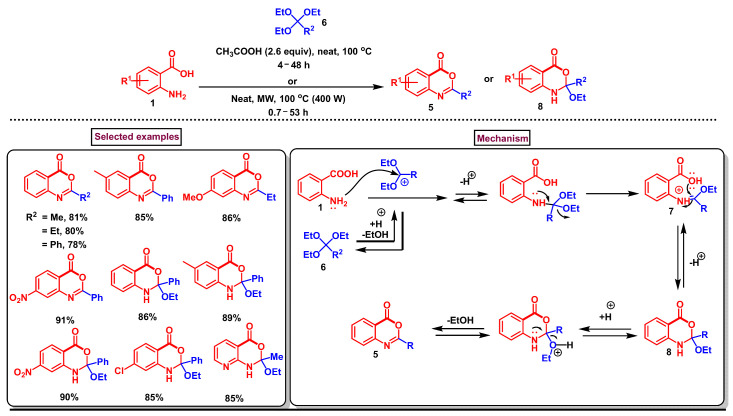
Preparation of benzo[*d*][1,3]oxazin-4-ones via acid-catalyzed cyclization of ortho-esters.

The acid-catalyzed reaction of anthranilic acids **(1)** with ortho esters **(6)** resulted in the formation of benzo[*d*][1,3]-oxazin-4-ones **(5)** and 1,2-dihydro-4*H*-benzoxazine-4-ones **(8),** as reported by Bunce et al. (Scheme 2) [47]. This transformation was studied under thermal and microwave-assisted conditions. In a few cases, where the final elimination of ethanol was difficult, (±)-2-alkyl/aryl-2-ethoxy-1,2-dihydro-4*H*-benzoxazine-4-ones were obtained. In general, the electron-withdrawing groups on the aromatic ring favored the dihydro intermediate, whereas electron-donating substituents led to benzoxazine-4-ones. This observation was mainly due to the diminished availability of lone pair electrons on N1 in the presence of electron-withdrawing substituents on the aryl ring which are crucial for elimination of ethanol. A strong electron-withdrawing pyridine ring in place of an aryl ring resulted in no product. It was observed that the reaction of anthranilic acid and triethyl orthobenzoate under thermal conditions for 48 h afforded the 4*H*-benzoxazine-4-ones **(5),** while shortening the time to 24 h furnished the dihydro intermediate. The inseparable mixture of benzoxazinone and the dihydro product was obtained with triethyl orthobutyrate. The formation of iminium intermediate **(7)** from the reaction of anthranilic acid **(1)** and the stabilized carbocation was observed via proton exchange and the loss of ethanol. Further ring closure and the loss of another molecule of ethanol resulted the product **(5)** through the dihydro intermediate **(8)**.

**Scheme 3:**
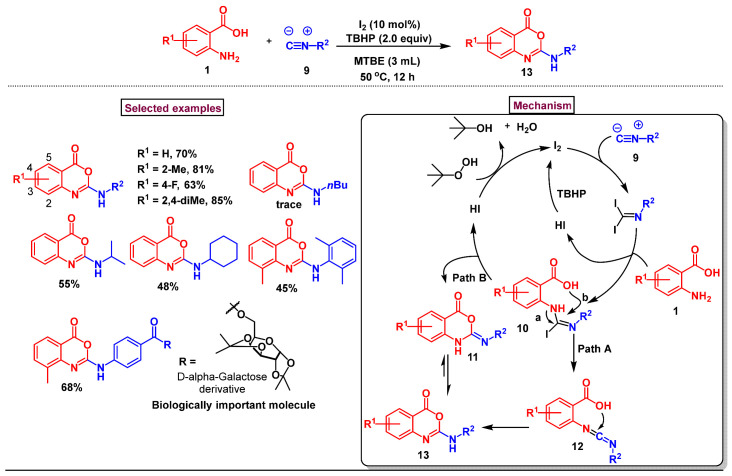
Preparation of benzo[*d*][1,3]oxazin-4-ones via I_2_/TBHP-mediated oxidative coupling.

Isocyanides are excellent C1 building blocks often utilized for the construction of various nitrogen heterocycles [48]. By combining I_2_/TBHP-mediated green oxidative coupling and isocyanide insertion, a transition-metal-free approach was disclosed to access diverse range of 2-aminobenzoxazin-4-ones under mild conditions (Scheme 3) [49]. Other than the primary isocyanides, others, such as secondary, tertiary, and aromatic precursors, led to moderate yields. Moreover, 2-aminobenzoic acids with halogens and electron-donating groups offered the corresponding products in acceptable yields (27–85%). On the other hand, no desired product was obtained with NO_2_ substitution. To illustrate the applicability, a pharmaceutically important 2-aminobenzoxazin-4-one derivative was prepared using galactose-derived aryl isocyanides. Additionally, under slightly modified conditions, a series of 2-aminoquinozolines and 2-aminobenzoxazines were obtained by employing 2-(aminomethyl)anilines and (2-aminophenyl)methanol as binucleophiles. The mechanistic pathway proceeded with the 1,1-addition of iodine to isocyanide **(9),** followed by a reaction with **(1)** via dehydrohalogenation to afford the intermediate **(10)**. The desired product **(13)** could be obtained either by isomerization of **(11)** (path a) or by the intramolecular cyclization of carbodiimide **(12)** (path b).

**Scheme 4:**
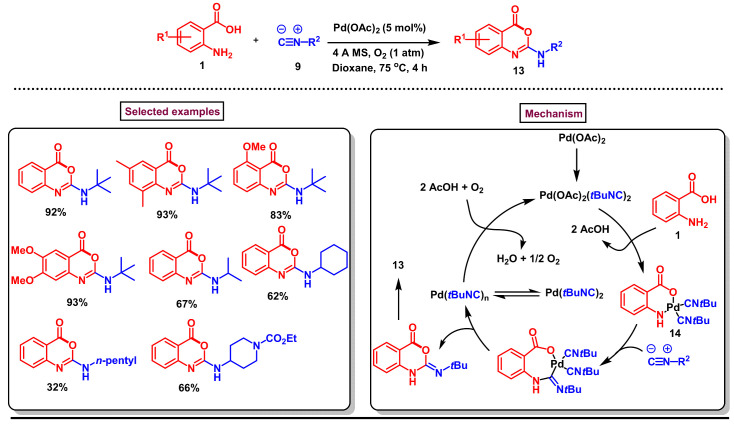
Pd-catalyzed aerobic oxidative coupling of anthranilic acid with isocyanides.

The synthetic potential of isocyanide was further exploited under Pd-catalyzed oxidative conditions to access a series of 2-aminobenzoxazinones **(13)** (Scheme 4) [50]. Undesired nucleophilic pathways and de-carbonylation were avoided under these mild conditions. To expand the scope of various isocyanides (other than *t*BuNC), slightly modified conditions were applied (Pd(OAc)_2_ (10 mol%, Dioxane, 100 °C, 4 h), under which 1° 2°, cyclic isocyanides were studied. Electron-rich substituents (-OMe) on anthranilic acids were effective, whereas electron-withdrawing groups (-F, -CF_3_) required slightly more catalyst loading to achieve good yields. However -NO_2_, -COOH, -I afforded only trace products. Mechanistic studies demonstrated that the initial interaction with active Pd species by anthranilic acid led to Pd complex **(14)**. The further migratory coordination of isocyanide, reductive elimination, and isomerization yielded the product **(13)**. Molecular oxygen re-oxidized Pd(0) for the next catalytic cycle. A gram-scale experiment was also observed to showcase synthetic potential. Overall, an excellent atom economy was observed with water as the only by-product.

**Scheme 5:**
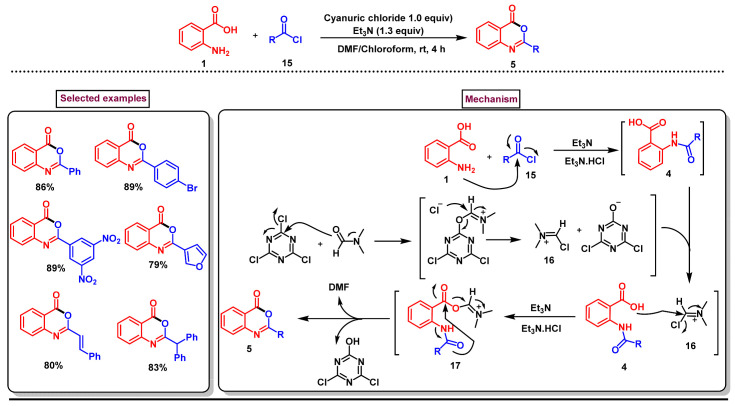
Cyanuric chloride/DMF-mediated synthesis of benzoxazine-4-ones from anthranilic acids.

Cyanuric chloride is frequently used to convert carboxylic acid into an active ester during the preparation of amides, nitriles, and acid chlorides. The cyanuric chloride/DMF mixture is used for the synthesis of ketoximes and the conversion of aliphatic alcohols into halides. An iminium cation **(16),** generated from cyanuric chloride/DMF, efficiently cyclized the *N*-acylated anthranilic acid **(4)** into 2-functionalized benzoxazine-4-ones **(5)** via cyclodehydration (Scheme 5) [51]. A series of 2-aryl, heteroaryl, styryl, and amino functionalized benzoxazine-4-ones were obtained under mild conditions. In comparison to the traditional cyclization strategies which often require heating, this process occurred at ambient temperature and furnished the desired products in higher yields (up to 89%). Mechanistically, this method proceeded through the formation of iminium **(16)** and active ester intermediates **(17)**.

**Scheme 6:**
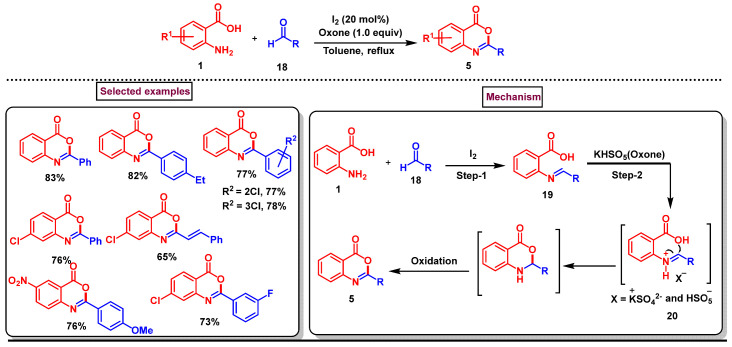
I_2_-catalyzed condensation/cyclization strategy to prepare 2-arylbenzoxazin-4-ones.

An appealing, transition-metal-free oxidative cascade sequence to access 2-arylbenzoxazin-4-ones was reported using anthranilic acid **(5)** and aldehydes **(18)** under I_2_-catalyzed conditions by Iyer et al. (Scheme 6) [52]. Economically affordable oxone was used as the sole oxidant in this process. Structurally diverse anthranilic acids **(1)** (3-Cl, 4-NO_2_) and aryl aldehydes **(18)** (4-ethyl, 4-OMe, 4-Br, 4-Cl, 4-NO_2_) were tested to afford the desired products **(5)** under the developed conditions. The presence of electron-withdrawing -NO_2_ group on both the substrates lowered the yield (69%). Alkyl aldehydes were found to be unreactive under these conditions. The reaction pathway was initiated with the I_2_-catalyzed formation of imine **(19),** followed by the cyclization of iminium salt **(20)** and oxidation. Easy synthetic manipulations with readily available precursors are the notable advantages of this report. 

**Scheme 7:**
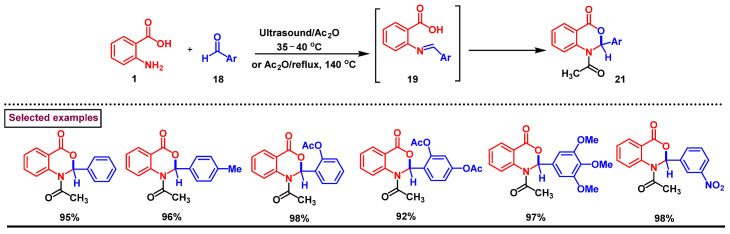
Ultrasound-irradiated synthesis of 1,2-dihydro-4*H*-1,3-benzoxazin-4-ones.

A similar condensation procedure under ultrasound conditions was developed using anthranilic acids **(1)** and aryl aldehydes **(18)** in the presence of acetic anhydride to access *N*-acetyl-2-aryl-1,2-dihydro-4*H*-1,3-benzoxazin-4-ones **(21)** via the imine intermediate **(19)** [53]. This one-pot sonochemistry offered the products in excellent yields under the transition-metal-free conditions. (Scheme 7). Optimizational studies revealed that lower yields were obtained in the presence of solvents (45–65%) under thermal conditions. However, excess acetic anhydride (>50 mmol) under ultrasonic irradiation led to excellent yields (up to 98%) in a relatively shorter time. An excellent substrate scope was observed, and both electron-donating (-Me, -OMe) and electron-withdrawing substituents (-Br, -Cl, -NO_2_) led to the desired products in higher yields (up to 98%). 

**Scheme 8:**
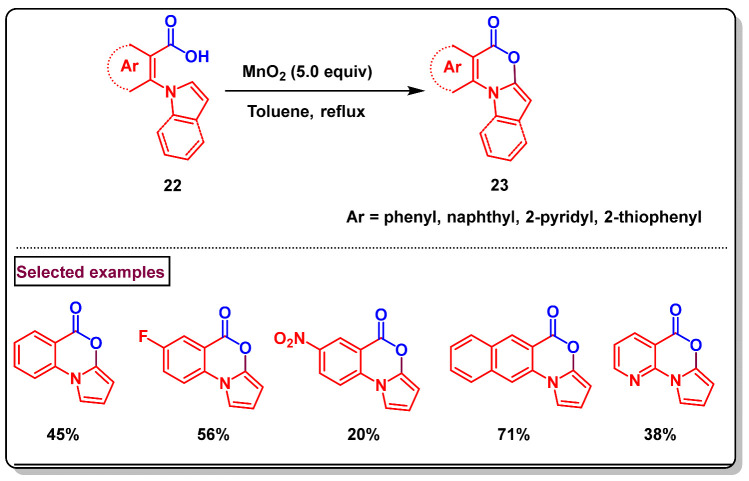
Activated MnO_2_-mediated oxidative C-O coupling.

The temperature-adjustable oxidative ability and the weak Lewis acid character of the activated MnO_2_ were exploited to prepare a series of 5*H*-pyrrolo [1,2-*a*][1,3] benzoxazinones **(23)** from the corresponding ortho-functionalized anthranilic acids **(22)** via an intramolecular C-O cyclization (Scheme 8) [54]. The scope and generalizability of this method were studied with differently substituted precursors, and the yields in most of the cases were poor to moderate (20–56%). Benzoxazine-4-ones fused with naphthalene, and heteroaromatic and sensitive halogen substitutions were accessed under these conditions. The analogous indole derivatives were also accessed under these conditions. Though excess oxidant and longer reaction times are required, considering the sensitivity of pyrrole towards the oxidative conditions, this method is a complementary approach. Extensive efforts to reduce the reaction times incurred by establishing microwave conditions, conducting sealed tube experiments, and the addition of radical scavengers (4-*tert*-butylcatechol) led to inferior results.

**Scheme 9:**
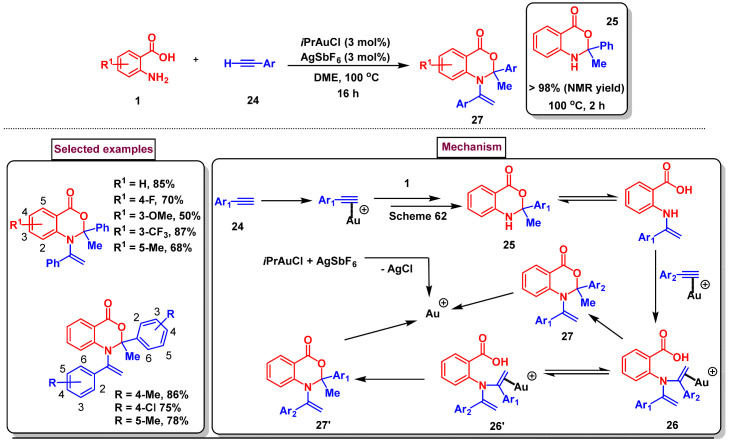
Preparation of benzo[*d*][1,3]oxazin-4-ones via gold-catalyzed heteroannulation.

Non-polarized simple alkynes **(24)** were hetero-annulated with anthranilic acids **(1)** under gold-catalyzed conditions to afford 1,2-dihydro-4*H*-benzo[*d*][1,3]oxazine-4-ones **(27)** via the double incorporation of alkynes (Scheme 9) [55]. As described the interaction of the gold–alkyne complex with (**1**) gave the cyclized product (**25**). Another set of interactions of (**25**) with the alkyne–gold complex and intramolecular cyclization yielded the product (**27** or **27’**) via the bisenamine–gold complex (**26** or **26’**). In addition, authors detected (**25**) in the NMR analysis in an experiment with shortened reaction time. Regardless of the order, the sequential addition of two different terminal alkynes resulted in the formation of a regioisomeric mixture of products, indicating (**25**) did not directly react with a second alkyne. Anthranilic acids **(1)** with electronically distinct groups (-OMe, Halogens, -NO_2_, -CF_3_) delivered the products in synthetically useful yields (up to 87%). Substitution adjacent to *N*-center severely hampered the reaction; however, with excess alkyne (10 equiv.) under neat conditions, the anticipated product was obtained in a good yield (83%). A gram-scale experiment was observed to highlight the synthetic utility. High functional group tolerance and atom economy were achieved under this gold-catalyzed heteroannulation.

**Scheme 10:**
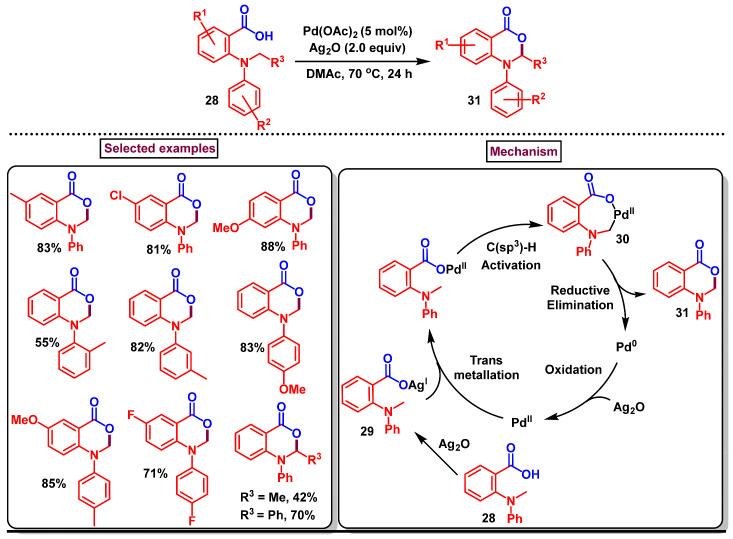
Intramolecular C-H activation strategy to access 1,2-dihydro-(4*H*)-3,1-benzoxazin-4-ones.

In the intramolecular C-H activation of *N*, *N*-difunctionalized anthranilic acids are very rarely investigated, owing to the presence of multiple C-H bonds. By exploiting the structural features of *N*-alkyl-*N*-arylanthranilic acid **(28),** which carry C(sp^2^)-H and C(sp^3^)-H bonds in the same molecule, Guo et al. developed a Pd-catalyzed intramolecular controllable C-H activation strategy using Ag_2_O as an oxidant to access 1,2-dihydro-(4*H*)-3,1-benzoxazin-4-ones **(31)** (Scheme 10) [56]. Mechanistically, Ag_2_O, as an oxidant, promoted the activation of the C(sp^3^)-H bond in the alkyl group by forming the silvercarboxylate complex **(29)** and the cyclization of the C-O bond to provide the desired 1,2-dihydro-4*H*-benzoxazine-4-ones from the Pd-complex **(30)** via reductive elimination. By switching to the Cu(OAc)_2_ oxidant, carbazoles were obtained via the C(sp^2^)-H activation/C-C cyclization strategy. The electronic effect of the substituents were less influential as both the electron-donating (-Me, 83%) and the electron-withdrawing groups (-Cl, 81%) led to higher yields. Steric effect was found to be crucial as ortho-substitution significantly lowered the product yield (55%). Considering all of this, this challenging oxidant-controlled intramolecular C-H activation strategy displayed a reasonable substrate scope and delivered two different N-heterocycles with high selectivity.

**Scheme 11:**
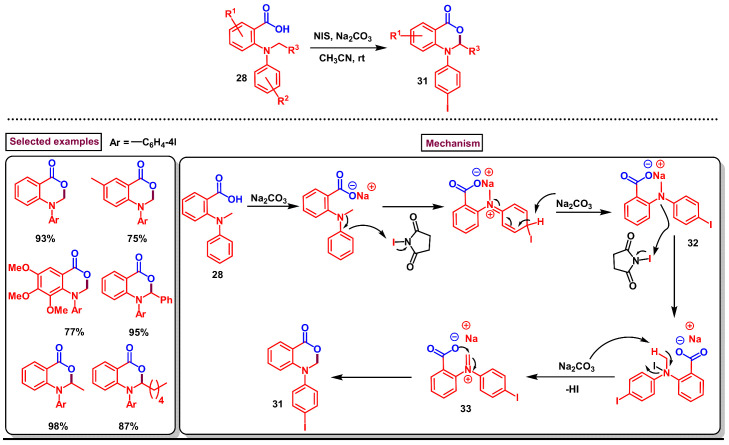
Preparation of 1,2-dihydro-benzoxazin-4-ones via NIS-mediated α-functionalization.

An alternative cross-dehydrogenative coupling strategy for the selective functionalization of C-H next to a tertiary amine followed by a direct C-O bond formation under transition-free conditions was developed by Du et al. (Scheme 11) [57]. In this process, *N*-iodosuccinimide effectively mediated the α-functionalization of the sp^3^ C-H bond of *N*-alkyl-*N*-aryl anthranilic acids **(28)** and led to 1,2-dihydro-benzoxazin-4-ones **(31)** via the oxidative C-O bond formation. The dual role of NIS as an iodinating agent and an oxidant was demonstrated under this transition metal-free procedure. Though a mixture of iodinated and non-iodinated products were obtained initially, the addition of Na_2_CO_3_ enhanced the formation of iodinated product chemo-selectively, leading to an excellent yield. In the case of the long-chain *N*-alkyl groups, decomposition was observed. Stronger electron-donating groups favored the formation of di-iodinated products. However, the N-aryl groups substituted with electron-withdrawing (halogens, -CF_3_, -NO_2_) groups led to only non-iodinated annulated products in good yields (up to 91%). It was observed that the N-Ac or N-Ts functionalities proved unsuccessful. Mechanistic insights illustrated that the deprotonation of carboxylic acid **(28)** and the electrophilic iodination yielded the intermediate **(32)**. Further interaction with NIS/Na_2_CO_3_ provided the imine **(33)**, which was converted into product **(31)** via intramolecular annulation. In a parallel study, the authors replaced the carboxylic acid in the *N*-OMe amide functionality and obtained 1,4-benzodiazepine in excellent yields. However, only para-iodinated starting material was found when the nucleophilic alcohol group was embedded.

**Scheme 12:**
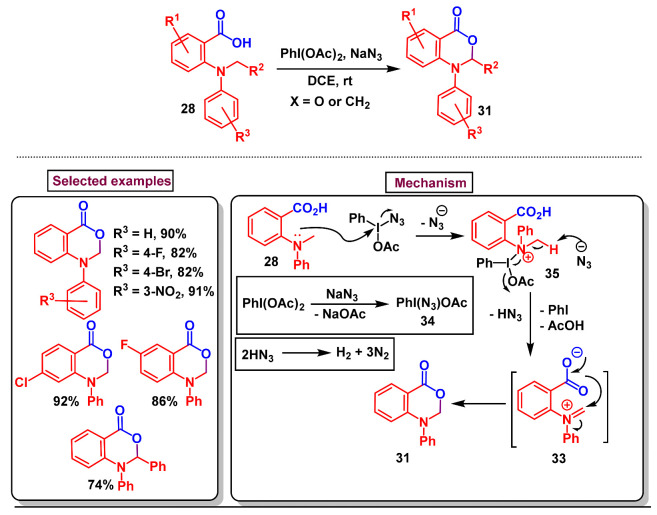
Preparation of 1,2-dihydro-benzoxazin-4-ones via PIDA-mediated oxidative approach.

Another example of a hypervalent iodine (III)-mediated, cross-dehydrogenative coupling for the direct C-O bond formation was reported by Zhao and Du’s group (Scheme 12) [58]. In this case, the intramolecular α- C-H activation/C-O bond formation of the tertiary amine substrate was featured in the presence of a PhI(OAc)_2_/NaN_3_ oxidative system to access 1,2-dihydro-(4*H*)-3,1-benzoxazin-4-ones **(31)**. It should be noted that tertiary amines with two aryl units are mandatory for this transformation. On the other hand, a trace amount of product (15%) was formed when two benzyl groups were installed on the nitrogen center. It was observed that strong electron-withdrawing (-OMe) substituents lowered the product yield (42%), whereas moderately electron-donating (-Me) halogens (-F, -Br, -Cl) and electron-withdrawing groups (-NO_2_, -CF_3_) were afforded the desired products in excellent yields (up to 92%). The authors explained the presence of two aryl units attached to the N-center was necessary, as the benzyl or acyl groups led to inferior results. Radical trapping experiments using TEMPO ruled out the radical mechanism. The nucleophilic attack of **(28)** in situ generated unstable azidoiodinane PhI(N_3_)OAc **(34)** and resulted in the ammonium ion **(35)**. Deprotonation by the basic N_3_ ion, the elimination of iodobenzene, and acetic acid led to the imine **(33)**. Further intramolecular cyclization formed product **(31)**. Unlike the author’s previous report [57], benzoxazines were obtained in reasonable yields when carboxylic acid was replaced with nucleophilic alcohol (up to 70%).

## 3. Preparation of Benzo[*d*][1,3]-Oxazin-4-Ones from Functionalized Amides

**Scheme 13:**
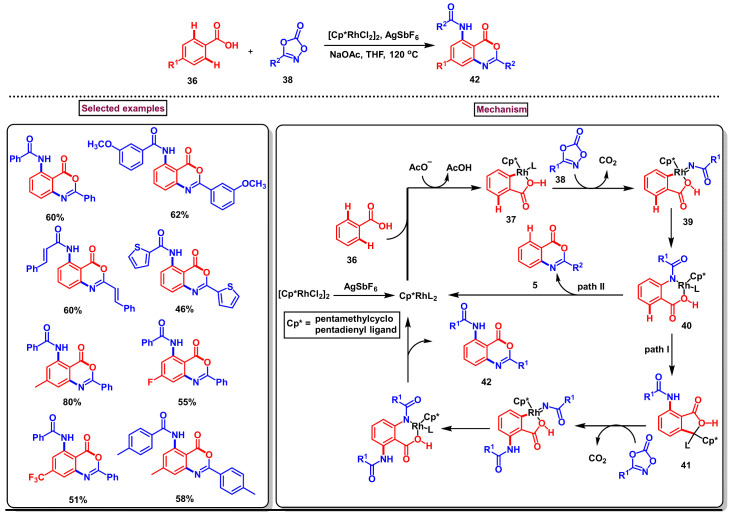
Rh(III)-catalyzed cascade dehydrative annulation to access 2,5-disubstituted benzoxazine-4-ones.

Dioxazolones, important building blocks with an excellent coordination ability often used in directing groups (nitrone, amide, ketones, imines), enabled ortho C-H amination strategies. By utilizing the carboxylic acid (**36**) as a directing group, a Rh(III)-catalyzed cascade sequence with dioxazolones (**38**) was reported to prepare 2,5-disubstituted benzoxazine-4-ones (**42**) (Scheme 13) [59]. In this method, carboxylic acid served as a directing group and led to double amidation, subsequently enabling the dehydrative annulation to yield the desired products. The formation of double-amidated product prior to dehydrative cyclization was proven by control experiments, thus confirming the directing role of carboxylic acid in the second C-H activation. The study of substituent effects at the para- and metapositions of the aryl ring on (**38**) showed that electron-donating groups (-Et, -*t*Bu, -OMe) furnished the products in higher yields (63–72%) than the electron-withdrawing groups (-CF_3_, 47%). Though moderate yields were obtained in the case of the thiophene and styryl derivatives, alkylated dioxazolones failed to be involved in this transformation. Similar electronic effects were observed in the case of benzoic acids (36). It should be noted that ortho and para-functionalized benzoic acids were not amenable under these conditions. The kinetic isotopic effect value (KIE = 8.1) undoubtedly confirmed that C-H activation was the rate-determining step. Rhodacycle (**37**) was generated from the interaction of the active Rh species and (**36**), which was coordinated with dioxazolone (**38**) and generated (**39**) via CO_2_ extrusion. Further migratory insertion and the second C-H activation provided the rhodacycle (**41**) (path I). The sequential reaction with (**38**), migratory insertion, and dehydrative cyclization led to (**42**). When the dimethylamino group was employed on oxazolone (**38**), 2-substituted benzoxazine-4-one was obtained from intermediate (**40**) (path II).

**Scheme 14:**
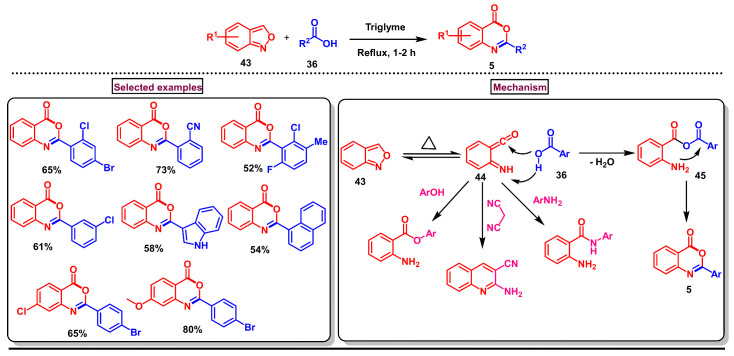
Preparation of 2-arylated benzoxazinones from anthranils and carboxylic acids.

The labile nature of the N-O bond in anthranil is often exploited under transition-metal-catalyzed conditions to prepare a wide range of *N*-heterocycles via the 2-formyl aryl nitrene intermediate. On the other hand, reactive ketene **(44)** was generated under the high-temperature stimulation of the N-O bond of anthranil **(43),** which was further trapped by carboxylic acid **(36)** to produce a series of 2-arylated benzoxazinones **(5)** (Scheme 14) [60]. Optimizational studies revealed that fewer polar solvents (triglyme) favored the product formation. Aliphatic carboxylic acids were found unreactive under these conditions; however, variously substituted aryl, heteroaryl, alkenyl carboxylic acids **(36)** were smoothly converted into the corresponding 2-functionalized benzoxazin4-ones **(5)** in moderate to good yields (50–80%). In situ-generated ketene **(44)** reacted with acid **(36)** and led to anhydride **(45)**. Further intramolecular condensation generated the product **(5)**. The low-cost reagents and shorter reaction times are the advantages of this method. The authors have also subjected various other nucleophiles, such as amines, malononitriles, and phenols, to anthranils and obtained synthetically useful amides, quinolines, and esters.

**Scheme 15:**
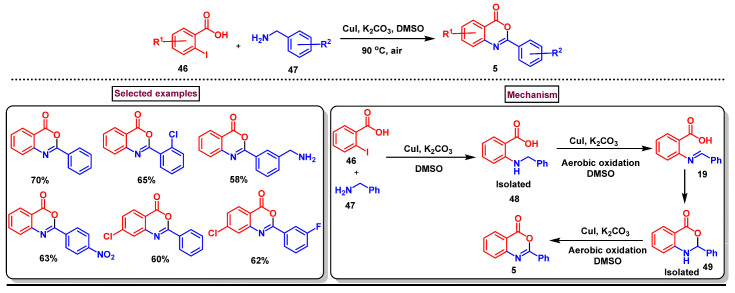
Cu-catalyzed synthesis of benzoxazine-4-ones via aerobic oxidative coupling.

A domino, Cu-catalyzed reaction of arylmethanamines **(120)** with 2-iodobenzoic acids **(119)** to prepare functionalized benzoxazine-4-ones **(5)** under tandem and ligand-free aerobic conditions was disclosed by Sathiyanarayanan et al. (Scheme 15) [61]. Various copper catalysts, bases, and reaction temperatures were screened to find the best reaction conditions. This Cu-catalyzed domino process tolerated a wide range of benzyl amines, bearing -Br, -Cl, -NO_2_, and NH_2_ and offering the desired products in moderate yields (55–73%). Alkyl methanmines were not reacted. A fair scope was also exerted by the 2-iodobenzoic acids and resulted in moderate yields. The authors observed the exclusive formation of 2-(benzylamino) benzoic acid **(121)** (Ullmann type *N*-arylation) under the influence of the N_2_ atmosphere, which was cyclized under standard conditions to give the desired product in good yields. Mechanistically, the Cu-catalyzed Ullmaan coupling was followed by aerobic oxidation to generate the imine **(19)**. Further intramolecular cyclization and aerobic oxidation yielded the benzoxazine-4-ones **(5)**.

**Scheme 16:**
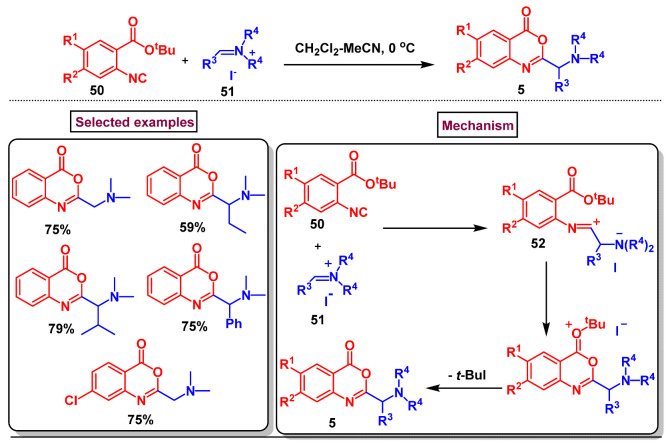
Preparation of benzoxazine-4-ones via the Ugi-type reaction of 2-isocyanobenzoates.

Kobayashi and coworkers (2014) developed a novel, catalyst-free method to prepare 4*H*-3,1-benzoxazin-4-ones **(5)** from the Ugi-type reaction of 2-isocyanobenzoates **(50)** with *N*, *N*-dialkyliminium salts **(51)** (Eschenmoser’s salt) [62]. In situ-generated *N*, *N*-dialkyliminium iodides from the corresponding aldehydes, and amine hydrochlorides were utilized in several cases (Scheme 16). Moreover, 2-isocyanobenzoates **(50)** were accessed from readily available 2-nitrobenzoic acids via a four-step literature process. Dialkyl- and aryl–alkyl-substituted Eschenmoser’s salts **(51)** reacted smoothly to afford the title compounds in moderate yields (up to 79%). An example of a *N*, *N*-dialkyliminium salt with a terminal alkene was also shown to afford the corresponding benzoxazine-4-ones in good yield (75%). The nucleophilic attack of isocyanide **(50)** on the imine carbon **(51)** led to imidoyl cation **(52)**. The further intramolecular cyclization and elimination of the *t*-Bu unit offered the product **(5)**. The reaction proceeded smoothly and tolerated common functional groups. Moderate yields were obtained under these mild conditions.

**Scheme 17:**
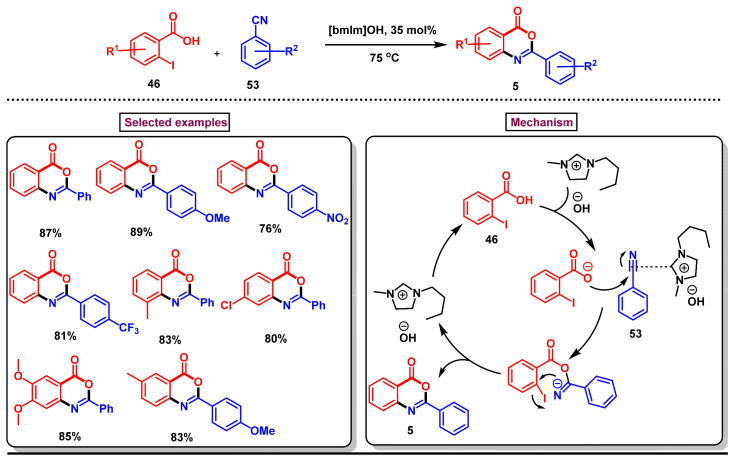
Basic [bmIm]OH-catalyzed synthesis of benzoxazine-4-ones.

Ionic liquids are renowned for their solvating nature and catalytic ability, and they play a vital role in eco-friendly organic transformations. Among these, basic ionic liquids are easy to recover and reuse and possess excellent catalytic efficiency. From this perspective, an eco-friendly benzoxazine-4-ones **(5)** was realized via the basic [bmIm]OH-catalyzed reaction of arylnitrile **(53)** and *o*-iodobenzoic acid **(46)** under mild conditions. (Scheme 17) [63]. Interestingly, this transformation proceeded under solvent-free conditions and the [bmIm]OH was recycled up to five times without any appreciable loss in the yield. The robustness of this method was explored with a wide range of substrates and the corresponding benzoxazine-4-ones were obtained in excellent yields (up to 91%). It was observed that the electron-donating groups (-Me, -OMe) increased the product formation compared to the electron-withdrawing groups (-CF_3_, -NO_2_). The reaction initiated with the nucleophilic attack of the carboxylate anion on nitrile unit **(53),** followed by an intramolecular nucleophilic displacement of the iodo-group, led to product **(5)**. The deprotonation of carboxylic acid and the activation of nitrile were influenced by [bmIm]OH. The authors observed that the reaction could also be effected by organic bases such as DBU, triethyl amine, and pyridine albeit in low yields.

**Scheme 18:**
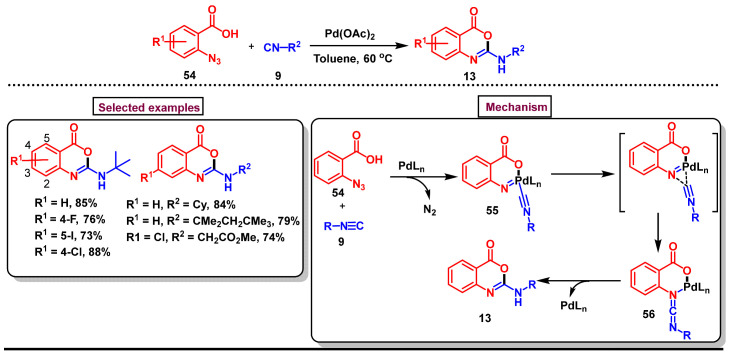
Pd-catalyzed tandem azide-isocyanide coupling of aryl azides with isocyanides.

The Pd-catalyzed reaction of 2-functionalized aryl azides **(54)** with isocyanides **(9)** afforded a series of 2-alkylaminobenzoxazinones **(13)** via the sequential, tandem azide–isocyanide coupling and the intramolecular 6-*exo-dig* cyclization (Scheme 18) [64,65]. Compared to the traditional oxidative coupling of amines with isocyanides, this tandem process exhibited a wide substrate scope. Aliphatic isocyanides (1^o^, 2^o^, 3^o^), including cyclic analogues, were found to be equally reactive in this process, yielding the desired products in good yields (up to 88%). The aryl isocyanides failed to react under these conditions. The generality of the unsubstituted and halogenated 2-azidoebenzoic acids (-Cl, -Br, -I) was tested and appreciable yields were obtained. Based on the control experiments and DFT computational studies, a plausible mechanism was proposed. Accordingly, coordination between Pd(OAc)_2_ and **(54),** followed by the interaction of isocyanide, yielded the key intermediate **(55)**. The further nitrine transfer and intramolecular 6-*exo-dig* cyclization of the carboxylate unit on carbodiimide **(56)** resulted in the desired product **(13)**. The applicability of this method was further extended for the preparation of aminoquinaolinones, benzoxazoles, benzimidazoles, and benzothiazoles.

**Scheme 19:**
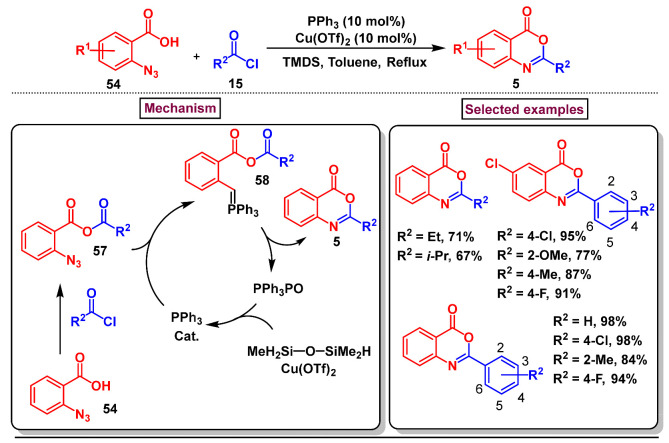
PPh_3_/Cu-catalyzed one-pot aza-Wittig reaction of carboxylic acid to prepare benzoxazine-4-ones.

The inter- and intramolecular aza-Wittig reaction of amides, aldehydes, ketones, and esters is a widely accepted tool for the synthesis of various heterocycles [66,67]. Ding and Huang’s group explored a PPh_3_/Cu-catalyzed one-pot aza-Wittig reaction of carboxylic acid **(54)** to access 4*H*-benzo[*d*][1,3]oxazin-4-ones **(5)** via acid anhydride intermediate **(57)** [68]. Readily available 2-azido benzoic acids and alkyl/aryl acyl chlorides were used in the presence of 10% PPh_3_ catalyst (Scheme 19). Irrespective of the substitution pattern and positions, a diverse range of aryl acyl chlorides, bearing -Me, -OMe, -Br, -Cl, -F, afforded the desired products in excellent yields (up to 98%). Interestingly, alkyl acyl chlorides were also amenable, and the products were obtained in comparatively lower yields (67–73%). The authors have also demonstrated the intramolecular cyclization of 2-azidoaryl anhydrides under similar conditions. Functional groups such as aldehyde, ketone, alkene, cyano, nitro and halogens survived, and the desired 2-arylated benzoxazine-4-ones were obtained in excellent yields (up to 91%). Additionally, the Hammett plot revealed this process was catalyzed at a faster rate using electron-rich phosphines. Mechanistically, this transformation proceeded via iminophosphorane **(58),** and the triphenylphosphine was regenerated through the reduction in triphenylphosphine oxide by the TMDS/Cu(OTf)_2_ system. On the other hand, the authors have also demonstrated the one-pot synthesis of 4-benzylidene-2-aryloxazol-5(4*H*)-ones from 2-azido-3-arylacrylic benzoic acids under identical reaction conditions.

**Scheme 20:**
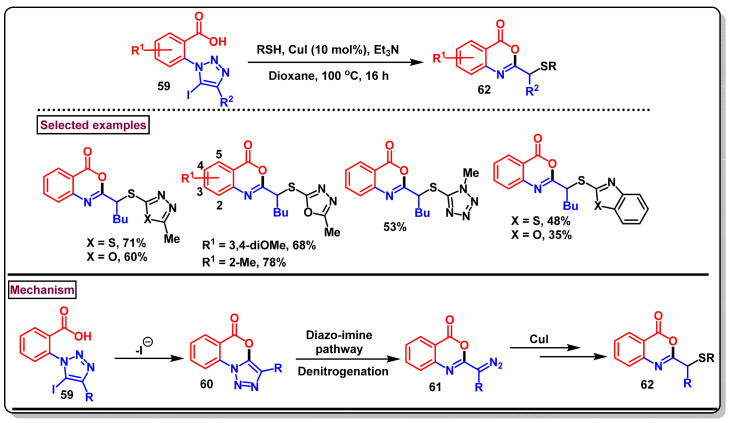
Cu-catalyzed one-pot synthesis of thiolated benzoxazinones.

Latyshev et al. described the nucelophilic intramolecular substitution of 2-(5-iodotriazolyl) benzoic acids **(59)** to access a range of 1,2,3-triazolobenzoxazinones **(60),** which acted as a hidden diazo source (Scheme 20) [69,70,71]. Under Cu-catalyzed conditions, thiolated benzoxazinones **(62)** were obtained via a one-pot cyclization and diazo-capture strategy. The base-induced cyclization of **(59)** in the presence of triethylamine led to triazolobenzoxazinones **(60)** in moderate to good yields (40–74%). Optimizational studies revealed that the usage of stronger bases and polar solvents resulted in the decomposition of **(62)**. The authors described that the diminsihed yields in most of the cases were due to the hydrolytic decomposition of the products during silica gel column chromatography. Alkyl substitutions on the triazole worked better, whereas the aryl unit requried a longer reaction time at 80 °C. The authors further explained that diazo-imine tautomer **(61)** could be captured by thiols under Cu-catalyzed denitrogenative reactions to afford 2-(thiomethyl)benzoxazinones in moderate to good yields. Interestingly, a one-pot Cu-catalyzed cascade process from **(59)** was developed to access **(62)**. Various heterocyclic-tethered thiols were incorporated, and the best yields were obtained from thiadiazole and oxadiazole derivatives (71–68%). It should be noted that the Cu-catalyzed capturing of diazoimine with amines led to anthranilic acid-based amides.

**Scheme 21:**
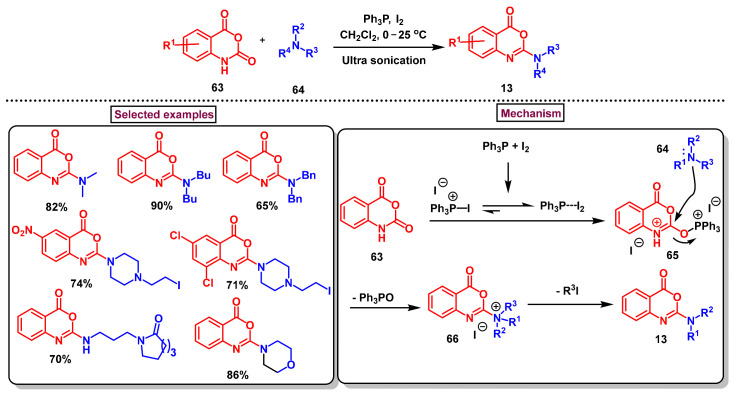
Ph_3_P-I_2_-promoted deoxygenative amination of functionalized isatoic anhydride.

Isatoic anhydride is a potential building block with multifaceted reactivity, often used in the preparation of various heterocycles owing to the electrophilicity of the reactive ester carbonyl and amide unit [72]. The Ph_3_P-I_2_-promoted deoxygenative amination of functionalized isatoic anhydride **(63)** with tertiary amines **(64)** was disclosed to access 2-aminobenzoxazin-4-ones **(13)** under sonochemical conditions (Scheme 21) [73]. Tertiary amines served as both nucleophile as well as base, and they regioselectively reacted with isatoic anhydrides rather than the carbonyl group at C4. The reactivity of various symmetrical and unsymmetrical tertiary amines were studied in detail. Acyclic, symmetrical tertiary alkyl amines (-Me, -Et, -*n*Bu) were smoothly reacted to give the corresponding 2-aminobenzoxazin-4-ones in high yields (up to 90%). In the case of unsymmetrical *N*, *N*-diethylbenzylamine, regioselective *N*-debenzylation occurred to give the desired product in excellent yields (87%). The loss of the isopropyl group was observed when Hünig’s base was employed as an aminating agent. These observations indicate the relative stablity of carbocation during the elimination process is crucial. Due to the competitive nucleophilic attack of pyridine nitrogen, DMAP led to a complex mixture. Interestingly, sterically hindered bicyclic amidines were also involved in this transformation, leading to lactam derivatives after ring opening, followed by hydrolysis. The proposed reaction pathway illustrated that the formation of oxyphosphonium intermediate **(65)** was observed via the phosphorylation of the amide carbonyl unit, which was converted into ammonium salt **(66)** upon reaction with tertiary amine **(64)**. The subsequent *N*-dealkylation liberated product **(13)** and HI. It is worth mentioning that when a secondary amine was employed, an additional nucleophilic attack at the ester carbonyl yielded the ring-opening product. The synthetic utility of this method was demonstrated by a gram-scale synthesis with excellent isolated yield (89%).

**Scheme 22:**
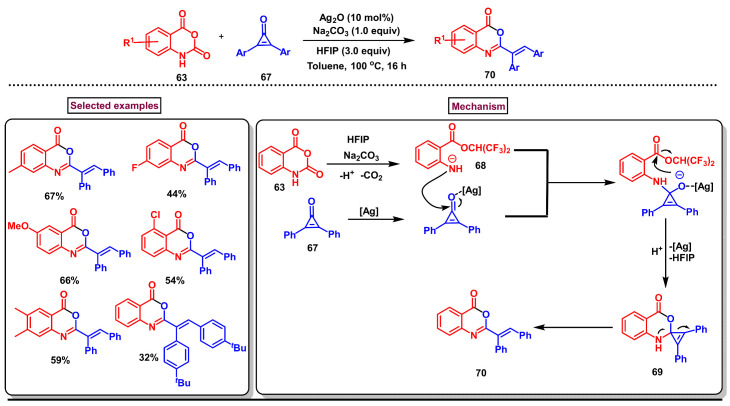
Preparation of 2-alkenyl benzoxazine-4-ones via Ag-catalyzed intermolecular [4+2]-cycloaddition.

The synthetic methods leading to 2-alkyl or 2-aryl functionalized benzoxazine-4-ones have become widely available in recent years. However, despite their biological importance, C2-alkenyl analogues are less known. An elegant approach through an Ag-catalyzed intermolecular [4+2]-cycloaddition strategy to access 2-alkenyl benzoxazine-4-ones **(70)** from readily available isatoic anhydride **(63)** and cyclopropenones **(67)** in the presence of HFIP was described by Wang and Cheng et al. (Scheme 22) [74]. Isatoic anhydrides **(63),** bearing electron-donating groups, displayed higher reactivity than the electron-withdrawing substituents during the cycloaddition with diphenylcyclopropenone **(67)**. Heteroaryl-fused isatoic anhydrides (pyridyl, thiophenyl) were not involved in this transformation. On the other hand, cyclopropenones bearing electron-withdrawing substituents (-F, -CF_3_) on the aryl rings reacted better than the electron-donating substituents (-Me, -*t*Bu). The decarboxylative esterification of isatoic anhydride in the presence of the HFIP-generated nitrogen anion intermediate **(68),** which through nucleophilic attack underwent [4+2] cycloaddition with cyclopropenones **(67)** to yield 2-diarylalkenyl-4*H*-3,1-benzoxazin-4-ones **(70)** via intermediate **(69)**. A fair substrate scope was exerted for both isatoic anhydride and cyclopropenone. 

**Scheme 23:**
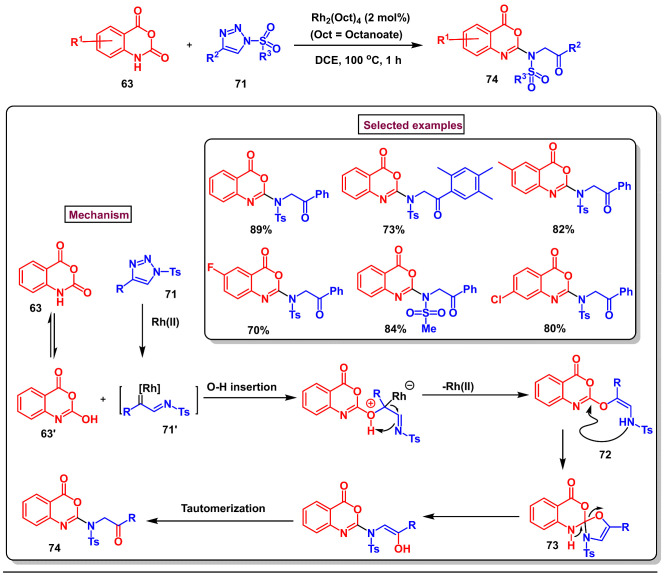
Preparation of 2-aminobenzoxazin-4-ones via O-H insertion of α-imino Rh-carbenoids.

The in situ generation of α-imino Rh-carbenoids from the reaction of catalytic Rh^II^ and *N*-sulfonyl-1,2,3-triazoles followed by 1,1-C-H/O-H insertions is known in the literature. Isatoic anhydride **(63)** was found to react with α-imino Rh-carbenoids **(71′)** via the O-H insertion, and the subsequent rearrangement provided 2-aminobenzoxazin-4-ones **(74)** (Scheme 23) [75]. A range of aryl, heteroaryl, and alkyl bearing triazoles were tested under the optimized conditions (73–89%). It was found that the electronic properties of the substituents had no influence on the outcome of the reaction, whereas the sterically demanding ortho-substitutents slightly lowered the yields. It should be noted that variously substituted sulfonyl moities (*p*-Br, *p*-OMe, *p*-Ph, *p*-*t*Bu), including the mesylate group, displayed excellent compatiblity during this transformation. The O-H insertion of **(63′)** with α-imino Rh-carbenoid species (**71′**) followed by proton abstraction yielded the enamine **(72)**. The formation of oxazole **(73)** via intramolecular cyclization was followed by ring opening and tautomerization, furnishing the product **(74)**. The scope of this denitrogenative approach was further extended to prepare fused benzoxazin-4-ones and 5-amino-oxadiazoles from oxadiazolones and triazoles under similar reaction conditions. In addition, the authors demonstrated a gram-scale synthesis and a one-pot apporach by combining the in situ preparation of triazole (via Cu^I^-mediated 1,3-dipolar cyclization), followed by a Rh^II^-catalyzed reaction with isatoic anhydride to offer the desired product in comparable yields (up to 75%)

## 4. Preparation of Benzo[*d*][1,3]-Oxazin-4-Ones from Isatoic 

**Scheme 24:**
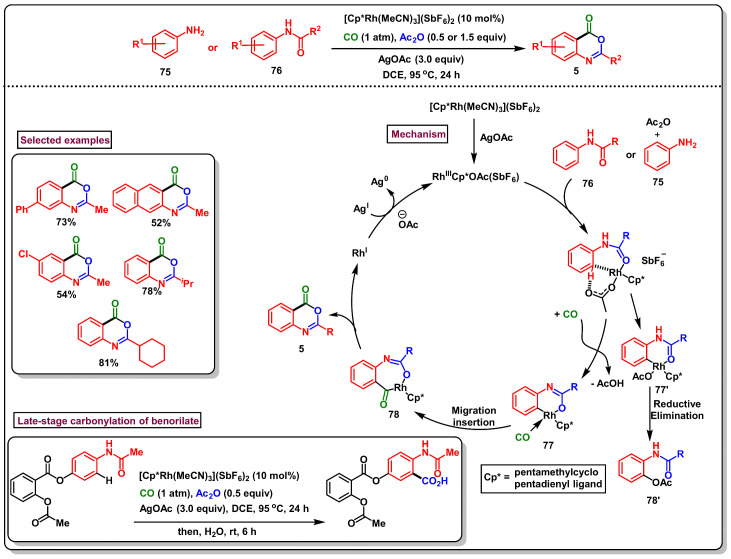
Rh(III)-catalyzed ortho-carbonylation strategy to prepare benzoxazine-4-ones.

By combining the C-H activation strategy and the utility of CO as a carbonyl source, a Rh(III)-catalyzed ortho-carbonylation of aniline **(75)** and its derivatives **(76)** to construct benzoxazine-4-ones **(5)** in a highly atom-economical one-pot manner was reported (Scheme 24) [76]. Notably, the acetanilides were generated in situ when the anilines were treated with excess Ac_2_O and simultaneously directed the ortho-C-H carbonylation to afford the corresponding benzoxazin-4-ones **(5)** via [3+2+1] cyclization. The reversibility of the C-H activation step was confirmed by kinetic isotope studies and deuteration experiments. The observed KIE value (1.04) revealed that the migratory insertion of CO was the rate-determining step. Acetic anhydride was effectively used as an additive to promote the reaction towards higher yields. Various meta-substituted precursors (-Me, -OMe, -*i*Pr, -Ph, -Br, -Cl) led to a single product in moderate to good yields; however, in the case of the meta-fluorinated substrate, regioisomeric products were obtained, owing to the minimal steric effect displayed by the F atom. The strong electron-withdrawing -CF_3_ unit provided the desired product in low yields (15%). Notably, diverse ranges of alkyl and cyclohexyl units were incorporated at the C2 position. Mechanistic studies showed that the coordination of the active Rh-catalyst to amide carbonyl followed by the C-H activation resulted in the Rh complex **(77)**. Further CO interaction and migratory insertion yielded the seven-member Rh-complex **(78),** from which **(5)** was released via reductive elimination. The reoxidation of Rh(I) to Rh(III) was effected by the silver salt. Rh complex **(77′)** formed at elevated temperature, resulting in by-product **(78′)** through reductive elimination. The potential of this method was further demonstrated in a late-stage functionalization of benorilate, an anti-inflammatory drug (Benorilate).

**Scheme 25:**
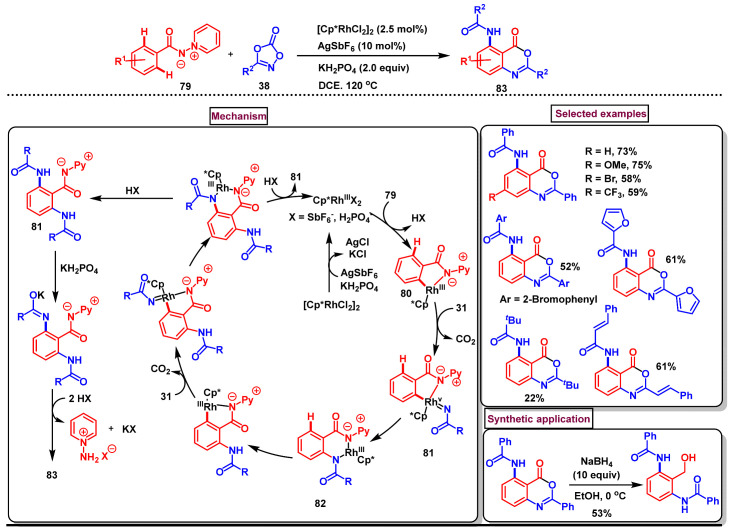
Rh(III)-catalyzed synthesis of 1.3-benzoxazin-4-ones using a removable directing group.

Directing the group-assisted formation of the C-N bond under the transition-metal-catalyzed conditions is an attractive tool for the construction of *N*-heterocycles in a high-atom and step-economic fashion [77]. To this end, Ma’s group described a novel Rh(III)-catalyzed diamidation followed by an intramolecular cyclization reaction of *N*-iminopyridinium ylides **(79)** and dioxazolones **(38)** to access benzoxazine-4-ones **(83)** (Scheme 25) [78]. *N*-iminopyridinium ylides served as a removable directing group in this process. Notably, the exclusive formation of the uncyclized diamidated product **(81)** was observed at room temperature. The electronic nature of the substituents on the *N*-iminopyridinium ylides had no obvious influence on the outcome of the reaction. Notably, in the case of meta-OMe- and -F-substituted substrates, the diamidated products were formed in moderate yields (38–43%). However, mono-amidated products were exclusively formed with other meta substituents such as -Me, -Br, and OCF_3_ (25–93%). On the other hand, this amidation/cyclization sequence worked well with the dioxazolones-bearing alkyl, aryl, heteroaryl and cinnamyl units. The initial formation of rhodacycle **(80)** from the *N*-iminopyridinium ylide **(39)** was followed by the coordination of **(38)** and resulted in the formation of **(81)** with the liberation of CO_2_. The further migratory insertion of nitrene led to **(82)**. Another set of second ortho -H activation, interaction with dioxazolone, migratory insertion, and protonation yielded the diamidated product **(81),** whereas the desired product **(83)** was obtained via tautomerization and intramolecular cyclization in the presence of KH_2_PO_4_. A scale-up experiment and the post-synthetic applications were demonstrated to describe the usefulness of this method. 

**Scheme 26:**
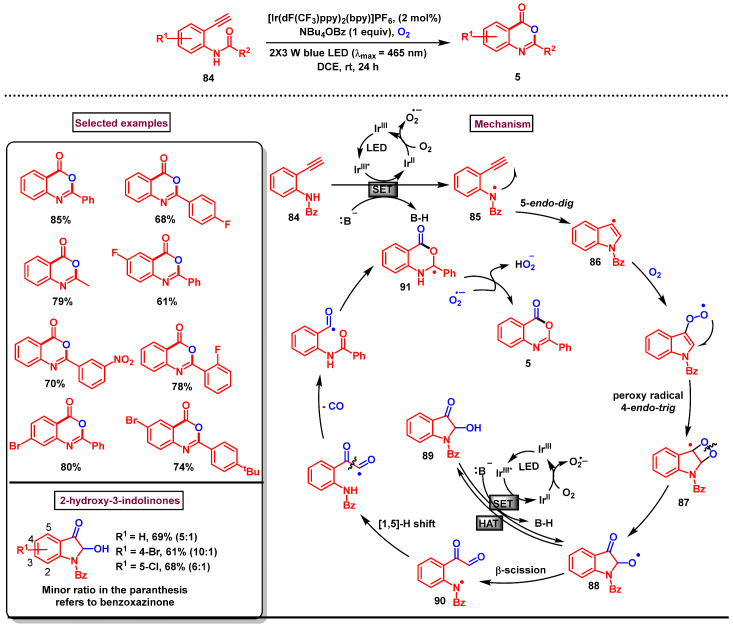
Ir-catalyzed photocatalytic *N*-radical-5-*endo*-dig cascade cyclization to prepare benzoxazine-4-ones.

Unlike the addition of a *N*-centered radical to alkenes, scant reports are known for the *N*-radical addition alkyne precursors, owing to the lower stability of vinyl radicals and the high-bond energy of alkynes. To this end, the first example of *N*-radical-5-*endo*-dig cascade cyclization via the addition of a *N* radical to alkyne was reported by Sun and Ye et al. (Scheme 26) to access a synthetically useful array of benzoxazine-4-ones **(5)** under Ir-catalyzed photo redox conditions [79]. Moreover, 2-arylated and 2-alkylated benzoxazine-4-ones were conveniently obtained from the corresponding alkynyl amide substrates. However, 2-heteroaryl-bearing substrates and internal alkynes were found inactive under these conditions. On the other hand, the aryl ring with substituents adjacent to an alkyne moiety resulted in the desired products in lower yields (51%) due to the steric influence. Theoretical calculations and various control experiments were performed to showcase the mechanistic rationale for this transformation. Excited state Ir^III^* and Bronsted base transformed the alkyne **(84)** into *N*-radical **(45)**, which underwent cyclization to yield the vinyl radical **(86)**. Further trapping with molecular oxygen and a 4-*endo-trig* radical cyclization resulted in intermediate **(87)**. Cleavage of the O-O bond followed by a β-scission yielded the *N*-radical **(90)**. Decarbonylation, intramolecular radical cyclization, provided the intermediate **(91)**, from which the product **(5)** was formed through the interaction of the superoxide anion radical. It should be noted that intermediate **(88)** can be converted into 2-hydroxy-3-indolinones **(89)** via the *H*-abstraction, and the process can be reversed under photocatalytic SET conditions. Molecular oxygen was necessary to regenerate the photocatalyst. The synthetic potential was demonstrated by a gram-scale synthesis and the preparation of biologically important quinazolines. In addition, 2-hydroxy-3-indolinones were also obtained under slightly modified reaction conditions. Overall, this radical cascade cyclization process enabled the controlled synthesis of benzoxazine-4-ones and 2-hydroxy-3-indolinones under mild conditions and exhibited an excellent substrate scope.

## 5. Preparation of Benzo[*d*][1,3]-Oxazin-4-Ones from Ortho-Aryl Halides

**Scheme 27:**
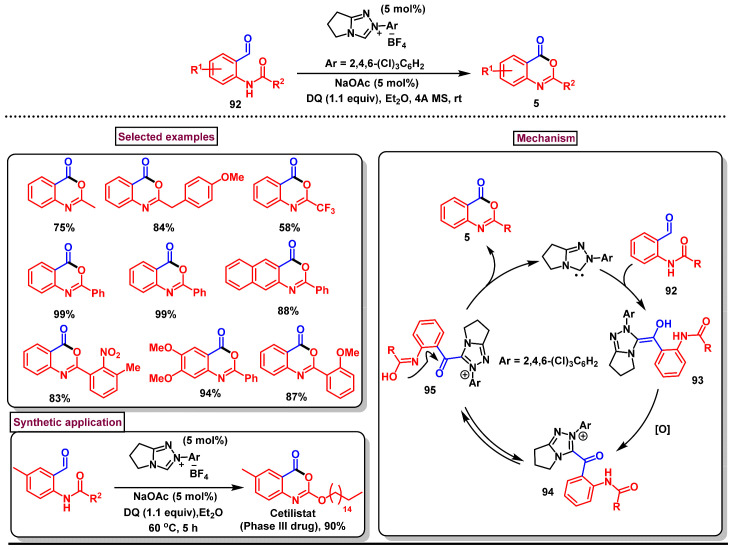
Preparation of benzoxazine-4-ones via NHC-catalyzed isomerization/cyclization.

An unprecedented assembly of benzoxazine-4-ones **(5)** via an oxidative NHC-catalyzed isomerization/cyclization tandem strategy from *N*-(2-formylphenyl)benzamide **(92)** was disclosed (Scheme 27) [80]. Synthetically valuable benzoxanin-4-ones were prepared, with excellent functional group tolerance and a broad substrate scope. Interestingly, the poly-aromatics, heterocyclic units, alkyl, alkyne, alkene, amine, ester, and ether functionalities on the amide unit were well-tolerated, and the corresponding products were obtained in excellent yields (up to 99%). Notably, chiral *N*-Boc amino acids and H were installed at the C2-position from the respective amide derivatives. On the other hand, aryl aldehydes, bearing substituents such as, halogens, -Me, -OMe and fused aromatics, were found compatible in offering benzoxazine-4-ones in excellent yields (up to 97%). Drug compounds such as Methaqualone, Cetilistat, Piriqualone, and the precursor for Idelalisib were prepared to showcase the synthetic potential of this method. In addition, a gram-scale synthesis further proved the utility of this method. A plausible mechanism suggested is that the Breslow intermediate **(93)** formed from the addition of the NHC (carbene) catalyst to **(92)** was oxidized to azolium species **(94)**. Further isomerization and intramolecular cyclization of **(95)** yielded the product **(5)**.

**Scheme 28:**
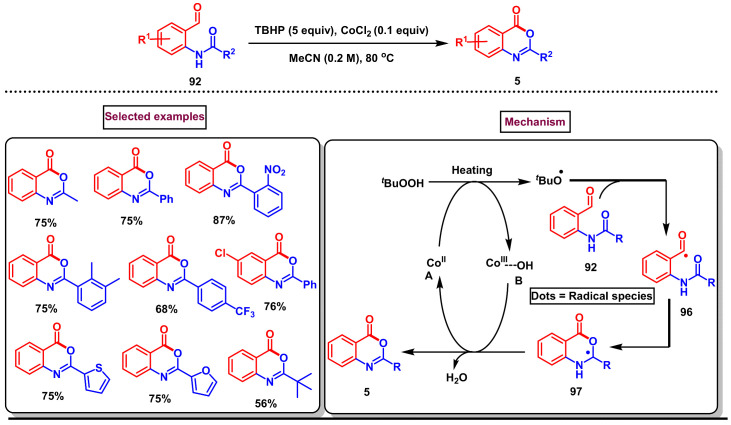
TBHP/CoCl_2−_mediated intramolecular oxidative cyclization to prepare benzoxazine-4-ones.

By utilizing similar substrates, an alternative approach via oxidative intramolecular cyclization to prepare benzoxazine-4-ones **(5)** using TBHP/CoCl_2_ catalytic system was developed by Du et al. (Scheme 28) [81]. This method was proceeded through oxidative C-O bond formation under mild conditions. A range of aryl and heteroaryl amides reacted well in offering the products in good yields (up to 87%). It was observed that bulkier alkylamides such as *tert*-butyl substituent resulted in the desired product in moderate yields (56%), whereas smaller alkyl groups led to no product, though the carboxylic acids were obtained in excellent yields (82–85%). The radical process was confirmed by a TEMPO-trapping experiment and a reasonable mechanism was also postulated. Accordingly, an initial H-abstraction of a *tert*-butoxy radical from **(92)** led to acyl radical **(96),** which through intramolecular radical cyclization offered **(97)**. Finally, another H-abstraction by high-valent Co^III^ released the product **(5)**.

**Scheme 29:**
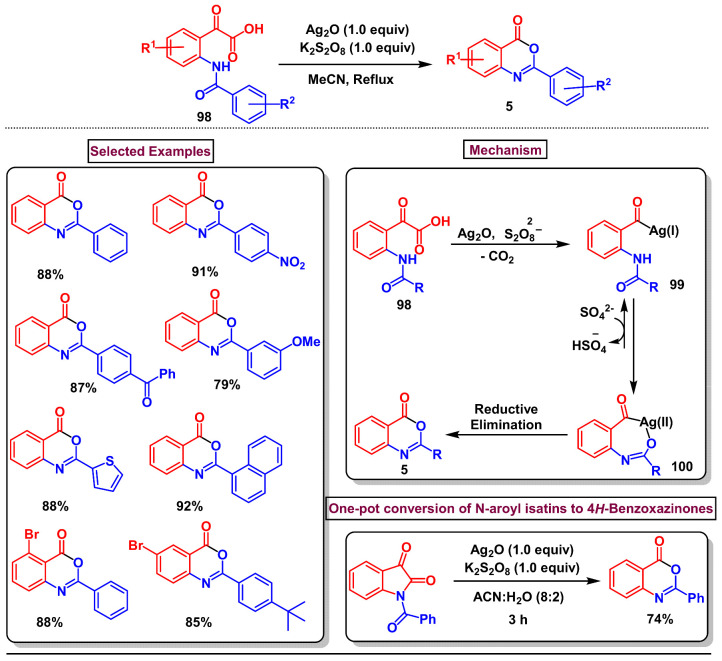
Ag-catalyzed intramolecular decarboxylative *O*-acylation of α-keto acids.

The facile access of aromatic ketones via decarboxylative acylation in an intermolecular fashion is well known. However, the intramolecular decarboxylative *O*-acylation of α-keto acids for accessing the fused *N*-heterocyclic skeleton is unexplored. To this end, a new method was reported to access functionalized benzoxazine-4-ones **(5)** from 2-arylamido-α-oxocarboxylic acid **(98)** via a silver-mediated decarboxylative intramolecular *O*-acylation with α-oxocarboxylic acid (Scheme 29) [82]. Irrespective of the electronic nature of the substituents on the aryl ring, the desired benzoxazine-4-ones were obtained in good yields (79–91%). Similarly, the amide moiety embedded with aryl, heteroaryl, and 1-naphthyl units showed good reactivity and offered the products in excellent isolated yields (up to 92%). It was observed that 2-alkylated-benzoxazin-4-ones were simultaneously converted into the corresponding 2-amido anthranilic acids owing to the instability. A one-pot in situ hydrolysis of *N*-aroyl isatin and the sequential decarboxylative *O*-acylation were also realized to afford benzoxazine-4-one **(5)** in good yields. Water served as a hydrolytic agent to produce α-oxocarboxylic acid under one-pot conditions. Mechanistically, this transformation proceeded via the sequential formation of silver (I) **(99)** and silver (II) **(100)** complexes through the interaction of substrate **(98)** with Ag_2_O/K_2_S_2_O_8_. The reductive elimination of **(100)** delivered the product **(5)**. To demonstrate the suitability of the large-scale outcome, a gram-scale experiment was performed, and the product was isolated in 87% yield.

**Scheme 30:**
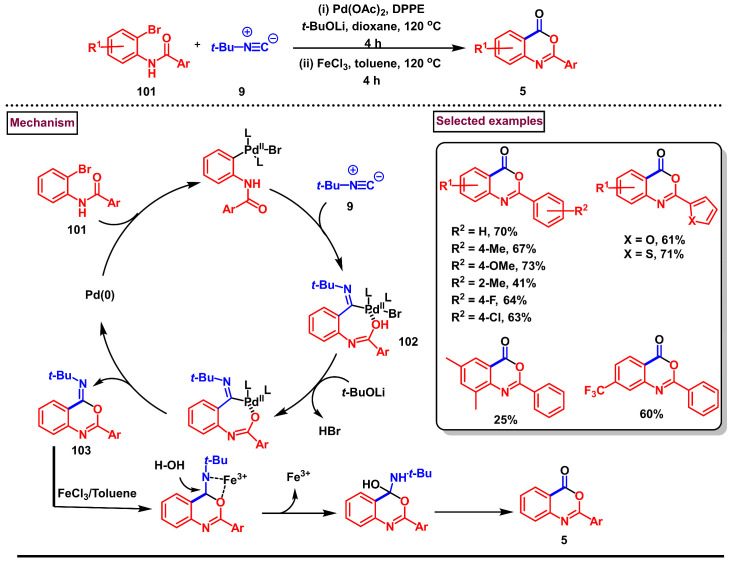
Pd-catalyzed synthesis of benzoxazine-4-ones via isocyanide insertion.

As a versatile C1 source, isocyanides provide easy access to the formation of C-N bonds. However, the subsequent protic acid-mediated hydrolysis of the imine intermediates to carbonyl compounds lead to a significant formation of undesired products. Adopting FeCl_3_-promoted hydrolysis, a Pd-catalyzed approach to access 4*H*-benzo[*d*][1,3]oxazin-4-ones **(5)** was developed from amide **(101)** via isocyanide insertion (Scheme 30) [83]. Multi-substituted aryl and heteroaryl moieties embedded in an amide unit containing functional groups were studied. (41–73%). The sterically hindered ortho-substitution on the aryl group of the amide moiety resulted in lower yields (41%). Mechanistically, the oxidative addition of **(101),** followed by isocyanide insertion, led to Pd-complex **(102)**. Base-mediated dehydrohalogenation, followed by a reductive elimination, afforded the imine intermediate **(103)**. The FeCl_3_-assisted hydrolysis of imine generated the desired product **(5),** with the simultaneous elimination of triethylamine. Interestingly, *N*-(2-cyanophenyl)benzamides were obtained through AlCl_3_-catalyzed hydrolysis, with the elimination of *t*-Bu unit. 

**Scheme 31:**
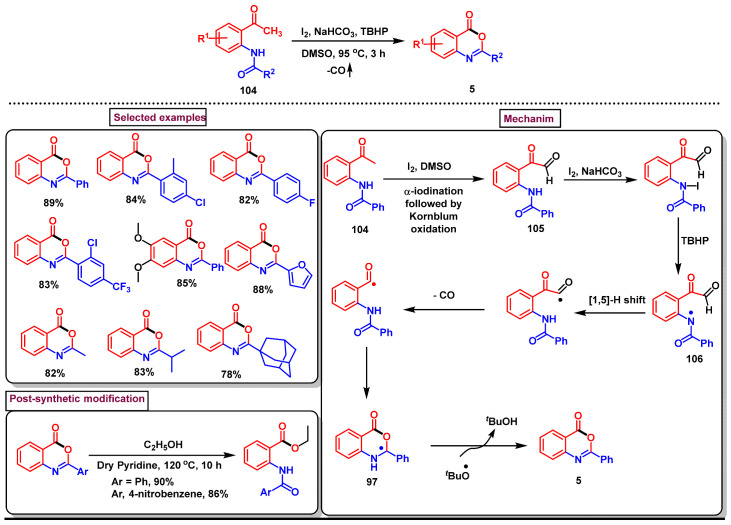
I_2_/TBHP-mediated synthesis of benzoxazine-4-ones via decarbonylation.

The I_2_/TBHP-mediated selective cleavage of the C(*sp^3^*)-C(*sp^2^*) bond for the construction of the C-O bond was realized to access benzoxazine-4-ones from readily available *N*-(2-acetylphenyl)aryl or alkylamides **(104)** (Scheme 31) [84]. This decarbonylative coupling strategy was explored with a variety of electronically and sterically distinct substituents, and the corresponding benzoxazine-4-ones **(5)** were obtained without significant variation in the isolated yields. There was no profound steric effect observed when ortho-substituted benzamides were employed, and the desired products were obtained in excellent yields (up to 90%). Specifically, this transition-metal-free decarbonylative process worked especially well with heteroaryl (2-furyl, 2/3-thiophenyl), and alkyl (1^o^, 2^o^, 3^o^) amides, and the anticipated products were isolated in appreciable yields (up to 90%). Importantly, biaryl benzoxazine-4-ones, amenable for metal complexation, were also achieved in 87% isolated yields. Further, a gram-scale synthesis and conversion of the benzoxazine-4-ones to synthetically useful ethyl-2-arylamidobenzoate demonstrated the synthetic potential of this oxidative decarbonylative strategy. Various intermediates involved in this transformation were confirmed by HRMS analysis. The mechanistic rationale revealed that the sequential α-iodination and Kornblum oxidation of **(104)** led to oxoacetyl intermediate **(105)**. Further *N*-iodination and TBHP enabled the formation of amidyl radical **(106)**. A series of 1,5-*H* shift, CO elimination, and intramolecular radical cyclization resulted in **(97)**. Product **(5)** was formed via H-abstraction.

**Scheme 32:**
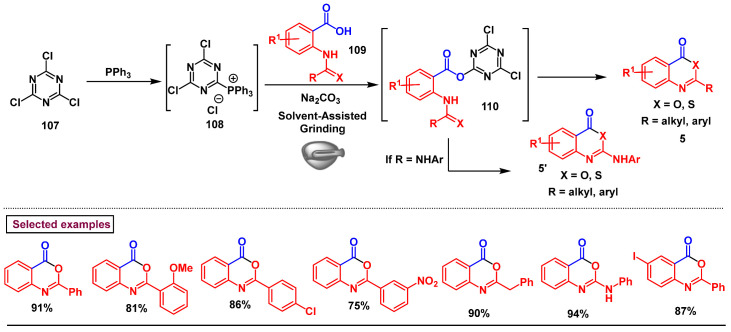
Rapid cyclodehydration of *N*-acyl anthranilic acid via a mechanochemical approach.

In comparison to traditional heating or microwave-assisted methods, mechanochemical routes are often performed under mild conditions with shorter reaction time. To this end, a solvent-assisted grinding approach for the synthesis of a series of 2-substituted benzoxazine-4-ones **(5)**, was reported by Pattarawarapan et al. (Scheme 32) [85]. The rapid cyclodehydration of *N*-acyl anthranilic acid **(109)** was realized in the presence of 2,4,6-trichloro-1,3,5-triazine (**107**, Dehydrating agent). Optimizational studies revealed that the use of *N*-Methylmorpholine lowered the product yield (55%), possibly due to the formation cum decomposition of *N*-triazinylammonium intermediate. The presence of electron-withdrawing substituents (halogens, -NO_2_) on either aryl ring (anthranilic acid or benzamide) slightly lowered the product yield (up to 56%). However, the electronic effects of the substituents were found to be less crucial. It must be noted that highly sensitive and unstable 2-alkylated benzoxazine-4-ones (prone to hydrolysis) were obtained in high yields (up to 90%). Shorter reaction time, mild conditions, and high yields are the notable features of this report. A detailed optimizational study revealed that the presence of PPh_3_ enabled the in situ formation of triazinylphosphonium chloride **(108),** which activated the substrate, leading to rapid cyclization. This method was further extended to the synthesis of 2-aminobenzoxazin-4-ones **(5)** and 2-aminobenzothiazine-4-ones **(5′)** from the cyclization of carbamides or thiocarbamides.

**Scheme 33:**
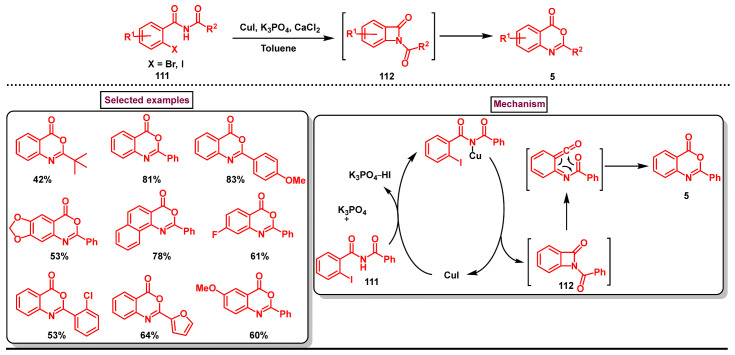
Cu-catalyzed intramolecular C-N coupling/rearrangement to prepare benzoxazine-4-ones.

Zhu et al. developed an intramolecular tandem C-N coupling/rearrangement sequence of *N*-acyl-2-halobenzamides **(111)** to prepare benzoxazine-4-one derivatives **(5)** (Scheme 33) [86]. Under the optimized conditions, the scope was evaluated with a variety of substrates. Both electron-withdrawing and electron-releasing groups led to the desired products with good to excellent yields. A series of alkyl, aryl, alkenyl, styryl, and heteroaryl groups on R^2^ were found compatible in yielding the desired benzoxazine-4-ones in moderate to good yields (36–81%). Similarly, the electron-donating (-Me, -OMe) and withdrawing groups (-Cl, -F) R^1^ resulted in excellent product formation (79–87%). Slightly lower yields were observed when the substrates were changed into bromo analogues. Activation of **(111)** by CuI and intramolecular C-N coupling resulted in the *N*-benzoyl benzazetone **(112)**. Ring opening by cleavage of the C-N bond, followed by ring formation through the C-O bond, yielded the product **(5)**.

**Scheme 34:**
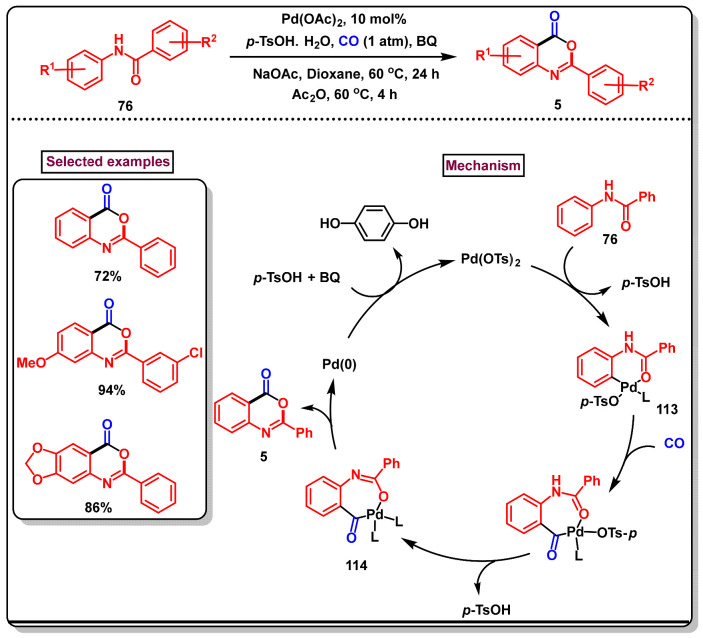
Pd-catalyzed sequential C-H activation/carboxylation of anilides to access benzoxazine-4-ones.

The Pd-catalyzed sequential C-H activation/carboxylation of anilides was demonstrated to achieve a series of biologically relevant benzoxazine-4-ones **(5)** in excellent yields (Scheme 34) [87]. In this pioneering and atom-economic transformation, the acyl group acted as a directing group via C-H activation and was further incorporated in the structure of **(5)**. OTs attached the active Pd-catalyst coordinated to **(76)** via C-H activation and resulted in the key cyclopalladated intermediate **(113)**. Further insertion of CO and the elimination of *p*-TsOH provided the seven-member Pd-complex **(114)**. Product **(5)** was formed through reductive elimination. Additionally, biologically significant quinazolinones were prepared by treating *N*-acyl arylcarboxylic acids with aniline in the presence of PCl_3_.

**Scheme 35:**
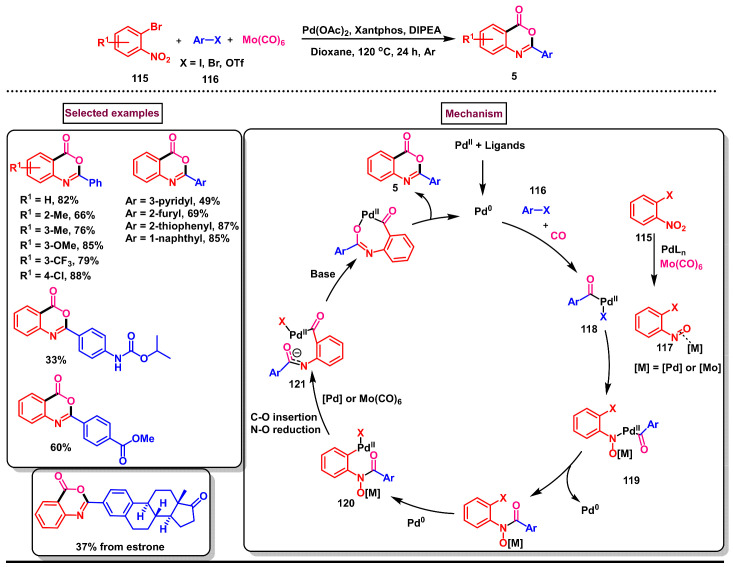
Pd-catalyzed reductive double carbonylation of nitroarenes using Mo(CO)_6_.

Metal carbonyls mediating the reductive carbonylation of nitroaryl compounds is an established chemical principle, whereas reductive double carbonylation using metal carbonyls is challenging but useful in achieving increased molecular complexity. To this end, Zhang and Yin et al. both reported a Pd-catalyzed process to access benzoxazine-4-ones **(5)** from nitroarenes **(115)** and aryl halides **(116)** via a Mo(CO)_6_-mediated reductive double carbonylation strategy (Scheme 35) [88]. Nitroarenes **(115)** bearing ether, ester, and halogens were smoothly involved in this transformation to afford the desired product in good isolated yields (up to 88%). Notably, the -Br group on the aryl ring remained unaffected. Similarly, the aryl and heteroaryl iodides **(116)** possesing amide, ether, ester, and halogens were amenable. Alternatively, aryl bromides and trifluoromethanesulfonates were also involved in this reaction and provided the corresponding products in acceptable yields (up to 80%). However, only *N*-(2-chlorophenyl)benzamide was detected in the case of aryl chlorides. Based on the control experiments, a plausible mechanism was proposed. Accordingly, the initially formed acylpalladium complex **(118)** was reacted with nitroso compound **(117)** (formed from nitro reduction) to afford the complex **(89)**. The sequential reductive elimination and oxidative addition of C-X bond resulted in intermediate **(120)**, which upon CO insertion and reduction in the N-O bond led to anionic intermediate **(121)**. The base-promoted intramolecular nucleophilic cyclization and release of Pd(0) via reductive elimination afforded the desired benzoxazine-4-one **(5)**. Interestingly, in this process Mo(CO)_6_ served as a stable carbonyl source and reductant. A late-stage synthetic modification of estrone was also demonstrated to showcase the synthetic utility of this method.

## 6. Preparation of Benzo[*d*][1,3]-Oxazin-4-Ones from Benzoic Acid Derivatives

**Scheme 36:**
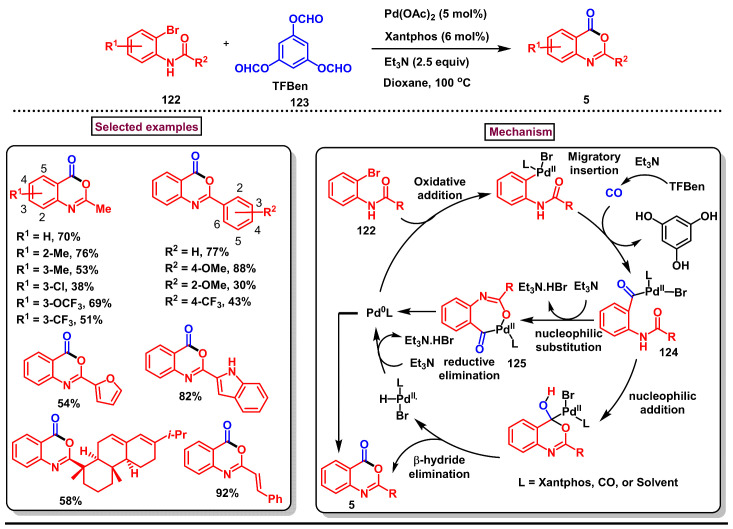
Pd-catalyzed carbonylation of amides using benzene-1,3,5-triyltriformate.

By using benzene-1,3,5-triyltriformate (TFBen) as a non-gaseous CO surrogate, a Pd-catalyzed carbonylative cross-coupling of *N*-(ortho-bromoaryl)amides **(122)** was reported to access functionalized 4*H*-benzo[*d*][1,3]oxazin-4-ones **(5)** (Scheme 36) [89]. Variously substituted *N*-(2-bromoaryl)acetamides were prepared from the ortho-bromo anilines via acetylation and subjected to this CO-free carbonylative process. It was observed that 2-alkyl (1^o^, 2^o^, 3^o^), aryl, heteroaryl (furanyl, indolyl), and styryl functionalities could be incorporated in moderate yields (21–92%). The electron-withdrawing groups (-Cl, -Br, -CF_3_) on the aryl ring of the amide precursor lowered the product formation (21–53%). The synthetic advancement of this method was further proved by converting the structurally complex abietic acid and indomethacin into the corresponding benzoxazine-4-ones via their corresponding amides. This method exhibited excellent functional group compatibility. The oxidative addition of Pd(0) to substrate **(122),** followed by CO insertion, generated the acyl palladium complex **(124)**. The intramolecular cyclization of the amido group to the Pd-acyl unit and the β-hydride elimination released the desired product **(5)** and regenerated Pd(0). Alternatively, **(124)** could undergo base-promoted intramolecular cyclization **(125)** and reductive elimination to afford the target product. The utilization of non-toxic CO surrogate, wide substrate scope and diverse range of post-synthetic modifications are the meritorius features of this method.

**Scheme 37:**
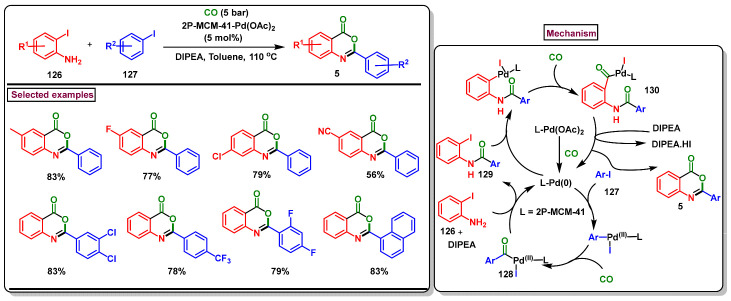
Heterogeneous Pd-catalyzed double carbonylation to prepare benzoxazine-4-ones.

The Pd-catalyzed carbonylative synthesis of benzoxazine-4-ones is a proven strategy. Most of the known routes utilize homogeneous Pd-complexes which are difficult to recover and often lead to undesired coordination with the heterocyclic products. To circumvent this issue, Cai et al. developed a sustainable double carbonylation procedure involving a heterogeneous Pd-catalyst to construct 2-arylbenzoxazin-4-ones **(5)** from readily available aryl iodides **(127)** and 2-iodoanilines **(126)** (Scheme 37) [90]. The 2P-MCM-41-Pd(OAc)_2_ complex was utilized as the catalyst, while the mesoporous MCM-41 was employed as support for immobilization due to its large surface area. The attractive features of this double carbonylation process are the remarkable functional group tolerance and recovery of the Pd-catalyst (via simple filtration process) without a loss in catalytic efficiency. Aryl and heteroaryl iodides **(127),** bearing strong electron-withdrawing groups such as -NO_2_, -CN, provided the product in lower yields (40–46%). Halogens (-Cl, -Br) on the aryl iodides remained unaffected during this carbonylative cyclization. Sterically hindered ortho-substitutions (-Me, -OMe) and 1-naphthyl groups were less impactful, and the corresponding products were obtained in good yields (up to 83%). In the case of 2-iodoanilines **(126)**, electron-donating groups and halogens favored the reaction; however, the -CN group drastically decreased the product yield to 56%. The ICP-AES analysis proved that no Pd species were detected in the filtrate, confirming the heterogeneous nature and stability of the 2P-MCM-41-Pd(OAc)_2_ catalyst. Mechanistically, Pd(II) was reduced to reactive Pd(0). The further oxidative addition of **(127)** and the CO insertion provided the acyl Pd-complex **(128)**. By reacting with aniline **(126)**, amide intermediate **(129)** was formed. Another set of oxidative addition with the regenerated Pd(0), insertion of CO, and intramolecular cyclization of acyl Pd-complex **(130)** released the product **(5)**.

**Scheme 38:**
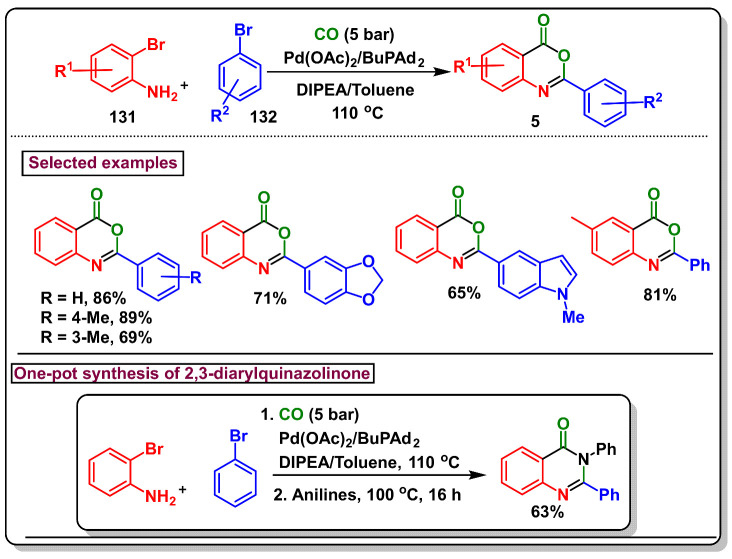
Homogeneous Pd-catalyzed double carbonylation to prepare benzoxazine-4-ones.

A very similar homogeneous Pd-catalyzed double carbonylation strategy (Beller et al. 2012) was reported by Beller’s group from bromoanilines **(131)** and bromoarenes **(132)** (Scheme 38) [91]. Aryl and heteroaryl bromides with ether, ester, halogens, and -CF_3_ were found equally reactive, and the corresponding products were obtained in moderate to good yields. (65–91%). Phenyl triflate was decomposed under standard conditions. On the other hand, electron deficient 2-bromoanilines were less reactive, and they afforded only 5–10% of the product. The synthetic efficiency of this double carbonylation process was further showcased by accessing 2,3-diarylquinazolines directly in one-pot. A mechanistic proposal similar to Scheme 37 was elucidated. 

**Scheme 39:**
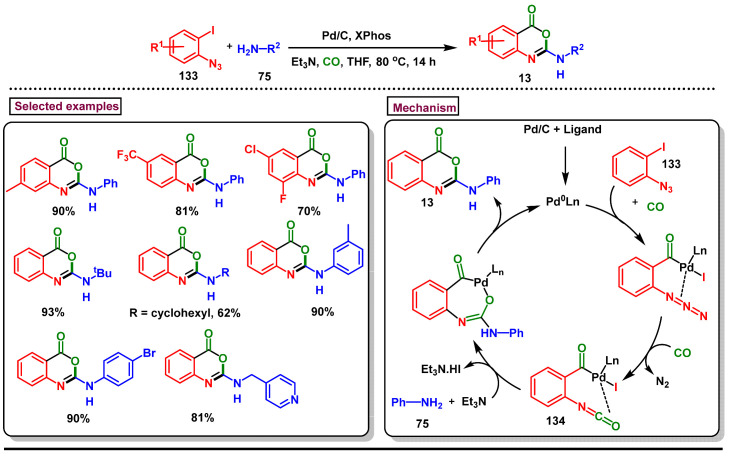
Preparation of 2-aminobenzoxazin-4-ones via Pd-catalyzed carbonylative sequence with aryl azides.

The utility of amines **(36)** and 2-iodoaryl azides **(133)** in the preparation of 2-amino benzoxazinones **(13)** via a Pd-catalyzed carbonylative sequence was demonstrated by Wu et al. (Scheme 39) [92]. Compared to other known methods, using azide precursors is advantageous, as no external oxidants are needed, generating N_2_ as a byproduct. With very low Pd-catalyst loading (1 mol%), the desired products were obtained in excellent yields (up to 96%). Differently substituted anilines **(75)** with functional groups such as -Br, -F, -CF_3_, -OMe, and -Ph were tested, and the desired products were obtained in the range of 49–96% isolated yields. In general, electron-donating groups displayed higher reactivity. Additionally, aliphatic amines tethered with alkyne, alkene, and heteroaryl groups were also amenable, and the 2-functionalized benzoxazine-4-ones were achieved in 25–81% of yields. On the other hand, 1-azido-2-iodobenzenes **(133)** displayed moderate generality and offered the products **(13)** in excellent yields (70–95%). A typical oxidative addition of **(133)** with in situ-generated Pd(0), followed by CO insertion, gave isocyanate **(134)**. The further nucleophilic reaction of amine, intramolecular cyclization, and reductive elimination yielded the product **(13)**. Application was demonstrated by performing a gram-scale synthesis (91%) and preparing a biologically important PSA-specific inhibitor in 88%.

**Scheme 40:**
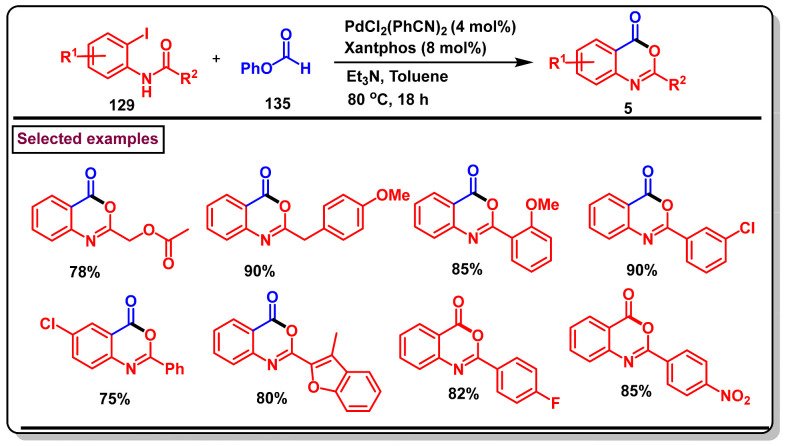
Pd-catalyzed carbonylative cyclization of 2-iodoanilides utilizing phenyl formate.

As a pseudohalide and CO surrogate for numerous carbonylative approaches, aryl formats are well known bifunctional reagents. To this end, a highly efficient Pd-catalyzed carbonylative cyclization of 2-iodoanilides **(129)** utilizing phenyl formate **(135)** was developed to prepare benzoxazine-4-ones **(5)** (Scheme 40) [93]. Irrespective of the substitution pattern and electronic nature, various 2-iodoanilides, bearing -NO_2_, -halogens, -esters, benzyl, and heteroaryl groups, were smoothly converted into the desired benzoxazine-4-ones in excellent yields (up to 90%). Biologically important 2-benzofuran-fused benzoxazine-4-ones **(5)** were also obtained under the optimized conditions in higher yields (80%). In addition, the authors have also reported a phosphine and solvent free method for the preparation of amino acid-derived phthalimides.

**Scheme 41:**
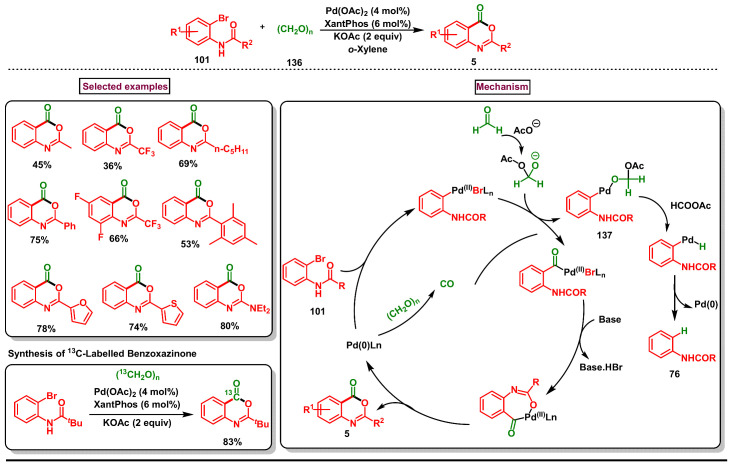
Pd-catalyzed carbonylative cyclization of 2-bromoanilides using paraformaldehyde.

Another relevant approach using paraformaldehyde **(136)** as a safe carbonyl source was reported by Wu et al. (Scheme 41) [94]. Detailed optimizational studies were carried out and Pd(OAc)_2_/XantPhos were found to be the best system. When the acyl group of *N*-(*o*-bromoaryl)amide was substituted with -*t*Bu group, the desired product was obtained in a 86% yield. However, methyl or the CF_3_-substituted acyl group led to *N*-phenylacetamide as a major product with simultaneous debromination. In the case of longer alkyl chain, cyclic alkyl, aryl, and heteroaryl groups, the corresponding products were obtained in good to excellent yields (63–80%). Notably, formamide analogues were unreactive and led to no desired products. It should be noted that arylacyl, bearing *o*-bromoanilines, required a slightly high temperature to proceed. On the other hand, electron-withdrawing groups on the aryl unit of *N*-(*o*-bromoaryl)amide considerably lowered the yields. A one-pot attempt from the aniline derivative was unsuccessful. By utilizing ^13^C-labeled paraformaldehyde, pharmaceutically important 4-^13^C-benzoxazinone was isolated in 83% yields. In situ generation of CO and its incorporation was confirmed by using ^13^C-labeled paraformaldehyde. The corresponding 4-^13^C-benzoxazinone was isolated in 83% yields. The mechanistic pathway was similar to that discussed in Scheme 36. Notably, the β-hydride elimination of the Pd-complex led to **(76)** via the unstable hydroaryl Pd-complex.

**Scheme 42:**
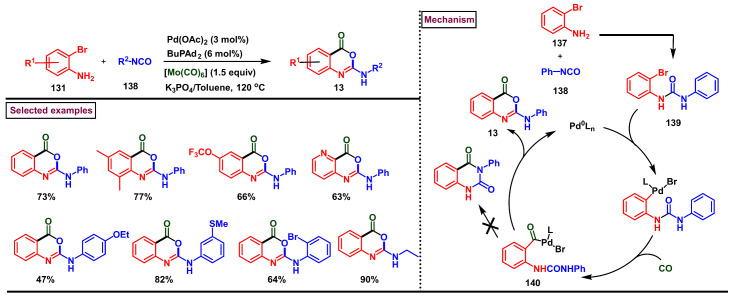
Pd-catalyzed carbonylation approach using isocyanates and 2-bromonilines.

In continuation of the development of novel Pd-catalyzed methods for the synthesis of 2-functionalized benzoxazine-4-ones, Beller’s team prepared a series of 2-amino benzoxazinones **(13)** from readily available isocyanates **(138)** and 2-bromonilines **(131)** in the presence of [Mo(CO)_6_] as CO source (Scheme 42) [95]. Under the optimized conditions, various 2-bromoanilines, 3-bromopyridine-2-imine, 2-bromopyridine-3-amines were tested, and the desired products were obtained in moderate yields (63–77%). Similarly, aryl Isocyanates, bearing halogens, -CF_3_, -OCF_3_, -CN, -Et, and alkyl isocyanates, resulted in the formation of the corresponding 2-aminobenzoxazin-4-ones in acceptable yields (up to 90%). The Authors proposed that [Mo(CO)_6_] played a crucial Lewis acid role by coordinating with acyl-Pd-complex **(140)** and assisted the product formation. Interestingly, this method was also extended by treating 2-bromophenylisocyanates with anilines under similar conditions. Mechanistically, in situ-generated Pd^0^ was oxidatively added to the C-Br bond of urea derivative **(139)**. Further CO insertion and the reductive elimination of acyl-Pd-complex **(140)** led to product **(13)**. Another approach to prepare 2-functionalzied benzoxazine-4-ones was reported by using readily available acid anhydrides and 2-bromoanilines using gaseous carbon monoxide (2 bar) [96].

**Scheme 43:**
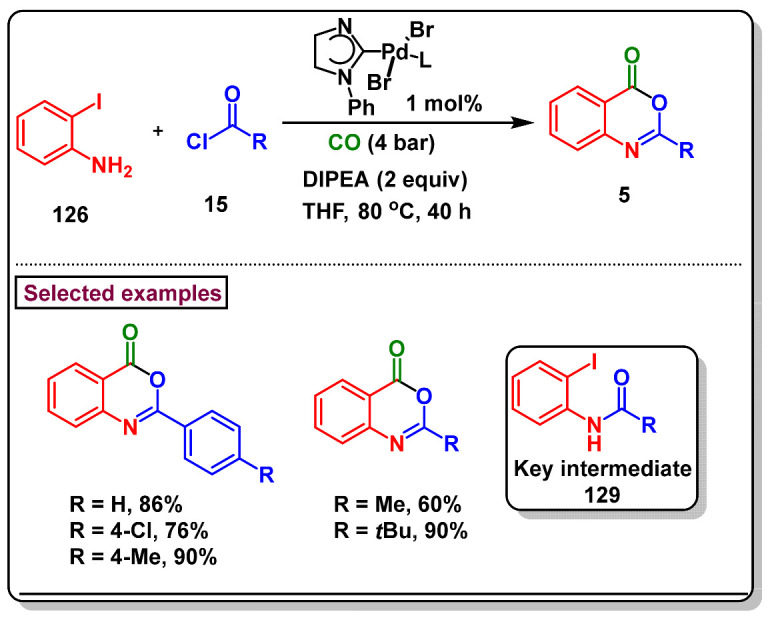
Pd-NHC-catalyzed carbonylative annulation of 2-iodoanilines, acyl chlorides.

A three-component Pd-NHC-catalyzed carbonylative annulation involving 2-iodoanilines **(126)**, acyl chlorides **(15),** and CO (4.0 bar) was reported to access 4*H*-1,3-benzoxazin-4-ones **(5)** (Scheme 43) [97]. At very low catalyst loading (1 mol%), the cyclocarbonylation proceeded efficiently to afford a wide range of benzoxazine-4-ones **(5)** in good yields (up to 90%). Both bulkier pivaloyl chlorides and aryl acid chlorides **(15)** afforded the desired products in excellent yields. It was observed that pivaloyl chloride exhibited superior reactivity than acetyl chloride and gave the desired benzoxazine-4-one **(5)** in 90% isolated yields. A noticeably lower yield was obtained in the case of benzoyl chloride with electron deficient halogens (76%), whereas the para-Me group led to 90% product formation. The authors have also performed a stepwise formation of 2-pivoloyl-benzoxazin-4-ones via the isolation of corresponding amide (88%). Control experiments proved the initial formation of amide derivative **(129),** which was further converted into the desired product **(5)** via the cyclocarbonylation sequence. In addition, the authors have also demonstrated the synthesis of flavones via the hydroxycarbonylation of iodobenzene with alkynes under Pd-NHC catalytic conditions. A similar kind of Pd/C-catalyzed cyclocarbonylation was also reported by Petricci et al. (2010) under microwave-assisted conditions [98]. The Pd/C-catalyzed CO insertion in this process was achieved by using gaseous carbon monoxide (CO), used at 170 psi. 

## 7. Preparation of Benzo[*d*][1,3]-Oxazin-4-Ones from Indoles and Isatins

**Scheme 44:**
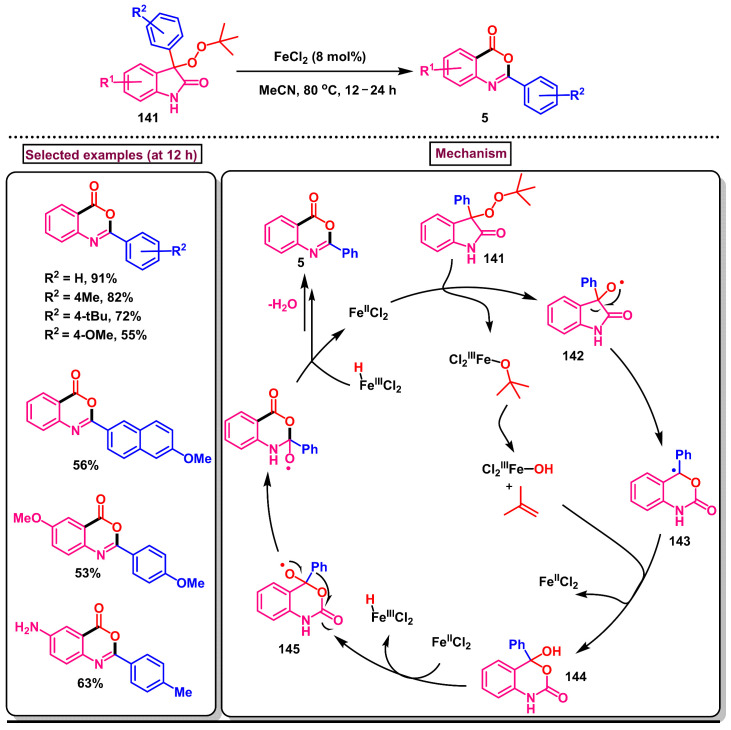
Synthesis of benzoxazine-4-ones via FeCl_2_-catalyzed radical rearrangement.

C3-functionalized peroxy indoles are useful building blocks and used in various organic transformations due to the sensitive peroxide bond. From C3-arylated peroxy indoles **(141)**, 1,3-benzoxzine-4-ones **(5)** were achieved through the FeCl_2_-catalyzed radical rearrangement (Scheme 44) [99]. Control experiments under N_2_ atmosphere suggested that the carbonyl oxygen of **(5)** was from either peroxy linkage or from amide carbonyl. Moreover, the detection of isobutylene further confirmed the homolytic cleavage of peroxide. This transformation did not proceed with Fe(III), whereas in situ-generated Fe(II) in the presence of hydroquinone provided the desired product **(5),** thus confirming the active role of Fe(II). C3-aryl peroxyoxindoles, possessing -OMe, -*t*Bu, -Ph, -Me, and -F, were rearranged to 2-arylated benzoxazine-4-ones in good yields (up to 92%). C3-naphthalenyl derivatives led to lower yields (53–56%), presumably due to steric effects. C3-methylated substrate resulted in C3-hydroxy indoles via the deprotection of the *t*BuO group; however, other alkylated groups (*n*Bu, *i*Pr) resulted the isatin derivatives via the Hock cleavage process. On the other hand, this radical rearrangement was found compatible with -Me, -Br, -NH_2_, -OMe substitutions on the core aryl ring of the peroxy indole. The proposed pathway revealed that alkoxy radical intermediate **(142)** was generated upon the coordination of Fe^II^Cl_2_ with the peroxide oxygen atom and cleavage of O-O bond. The addition of **(142)** to the amide carbonyl center through the C2-C3 bond cleavage led to radical **(143),** which was converted into hydroxy intermediate **(144)** upon the interaction with Fe^III^(OH)Cl_2_. The single electron transfer between **(145)** and in situ*-*formed Fe^II^Cl_2_ resulted in the acetal radical, in which aryl migration occurred to the amide carbonyl center via a radical relay process. Further H-transfer and elimination of H_2_O delivered the product **(5)** and regenerated FeCl_2_. A gram-scale synthesis and concise preparation of the elastage inhibitor were performed to highlight the synthetic utility.

**Scheme 45:**
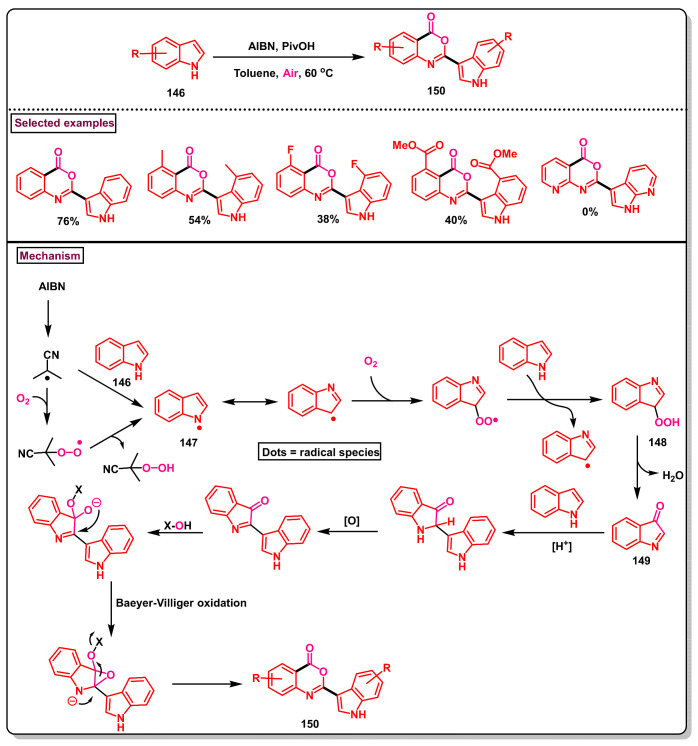
Preparation of 2-indolylbenzoxazin-4-ones via oxidative cleavage of C2−C3 bond.

Direct functionalization of 1*H*-indoles **(146)** to achieve 2-indolylbenzoxazin-4-ones **(150)** was realized in the presence of azobis(isobutyronitrile) (AIBN) under open air via oxidative cleavage of the C2-C3 bond (Scheme 45) [100]. The involvement of the radical process and the crucial role of AIBN and air were confirmed by a series of control experiments. Upon pre-termination in 2 h, the key intermediate 3*H*-indol-3-ones **(149)** was detected along with desired product **(150)** in HRMS. The profound steric impact was observed with substitutions at C4 or C7. When -F and -OMe groups were substituted at C7 of the indole precursors, corresponding products were obtained in 32% and 28% isolated yields. C4-Me and C4-F substitutions resulted the product formation in 54% and 38%. This oxidative recyclization failed with the pyridine-fused indole substrate. The proposed mechanism stated that the initial formation of indolyl radical **(147)** was observed via AIBN-mediated NH-abstraction of indole. Upon reaction with molecular oxygen, a C3-hydroperoxide radical **(148)** was formed and transformed into key intermediate 3*H*-indol-3-ones **(149)**. Acid-promoted nucleophilic addition of 1*H*-indole to **(149),** followed by a Baeyer–Villiger oxidation, resulted in the 2-indolylbenzoxazin-4-ones **(150)** through the cleavage of the C2-C3 bond. Owing to the wide occurrence of unprotected 1*H*-indoles, a broad range of multi-substituted benzoxazine-4-ones were obtained with excellent step and atom economy.

**Scheme 46:**
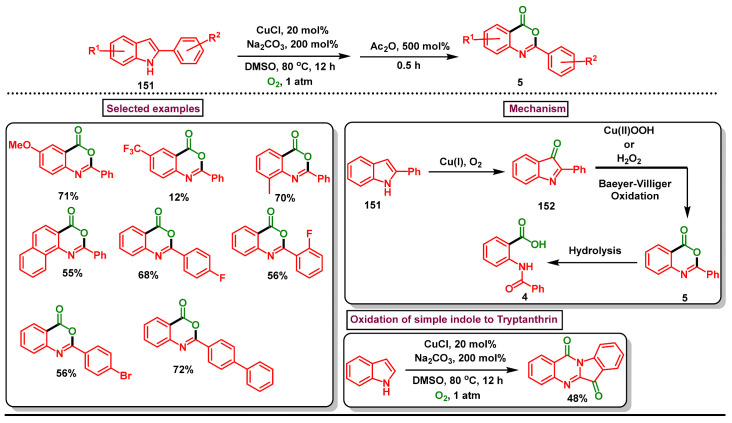
Cu-catalyzed oxidative synthesis of 2-arylated benzoxazine-4-ones.

Cu-catalyzed oxidative tandem synthesis of 2-arylated benzoxazine-4-ones **(5)** was reported from 2-arylindoles **(151)** in the presence of Cu(I)-salt and oxygen by Yamashita et al. [101]. The 2-alkyl indoles did not react under the reaction conditions; on the other hand, simple unsubstituted indoles were oxidatively converted into Tryptanthrin via Cu-catalyzed aerobic oxidation [102]. Notably, though *o*-alkynylanilines are known to yield 2-substituted indoles via Cu-catalyzed intramolecular annulation [103], under these reaction conditions, anthranilic acid derivative **(4)** was obtained as the sole product (Scheme 46). The reaction was quite general with variously substituted 2-aryl indoles **(151)**. The electron-donating substituents (-Me, -OMe) and halogens (-Br, -Cl, -F) were smoothly converted into 2-arylbenzoxazin-4-ones **(5)** in moderate yields (up to 82%). However, the 5-CF_3_ substituted indole precursor led to lower yields (12%), even with the excess Cu-catalyst. A fair substrate scope was demonstrated at the C2-aryl ring of the indole. A one-pot synthesis of **(5)** was also demonstrated from arylacetylenes and 2-iodoanilines via the tandem Sonagashira coupling, deprotective cyclization, oxidative hydrolysis, and cyclization of *N*-protected anthranilic acid intermediate. Mechanistic insights revealed that the aerobic oxidation of **(151)** under copper-catalyzed conditions led to indolone **(152)**. Further peroxide-mediated Baeyer–Villiger oxidation yielded the product **(5)**. Hydrolyzed product *N*-acyl anthranilic acid **(4),** observed under the reaction conditions, can be further converted into product **(5)** in the presence of Ac_2_O.

**Scheme 47:**
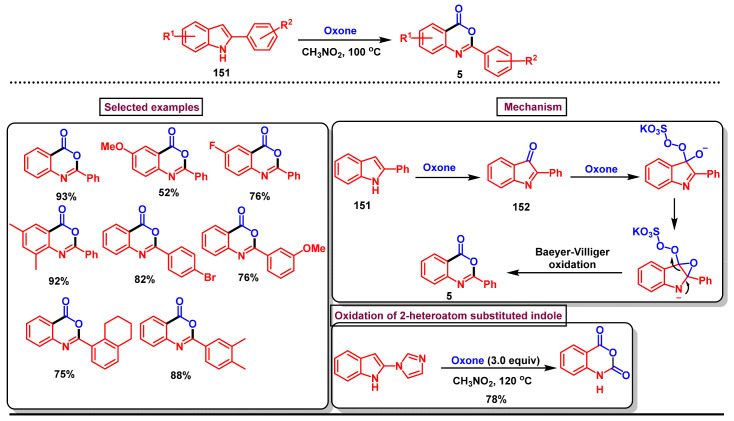
Direct oxidation of 2-aryl indoles into 2-arylated benzoxazine-4-ones using oxone.

Another example of direct oxidation of 2-aryl indoles **(151)** into 2-arylated benzoxazine-4-ones **(5)** was reported using oxone as a stable and non-toxic oxidant (Scheme 47) [104]. Sterically and electronically divergent substrates were screened, and the desired products were obtained in good to excellent yields (up to 93%). Common substituents such as -Me, -Cl, -Br, and -F worked well and high product yields were obtained (up to 90%). In the case of -OMe substitution, the partial decomposition of the product lowered the yield (52%). Variations in the C2-aryl group did not alter the outcome of the reaction. However, indol-2-yl pivalate produced indolin-2-ones (86%) and C2-imidazole-substituted precursor resulted in isatoic anhydride (78%). C2-unsubstituted indoles were unreactive under these conditions. The key intermediacy of 2-aryl-3*H*-indol-3-one **(152)** was confirmed by the control experiment, from which **(5)** was formed via Baeyer–Villiger oxidation. Overall, a wide range of benzoxazine-4-ones were obtained under transition-metal-free conditions using inexpensive oxone as a sole oxidant.

**From Functionalized Isatins**

**Scheme 48:**
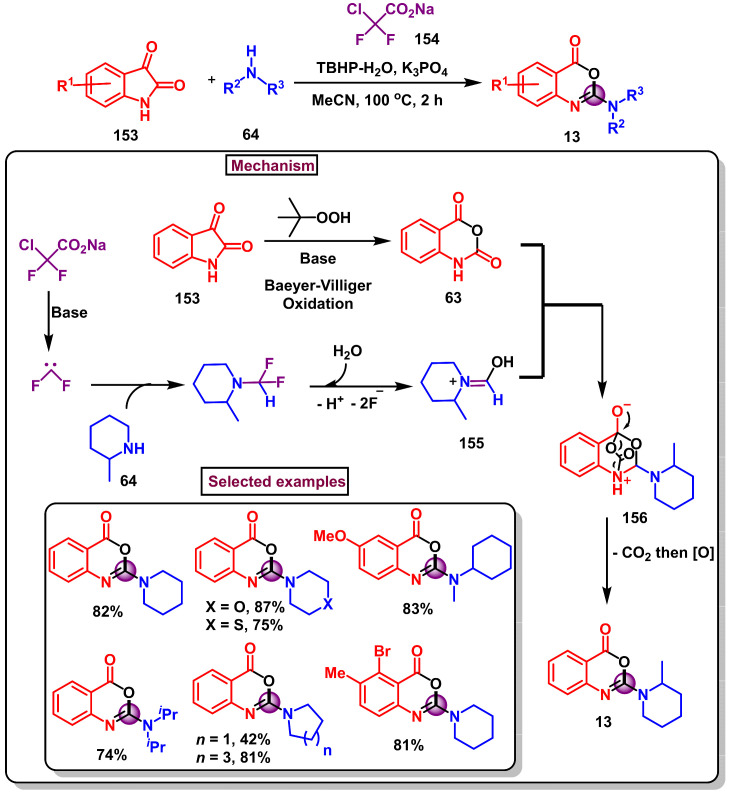
TBHP-promoted synthesis of 2-aminobenzoxazinones from isatins and amines.

A complementary approach towards 2-aminobenzoxazinones **(13)** without toxic azides or expensive Pd-catalysts was developed by Yin and Xu’s group (Scheme 48) [105]. To this end, by employing readily available isatins **(153)**, amines **(64),** and sodium chlorodifluoroacetate **(154)** in the presence of TBHP, a series of 2-aminobenzoxazinones were obtained via a cyclization between isatoic anhydride **(63)** and imine intermediates **(155)**. Notably, C2 of benzoxazinone was installed by a quadruple cleavage of **(154)**.The substrate scope revealed that isatin **(153),** bearing strong electron-withdrawing group (-NO_2_), hampered the reaction and only a complex mixtrure was obtained. On the other hand, the electron-donating groups (-Me, -OMe, OCF_3_) and halogens (Br, Cl, F) favored the reaction and afforded the corresponding benzoxazin-4-ones in good yields (up to 80%). The reaction proceeded well with various cyclic aliphatic amines such as piperidine, thiomorpholine, morpholine, and acyclic secondary amines **(64)**. However, 1,2,3,4-tetrahyrdoquinoline displayed low reactivity due to the π -conjugation between N and the aryl ring. Mechanistically, isatoic anhydride **(63)** (formed via the Baeyer–Villiger oxidation of isatin) underwent intermolecular cyclization with hydroxy imine cation **(155)** to yield **(156),** which after decarboxylation and oxidative dehydrogenation provided the 2-aminobenzoxazinones **(13)**. The synthetic utility was further demonstrated by a gram-scale synthesis (65%)

**Scheme 49:**
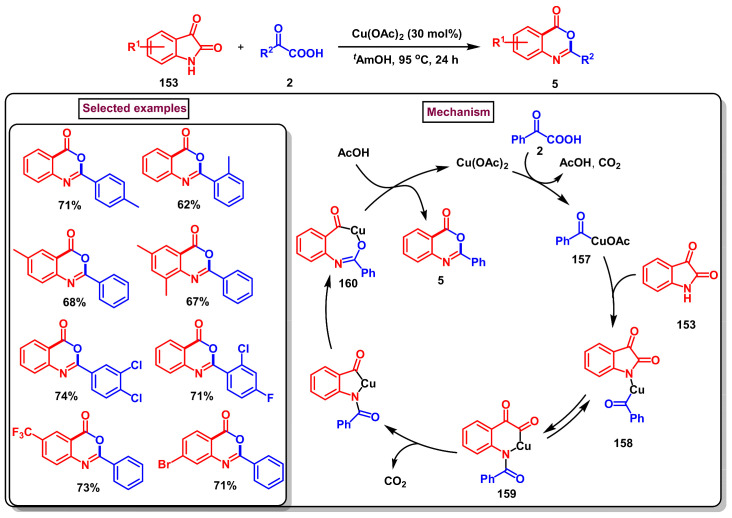
Cu(II)-catalyzed decarboxylative coupling of isatins with arylglyoxylic acids.

Cu(II)-catalyzed reactions of isatins with arylglyoxylic acids via decarboxylative coupling strategy was developed to access wide range of [1,3]-benzoxazine-4-ones **(5)** [106]. Decabonylation of isatin and simultaneous formation of C-N and C-O bonds observed during this transformation. Varieties of α-oxocarboxylic acids **(2)** and isatins **(153)** with range of functional groups were found suitable substrates (Scheme 49). No desired product was obtained with *N*-protected isatins whereas inferior yield was observed in the case of ortho-substituted α-oxocarboxylic acids. Isatins with halogens, CF_3_, Me, OMe provided the desired benzoxazin-4-ones **(5)** in moderate to good yields (up to 82%). Product formation was not affected by the nature and position of the substitutions. However, strongly electron-withdrawing The NO_2_ group led to no desired product. A diverse range of α-aryl and α-heteroaryl oxocarboxylic acids **(2)** were tested. It was observed that sterically demanding ortho-substitutions slightly lowered the product yield. As observed in the scope of isatin, NO_2_-bearing α-phenyl oxocarboxylic acid failed to react under these conditions. A plausible mechanism stated that the Cu-catalyzed decarboxylation of **(2)** led to species **(157)**. Further reaction with isatin generated **(153),** which might be in equilibrium with **(159),** owing to the Cu-migration. Decarbonylation and subsequent rearrangement led to **(160),** from which **(5)** was formed via reductive elimination. 

## 8. Preparation of Benzo[*d*][1,3]-Oxazin-4-Ones via Miscellaneous Procedures

**Scheme 50:**
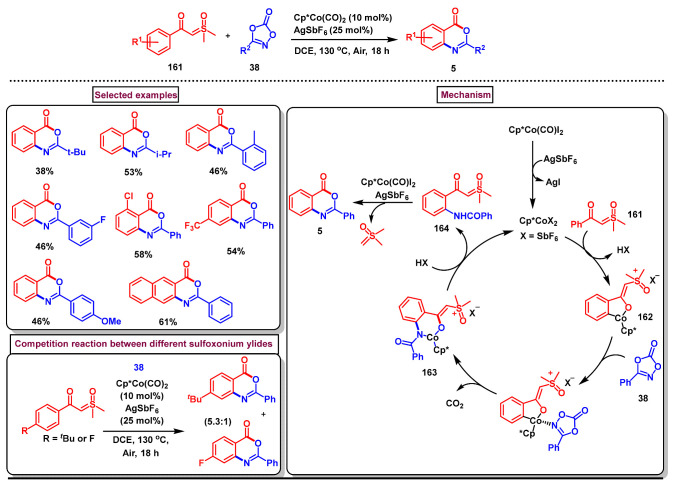
Preparation of benzoxazine-4-ones Via Co(III)-catalyzed C-H activation/[3+3] annulation.

Co(III)-catalyzed C-H activation followed by a [3+3] annulation sequence between sulfoxonium ylides **(161)** and dioxazolones **(38)** was reported under base-free conditions to access 2-substituted benzoxazine-4-ones **(5)** (Scheme 50) [107]. Optimizational studies revealed that the reaction temperature (130 °C) is crucial, as lower temperatures lead to ortho-amidated sulfoxonium ylides **(164)**. A considerable steric influence was exerted by ortho-substitutions which lowered the yields (52–58%). Other common groups such as halogens CF_3_, *t*Bu, and Ph were found compatible under the reaction conditions. However, the heteroaryl-embedded sulfoxonium ylide was not suitable for this transformation. Further evaluation of generality revealed that the dioxazolones with ortho-functionalizations led to the corresponding 2-arylbenzoxazin-4-ones in lower yields owing to the steric impact (46–51%). It is worth mentioning that alkyl and heteroaryl-substituted dioxazolones were also participated in this process and delivered the products in moderate yields (32–53%). Control experiments suggested that AgSbF_6_ and Cp*Co(CO)I_2_ were served as Lewis acids and assisted in the carbonyl activation of **(161)**. The observed low KIE value (K_H_/K_D_ = 1.00) revealed that the C-H activation is not a rate-determining step. However, the author proposed that the intramolecular nucleophilic cyclization of **(164)** determines the rate. Intermolecular competitive reactions proved that sulfoxonium ylides with electron-rich substituent (*t*-Bu) reacted efficiently with **(38)**. Mechanistically, five-member cobaltacycle **(162)** was formed through the interaction of in situ-generated active Co(III) catalyst with carbonyl group of **(164)** followed by C-H activation. The subsequent *N*-coordination of **(38)** to Co center, extrusion of CO_2,_ and migratory insertion led to intermediate **(163)**. Formation of **(164)** via protonolysis and intramolecular cyclization generated the product **(5)**.

**Scheme 51:**
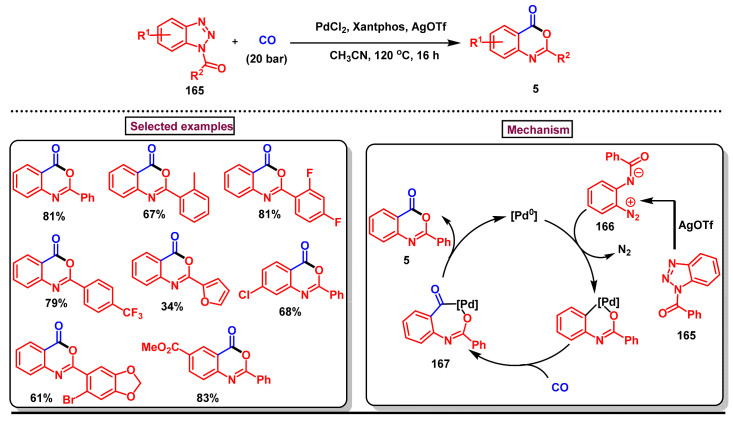
Pd-catalyzed carbonylative transformation of benzotriazoles into benzoxazine-4-ones.

The carbonylative transformation of benzotriazoles **(165)** into benzoxazine-4-ones **(5)** was realized by Wu et al. (2017), employing an Ag/Pd-based bimetallic system and CO (20 bar) [108]. Under neutral conditions, a range of benzoxazine-4-ones were obtained with remarkable functional group compatibility (Scheme 51). Effects of catalysts, co-catalysts, and ligands were studied in detail. A broad range of 2-alkylated, arylated, and heteroarylated benzoxazin-4-ones were obtained from their respective precursors in moderate to good yields. Specifically, synthetically useful amines, ketones, cyano, and CF_3_ functionalities could be incorporated into the 2-aryl group. On the other hand, variations in the aryl ring of 1,2,3-benzotriazoles showed that, in few cases, inseparable mixtures of regioisomers were formed (2.2:1-3:1). A plausible mechanism for this carbonylative transformation under the Pd/Ag bimetallic catalytic system was proposed. Accordingly, the Ag-catalyzed ring opening of **(165)** generated the aryldiazonium species **(166)**. Further oxidative addition of Pd(0) with **(166)** and CO insertion led to seven-member intermediate **(167)**. Desired product **(5)** was obtained via reductive elimination. A similar Pd-Ag-bimetallic catalytic system based on denitrogenative carbonylation using Cr(CO)_6_ as a carbonyl surrogate to prepare benzoxazin-4-ones was also reported by the authors [109].

**Scheme 52:**
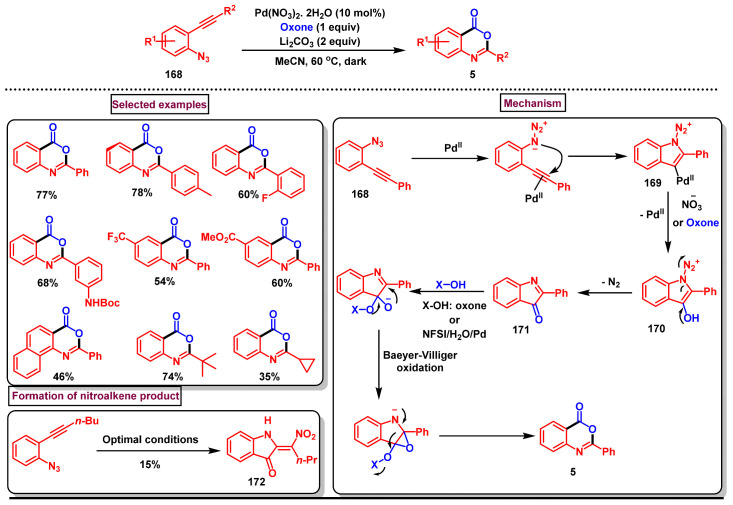
Preparation of benzoxazine-4-ones via Pd-catalyzed alkyne cleavage of 2-azidoalkynylbenzenes.

Cleavage of alkynes under transition-metal-catalyzed conditions is a challenging task due to the large bond dissociation energy (≈ 200 kcal/mol). Liu et al. reported a Pd-catalyzed approach to prepare benzoxazin-4-ones **(5)** via alkyne cleavage of 2-azidoalkynylbenzenes **(168)** in the presence of an oxidant (NFSI or oxone) (Scheme 52) [110]. This process involved a sequential aminopalladation/C-Pd oxidation/oxidative rearrangement of azidoalkynes **(168)**. Variously substituted aryl alkynes, cyclic and branched aliphatic alkynes yielded the desired benzoxazine-4-ones (35–78%). The aryl group of alkynes displayed equal outcomes for both electron-withdrawing and donating groups. However, when R^2^ is a linear alkyl group, an alkene product **(172)** is obtained. On the other hand, useful functional groups such as ester, ether, nitro, and halogens were smoothly incorporated into the aryl skeleton of 2-azidoalkynylbenzenes **(168)**. Intermediacy of 2-phenyl-3*H*-indol-3-one **(171)** was confirmed by isolation and further transformed into product **(5)** using the oxidant (NFSI of oxone). The proposed pathway revealed that the initial formation of the indolyl-Pd complex **(169)** was realized via aminopalladation, followed by an oxidation of C-Pd bond, generating 3-hydroxyindole **(170)**. Further tautomerization to ketone **(171)** and Baeyer–Villiger oxidation led to desired product **(5)**.

**Scheme 53:**
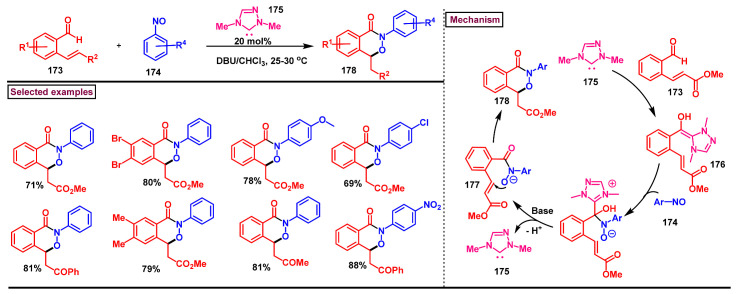
NHC-catalyzed Synthesis of 2,3-benzoxazin-4-ones from ortho-formyl cinnamates and nitroso arenes.

Another special example of preparation of the multi-substituted 2,3-benzoxazin-4-ones **(178)** was achieved under a NHC (carbene)-catalyzed reaction of ortho formyl cinnamates/chalcones **(173)** and nitrosoarenes **(174)** (Scheme 53) [111]. This transformation featured a cascade aza-benzoin reaction/oxo-Michael intramolecular addition sequence. Strong electron-rich substituents (OMe) and electron-withdrawing substituents (NO_2_) on nitrosoarenes **(174)** were found inert under the reaction conditions. In contrast, 2-formylcinnamate with electron-donating -OMe group displayed inertness. However, -Br, -NO_2_ groups increased the efficiency to afford the desired product in good yields (77–80%). The scope of this NHC-catalyzed cascade annulation was further expanded to various o-formyl chalcone derivatives, and the products were isolated in good yields (77–92%). This process was proceeded via the formation of Breslow intermediate **(176)** from the addition of NHC catalyst **(175)** to the aldehyde of **(173)**. Reaction of enol **(176)** with nitroso precursor **(174)** followed by elimination of **(175)** resulted in the anion **(177)**. The desired product **(178)** was formed via the intramolecular oxo-Michael addition. The synthetically useful 2-oxo-1-isobenzofuranacetates and β-hydroxycarboxylates under reductive conditions were prepared to showcase the synthetic potential.

**Scheme 54:**
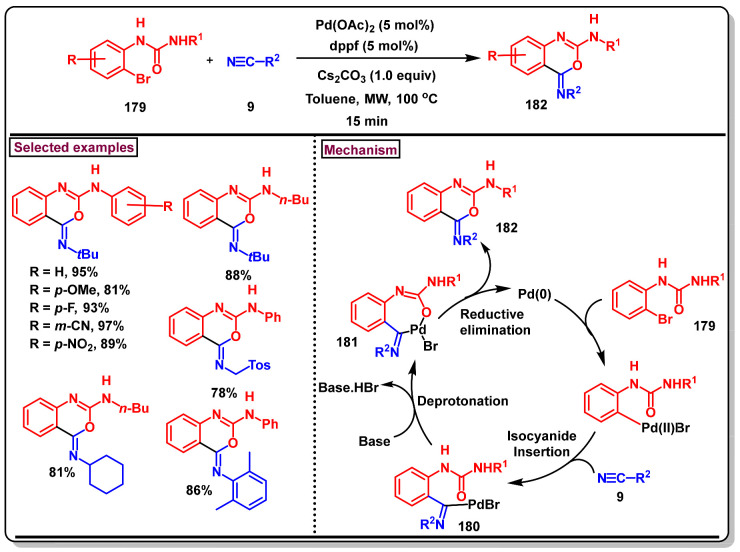
Microwave assisted Pd-catalyzed isonitrile insertion in 2-bromophenylureas.

Batra et al. envisioned a Pd-catalyzed insertion of isocyanides **(9)** into 2-bromophenyl ureas **(179)** to access 4-functionalized imino-4*H*-benzo[*d*][1,3]oxazin-2-amines **(182)** in an one-pot manner (Scheme 54) [112]. This microwave-assisted isocyanide insertion of **(179)** was followed by a C-O coupling of imidoyl-Pd intermedite to afford the desired products. Diverse range of aryl, benzyl and alkyl amino units were installed at C2 position from the suitably substituted **(179)**. Both electron-withdrawing (-NO_2_, -CN, halogens) and electron-donating groups (-OMe) were found compatible and the corresponding 4-(*tert*-butylimino)-*N*-substituted alkyl or aryl-4*H*-benzo[*d*][1,3]oxazin-2-amines were isolated in good yields (up to 97%). Arylisocyanide, tosylmethylisocyanide and cyclohexyl isocyanides were smoothly involved in this transformation. Imidoyl intermediate **(180)** was formed via an oxidative addition of Pd(0) to substrate **(179)** followed by an isocyanide insertion. Base-mediated deprotonation yielded the seven-member transtion state **(181)**. Finally, 4-imino-4*H*-benzo[*d*][1,3]oxazin-2-amine **(182)** was generated via reductive elimination and Pd(0) was regenerated. Conversion of **(182)** into *N*-substituted quinazolinones and hydrolysis of imine into carbonyl groups were performed as part of post synthetic applications.

**Scheme 55:**
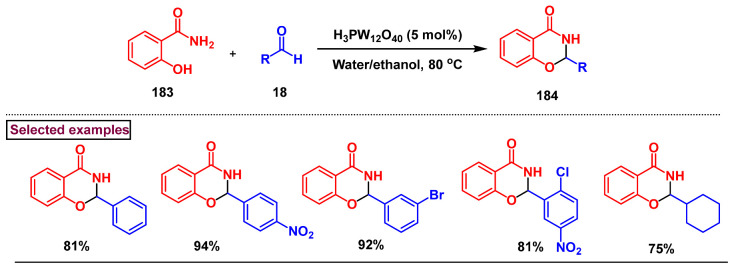
Heteropoly acid-catalyzed cyclocondensation of salicylamides and aldehydes.

An efficient cyclo-condensation of commercially available salicylamide (**183**) and aldehydes (**18**) was realized in the presence of H_3_PW_12_O_40_ to afford a series of 1,3-benzoxazin-4-ones (**184**) in good to excellent yields (up to 94%) (Scheme 55) [113]. Importantly this reaction was carried out under mild and aqueous conditions. The heteropoly acid used for the reaction could be reused up to three times without appreciable loss in reactivity. Electron-withdrawing (-NO_2_, -CN) groups, halogens, naphthyl groups on the aldehyde precursors (**18**) were compatible and led to the desired compounds in higher yields. Cyclohexaldehyde led to moderate yield (75%) whereas formamide resulted in the formation of bisamide (71%). A regular acid-catalyzed mechanism was discussed for this transformation. Protonation of carbonyl group of aldehydes by H_3_PW_12_O_40_ was followed by the nucleophilic attack of hydroxy group of salicylamide (**183**). Cyclization was induced by the amide group with simultaneous elimination of H_2_O provided the 1,3-benzoxazin-4-one (**184**). A parallel synthesis of quinazolinones was achieved from 2-aminobenzamides and aldehydes under similar conditions.

## 9. Preparation of Fused Benzo[*d*][1,3]-Oxazin-4-One Derivatives

**Scheme 56:**
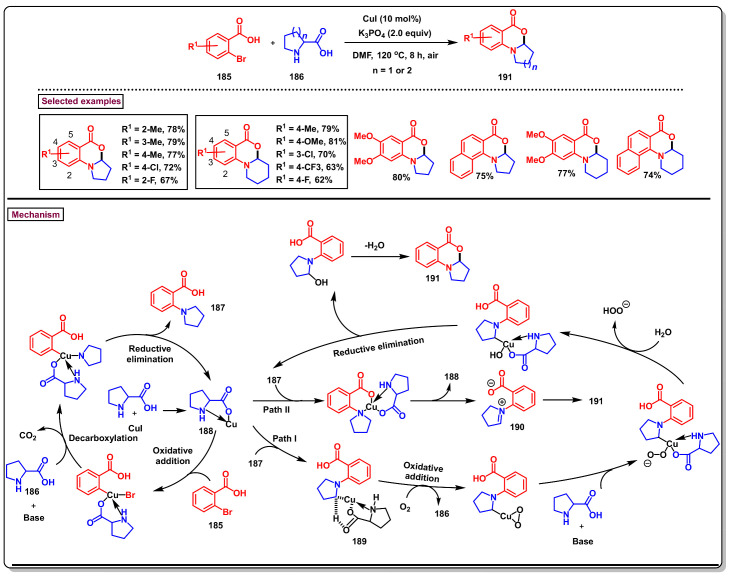
Cu-catalyzed synthesis of pyrrolo/pyrido benzoxazinones via Ullmann coupling/dehydration/decarboxylation.

Recently, Cu-catalyzed regioselective approach was developed to access fused pyrrolo/pyrido benzoxazinone structures **(191)** from easily available 2-bromobenzoic acids **(185)** and proline or pipecolic acids **(186)** via sequential Ullmann coupling/dehydration/decarboxylation. (Scheme 56) [114]. Both prolines and pipecolic acid derivatives were smoothly reacted and afforded the corresponding products in moderate yields (60–81%). However, heteroaryl counterparts such as 2-bromo-3-thiophenecarboxylic acid, 3-bromo-2-pyridine carboxylic acid and 3-bromo-2-thiophenecarboxylic acid were failed to produce the desired products under these conditions. Interestingly, when chiral prolines (D or L) and L-pipecolic acids were employed, no optical rotation was observed on the desired products suggesting the decarboxylation process. Mechanistically, key intermediate **(187)** was formed via Cu-catalyzed Ullmann coupling and decarboxylation process. Two possible coordination ways are proposed for this transformation. In pathway 1, Cu-complex **(188)** coordinated to **(187)** via several possible H-bonding and led to **(189)**. Sequential oxidative addition, reductive elimination and dehydration afforded the desired product **(191)**. Alternatively, in pathway 2, *N*-atom of key intermediate **(187)** coordinated with Cu-of **(188)** and provided the intermediate **(190)**. Further reductive elimination and cyclization offered the product **(191)**. Overall, a highly valuable benzoxazine-4-one derivatives were achieved from readily available substrates with excellent substrate scope.

**Scheme 57:**
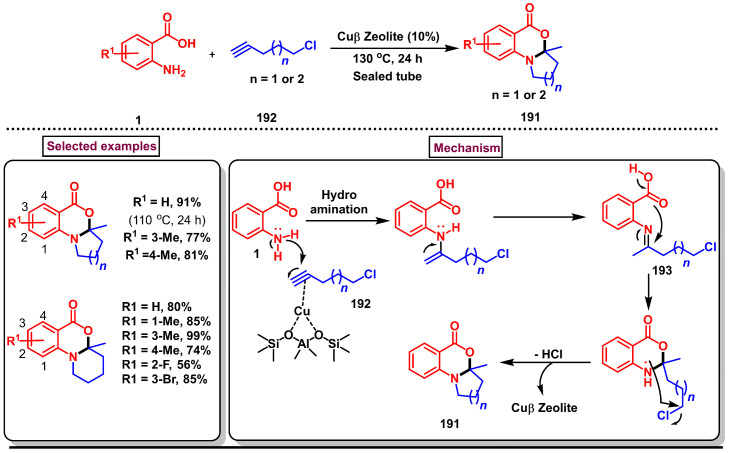
Cuβ zeolite-catalyzed one-pot synthesis of pyrrolo/pyrido benzoxazinones.

Metal-modified zeolite heterogeneous catalysts are frequently employed in various organic transformations owing to their high surface area, thermal stability, uniform channel size and environmentally friendly properties [115]. To this end, a one-step synthesis of preparation of fused pyrrolo/pyrido benzoxazinones **(191)** was realized from anthranilic acids **(1)** and chloro alkynes **(192)** by using Cuβ zeolite (Scheme 57) [116]. This transformation features Markownikoff’s hydroamination of alkynes and intramolecular cyclization. Anthranilic acids **(1)** with activating groups (-Me) and halogens (-Br, -F) exhibited excellent reactivity towards 6-chloro-1-hexyne and 5-chloro-1-pentyne. Corresponding pyrrolo- and pyridobenzoxazin-4-ones **(191)** were obtained in good yields (56–91%). Adsorption of terminal alkyne **(192)** into the acid sites of Cuβ zeolite and hydroamination yielded the imine **(193)**. Subsequent intramolecular cyclization and dehydrochlorination offered the desired fused benzoxazinones **(191)**. Cuβ zeolite used in this process was effectively reused up to five times without appreciable decrease in the yields. In addition, fused quinazolinones and thiadazine compounds were also obtained by employing anthranilamides and 2-aminobenzenesulfonamides with terminal alkynes. Furthermore, in vitro anticancer, antimicrobial and antifungal activities of these compounds were evaluated. 

**Scheme 58:**
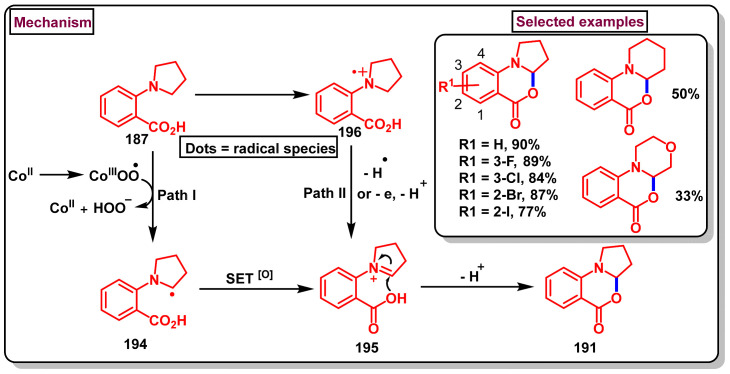
Co-catalyzed intramolecular oxidative cross coupling approach.

Liu and Shang et al. combinedly reported a Co-catalyzed formation of C-O bond between carboxylic acid and (sp^3^) C-H center via aerobic, intramolecular oxidative cross coupling strategy (Scheme 58) [117]. This method enabled the formation of diverse range of pyrrolidine, piperidine, and morpholine fused dihydro-benzoxazinones **(191)** in good yields. No significant difference was observed with 2-(pyrrolidine-1-yl) benzoic acids bearing electron-rich and poor substituents. Worth noting, a formyl group and halogens (-Br, -Cl, -I) remained intact to yield the corresponding fused dihydro-benzoxazinones in good yields (77–90%). Additionally, 2-morpholinobenzoic acid and 2-(piperidin-1-yl) benzoic acid were also reacted albeit in lower yields (33% and 50%, respectively). However, 2-(indolin-1-yl) benzoic acid did not react under the optimum conditions. Compared to the toxic HgO-mediated literature methods [118], this process proceeded at ambient temperature by utilizing molecular O_2_ as an oxidant and H_2_O was the sole byproduct. H-abstraction by Co(III) peroxy radical generated the radical intermediate **(194)** which further oxidized to imine **(195)**. Alternatively, **(196)** was proposed to form through SET oxidation of **(187)** and elimination of radical H. Further intramolecular cyclization and deprotonation afforded the desired product **(191)**. The Authors proposed that catalytic tyrosine is required to stabilize Co-species during the catalytic process.

**Scheme 59:**
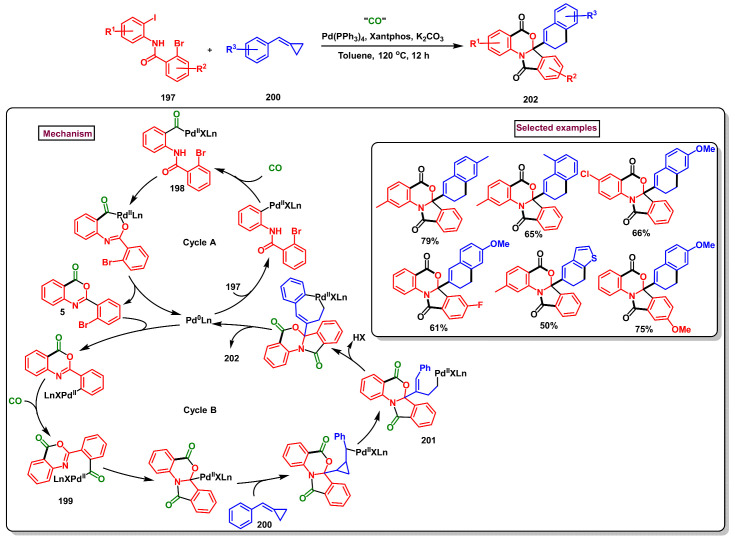
Pd-catalyzed cascade carbonylation strategy for benzoxazinone-fused isoindolinones.

Recently, Pd-catalyzed cascade carbonylation process was developed to access a wide range of benzoxazinone-fused isoindolinones **(202)** from 2-bromo-*N*-(2-iodophenyl)benzamides **(197)** and benzylidenecyclopropanes **(200)** (BCPs) (Scheme 59) [119]. It should be noted that installation of two carbonyl groups, formation of four carbon-carbon bonds and two carbon-heteroatom bonds were realized in one step. A series of BCPs bearing functional groups such as ether, thioether, ester, halogens, were found compatible. In general, electron-donating groups led to higher yields than electron-withdrawing groups. Sterically bulkier ortho-substitutions also hampered the reaction and lowered the yields. Additionally, thiophenyl and naphthyl tethered BCPs were also smoothly reacted to give the target products in moderate yields (50–52%). On the other hand, irrespective of the electronic nature various *N*-bromobenzoyl-*o*-iodoanilines were involved in this transformation and the corresponding tetracyclic products were obtained in moderate to good yields (59–79%). Proposed catalytic cycle A led to intermediate benzo[*d*]oxazin-4-one **(5)** via the oxidative formation of aryl-Pd complex, CO insertion **(198)**, intramolecular cyclization and reductive elimination. Regenerated Pd(0) catalyst initiated another set (cycle B) of oxidative addition, CO insertion and led to acyl-Pd complex **(199)**. Further intramolecular cyclization, migratory insertion of **(200)**, β-carbon elimination generated the complex **(201)**. Base-promoted internal aromatic electrophilic substitution, and reductive elimination offered the desired product **(202)**.

**Scheme 60:**
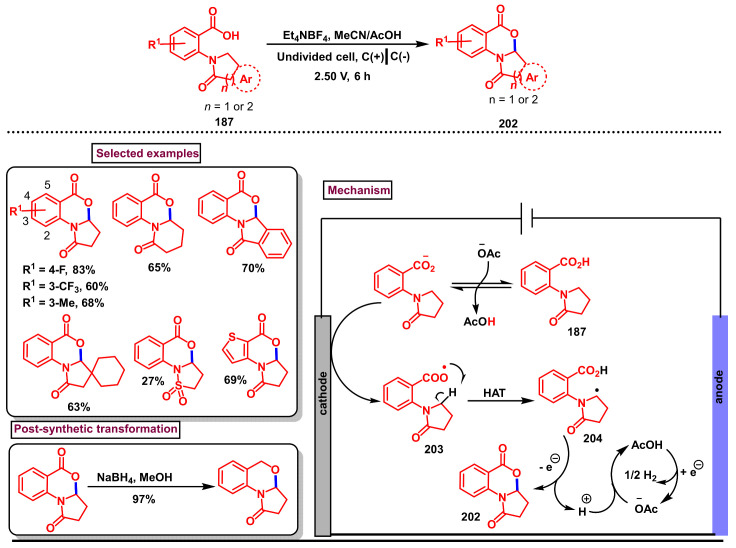
Electrochemical oxidative lactonization approach to prepare lactone-fused benzoxazinones.

An unusual Shono-type oxidation of functionalized anthranilic acids **(187)** via C(*sp^3^*)-H activation was developed to access various lactone-fused benzoxazinones **(202)** under electrochemical conditions (Scheme 60) [120]. This atom-economic, electrochemical oxidative lactonization was proceeded under mild conditions by using affordable graphite electrodes and Et_4_NBF_4_ electrolyte with an operating potential of 2.5 V. Biologically important spiro lactams and cyclic sulphamides were also obtained in moderate yields. Benzoic acid derivatives bearing aryl, alkyl, alkoxyl, and halogens were found viable, and the corresponding fused products were obtained in good to excellent yields (43–97%). Notably, heteroaryl incorporated substrate was also amenable and offered the desired tricyclic product in 69% yield. The scope was further expanded to six-member lactam, benzo lactam and spiro variants (46–70%). Interestingly, cyclic sulphamide also worked well albeit in low yield (27%). Control experiments supported the radical process and ruled out the involvement of typical iminium intermediate **(195)**. On the other hand, cyclic voltammetry studies revealed that an oxidative peak (1.38 V) owing to the deprotonation of **(187)** by OAc^-^ was observed at cathodic center. Further oxidation into a radical **(204)**, intramolecular hydrogen atom transfer (HAT) process and radical cyclization led to corresponding product **(202)**. A selective reduction in ester carbonyl was demonstrated to prove the chemical applicability. The scalability of this method was demonstrated by a gram-scale synthesis.

**Scheme 61:**
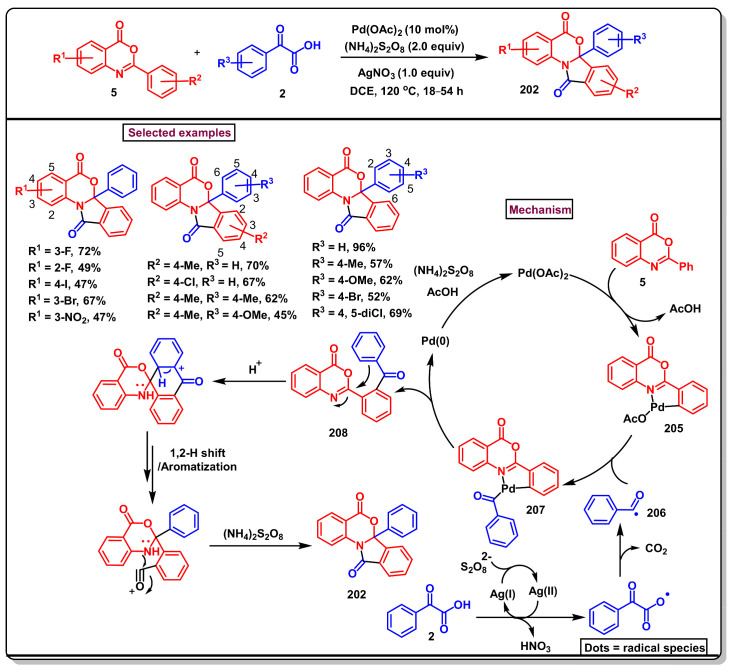
Pd-catalyzed decarboxylative acylation 2-aryl benzoxazine-4-ones with α-oxo carboxylic acids.

Direct synthesis of functionalized isoindolinones **(202)** was achieved via Pd-catalyzed decarboxylative acylation strategy from 2-aryl benzoxazine-4-ones **(5)** and α-oxo carboxylic acids **(2)** with the aid of oxidant (NH_4_)_2_S_2_O_8_ and AgNO_3_ as a co-oxidant (Scheme 61) [121]. Control experiments revealed that this radical process occurred through the formation of isolable, acylated 2-aryl benzoxazine-4-one intermediate **(208)** which underwent cyclization in the absence of Pd-catalyst. Moreover, (NH_4_)_2_S_2_O_8_ led to the desired product even in the absence of co-oxidant. Strong electron-withdrawing -NO_2_ group on the aryl ring of **(5)** lowered the yield (44–47%). However, halogens, -OMe, -Me groups resulted in comparatively higher yields (57–62%). Moreover, α-oxo carboxylic acids **(2)** also demonstrated a fair scope. It should be noted that in a few cases (-Me, -OMe) relatively longer reaction time was required. Initially formed five-member Pd-complex **(205)** reacted with acyl radical **(206)** which in turn formed from α-oxo carboxylic acid **(2)** via single electron abstraction followed by decarboxylation. Acyl Pd-complex **(207)** provided **(203)** via reductive elimination. A series of intramolecular cyclizations afforded the isoindolinone **(202)**. In another report in 2018, authors demonstrated a similar radical process to prepare isoindolinones from 2-aryl benzoxazine-4-ones and toluene, aldehydes or benzyl alcohols [122]. Saha et al. (2022) prepared a similar fused isoindolinones under heterogeneous magnetic Pd-catalyzed conditions [123]. This transformation was proceeded under relatively mild conditions using aqueous solvent medium.

**Scheme 62:**
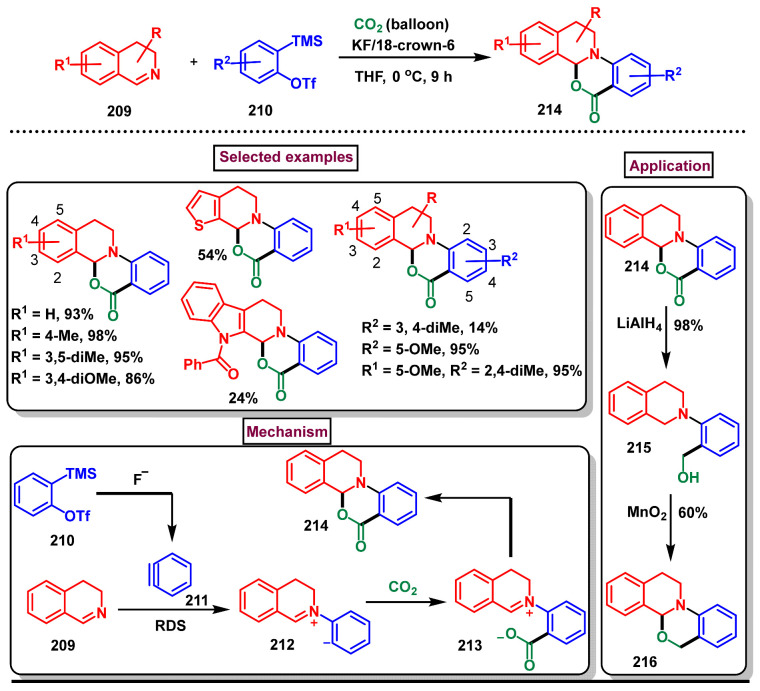
Preparation of tetrahydroisoquinoline-fused benzoxazinones via [2+2+2] annulation strategy.

A convergent [2+2+2] annulation strategy was developed from benzynes, CO_2_ and 3,4-dihydroisoquinolines **(209)** to access tetrahydroisoquinoline-fused benzoxazinones **(214)** by using non-toxic CO_2_ as a C1 source and KF/18-crown-6 as the fluoride source (Scheme 62) [124]. It was observed that electron deficient benzyne precursors led to higher yields by favoring the intermolecular nucleophilic attack of cyclic imines **(209)**. Further derivatization of **(214)** into alcohol **(215)**, and ether **(216)** proven the synthetic utility. Introduction of electron-withdrawing groups such as -Me, -OMe drastically increased the yield up to 98%. In the case of halogenated substrates, moderate yields of the desired products were obtained (50–59%). Heterocyclic and naphthyl fused 3,4-dihydroisoquinolines were found less reactive and the corresponding products were obtained in lower yields (24%). The steric effect of substituents was also crucial in this process. As expected, the reactivity of electrophilic partner (benzyne) was largely influenced by the electronic environment. Electron-withdrawing -F group led to higher yield (97%) , whereas -Me group lowered the yield to 14%. A successful gram-scale experiment further demonstrated the practicality of this method. Proposed mechanism stated that zwitter-ionic intermediate **(212)** was formed via the nucleophilic attack of **(209)** to in situ-generated **(211)** which rapidly reacted with CO_2_ and provided the carboxylate **(213)**. Further ring closure led to **(214)**. Two examples were discussed in an iminium-ion-mediated double annulation cascade strategy reported by Sharada et al. (2016) [125], whereas one example of tetrahydroisoquinoline-fused benzoxazinones was displayed in a photocatalyst enabled oxidative C-H functionalization developed by Che et al. (2013) [126].

**Scheme 63:**
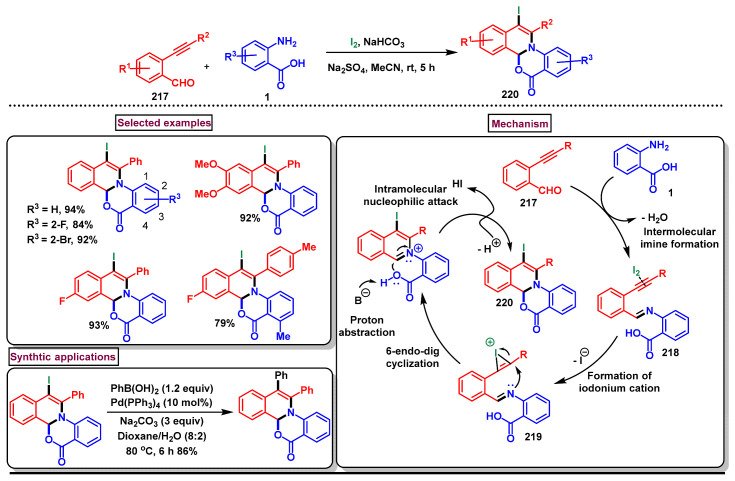
I_2_/base-mediated electrophilic iodocyclization 2-alkynylarylaldehydes with anthranilic acids.

I_2_/base-mediated tandem electrophilic iodocyclization of functionalized 2-alkynylarylaldehydes **(217)** with anthranilic acids **(1)** was demonstrated to yield iodinated 1,2-dihydroisoquinoline-fused benzoxazinones **(220)** at room temperature (Batra et al. 2013) [127]. A wide substrate scope and excellent yields were demonstrated, and the consecutive formation of N-C/C-N/O-C/C-I bonds was realized during this transformation (Scheme 63). It should be noted that anthanilic acids with electron-withdrawing -NO_2_ substituent failed to generate the aldimine. In the case of anthranilic acids **(1)** bearing the -NO_2_ group, the reaction was deterred, and no desired product was isolated. Other substituents such as halogens, -Me, and -OMe were found reactive and afforded the corresponding tetracyclic products in excellent yields (up to 92%). In contrast, the alkyne precursor **(217)** was insensitive to the nature of substitutions. In most of the cases studied, the desired products were obtained in good yields (up to 92%). The mechanistic proposal revealed that the initially formed aldimine **(218)** generated iodonium complex **(219)**. Sequential intramolecular 6-*endo*-*dig* cyclization, base-promoted cyclization, and deprotonation provided the desired tetracyclic products **(220)**. Anhydrous Na_2_SO_4_ favored the reaction by absorbing the water eliminated during the condensation step. The usefulness was further demonstrated by achieving the synthesis of isoquinoline-fused quinazolinones by employing 2-aminobenzamides. Under Pd-catalyzed conditions, the iodo group on the product was derivatized via the Suzuki and Sonagashira cross-coupling.

**Scheme 64:**
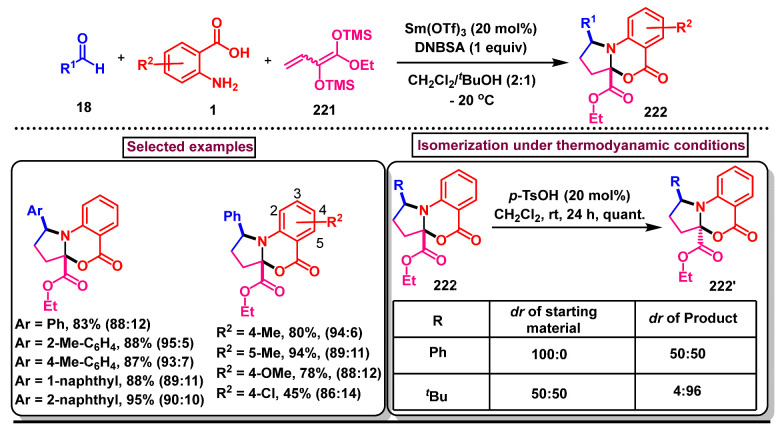
Lewis acid-catalyzed annulation of 2-aminobenzoic acid-derived imines to access pyrrolobenzoxazin-4-ones.

In continuation of the preparation of tetrahydropyrrolo [2,1-*b*]benzoxazoles [128,129], Schneider’s group reported a Lewis acid-catalyzed cyclo-annulation of 2-aminobenzoic acid-derived imines with bis-silyldienediolate **(221)** to access pyrrolobenzoxazinones **(222)** in a stereocontrolled fashion via a vinylogous Mannich reaction of **(221)** and intramolecular N-O acetal formation (Scheme 64) [130]. This reaction favored the *cis*-isomer with a ratio of 85:15 in the presence of the Sm(OTf)_3_/DNBSA system. This heteroannulation process was smoothly proceeded with various aryl, naphthyl, and alkyl aldehydes **(18)**. Specifically, the presence of electron-donating groups (-Me, -OMe) and halogens (-Br, -Cl) favored the *cis*-isomer (dr = 88:1–95:5). However, aryl aldehydes with electron-withdrawing groups (-CN), heteroaryl aldehydes, and bulkier aliphatic aldehydes resulted in poor selectivity (50:50). Interestingly, the exclusive formation of *trans* isomer (3:97) was observed when the reaction was performed at room temperature with pivalic aldehyde. Structural variations on anthranilic acid **(1)** revealed that electron-donating (-Me, -OMe) groups and halogens produced the desired fused products in good yields (45–99%) with acceptable diastereoselectivity (dr = 86:14–94:6). It is proposed that the observed cis-selectivity was owing to the non-bonding interactions between the substituents on the pyrrolidine unit and the anthranilic acid during the intramolecular cyclization stage. However, this ratio could be altered towards the thermodynamically stable isomer in the presence of acid catalyst **(222′)**. The thermodynamic ratio largely depends on the size of pyrrolidine substituents. Additionally, a one-pot synthesis of pyrroloquinazolinones was also demonstrated from anthranilic amide with an excellent diastereomeric ratio (98:2).

## 10. Preparation of Benzo[*e*][1,3]-Oxazin-4-One Derivatives

**Figure 4:**
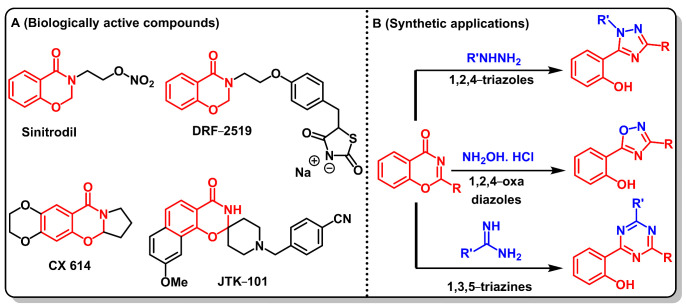
Significance of benzo [1,3][*e*]oxazin-4-one derivatives. (**A**). Biologically active compounds with benzo [1,3][*e*]oxazin-4-one skeleton; (**B**). Syntshetic applications of benzo [1,3][*e*]oxazin-4-ones.

Benzo [1,3][*e*]oxazin-4-ones are important structural congener found in various biologically important skeletons (Figure 4A) [131,132,133,134]. In addition to the widely accepted pharmacological skeletons, this ubiquitous core is often utilized as a key intermediate in the preparation of 1,2,4-oxadiazoles, 1,3,5-triazines and functionalized triazoles (Figure 4B) [135,136,137]. Owing to their significance, several synthetic approaches are constantly designed for their selective synthesis. Herein, we discuss the recent synthetic developments towards these attractive target compounds.

**Scheme 65:**
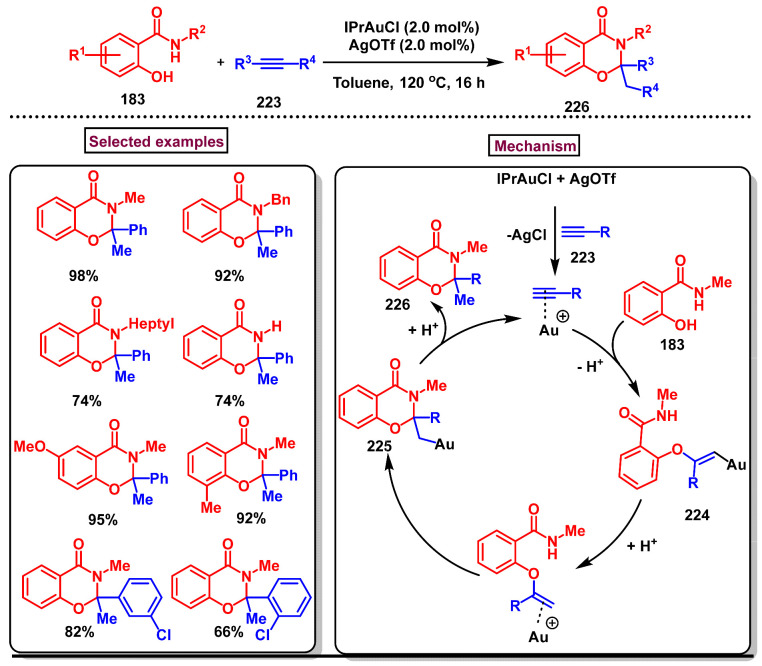
Gold(I)-catalyzed construction of 2,3-dihydro-4*H*-benzo[*e*][1,3]oxazin-4-one via heteroannulation.

Gold-catalyzed annulations involving reactive alkynes such as α,β-unsaturated alkynones, ynamides are highly desirable for the construction of *N*-heterocycles owing to the ease of activation of alkynes. However, the use of non-polar alkynes is challenging due to their poor reactivity. To this end, Gold(I)-catalyzed construction of 2,3-dihydro-4*H*-benzo[*e*][1,3]oxazin-4-one **(226)** via heteroannulation of alkynes **(223)** with salicylic amides **(183)** under mild conditions was combinedly reported by Abe and Inamoto et al. (Scheme 65) [138]. Terminal aryl, alkyl, and symmetrical internal alkynes were compatible during this transformation. Alkyl and benzyl groups on the *N* atom of salicylic amide **(183)** resulted in good yields (up to 98%), whereas N-free substrate delivered the product in moderate yield (58%). In contrast, *N*-Ph salicylic amide was not reactive at all. Presence of halogens, -CF_3_, -OMe groups favored the product formation in good yields (66–95%), however electron-withdrawing -NO_2_ group lowered the yield (33%) even with higher catalyst loading. Unsymmetrical aryl alkynes (internal) and aliphatic terminal alkynes **(223)** were compatible to afford the desired products in good yields (66–98%). In the case of bulkier *t*Bu group and ortho-halogenated aryl group, the yield was lowered (20–66%). Albeit with a higher catalyst loading, the symmetrical alkyl and aryl alkynes exhibited moderate reactivity (15–54%). As discussed in Scheme 9, the mechanistic proposal suggested that initial activation of alkyne **(223)** by in situ-generated cationic gold catalyst was followed by the phenolic attack of **(223)** to generate the alkenyl Au-species **(224)**. Further protonation and intramolecular cyclization provided the gold complex **(225)**. The desired product was formed via protonation of **(225)**. The Authors further demonstrated a 1 mmol scale reaction successfully.

**Scheme 66:**
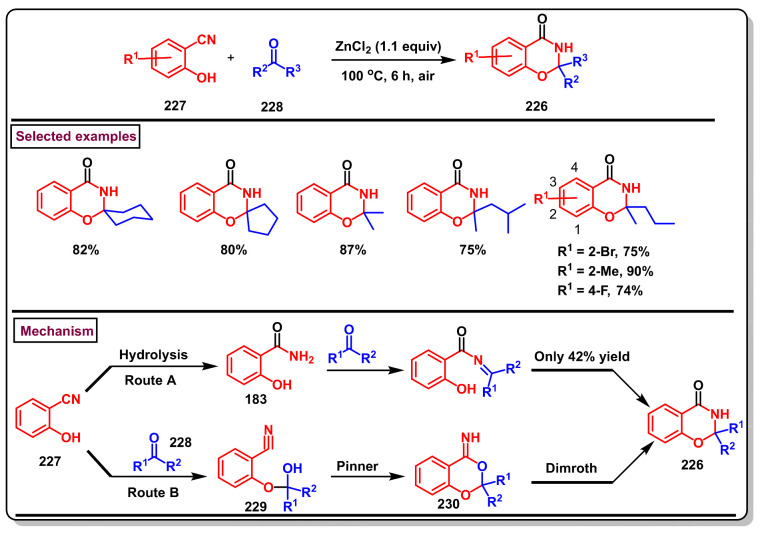
ZnCl_2_-mediated intramolecular tandem Pinner-Dimroth rearrangement.

A facile preparation of a wide range of functionalized of 2,3-dihydro-4*H*-benzo[*e*][1,3]oxazin-4-ones **(226)** via a ZnCl_2_-mediated domino reaction of a ketone **(228)** and 2-hydroxybenzonitriles **(227)** was reported by Li et al. (Scheme 66) [139]. It was proposed that the reaction proceeded through intramolecular tandem Pinner-Dimroth rearrangment. This domino cyclization was effective with various aliphatic, aromatic, cyclic, and branched ketones leading to the desired products in good yields (up to 90%). This transformation was smoothly proceeded with various cyclic, acyclic, (symmetrical and unsymmetrical), and aryl ketones **(228)** to afford the desired spirocyclic products in excellent yields (73–90%). On the other hand, salicylonitriles **(227)** with halogens (-Br, -F), -Me group were tested and the corresponding benzo[*e*][1,3]oxazin-4(3*H*)-ones were obtained in good yields (72–90%). To obtain mechanistic insights, this cyclocondensation was performed from salicylamide **(183)** and the product was obtained in lower yields (42%). Alternatively, under anhydrous conditions **(229)** was formed via an intermolecular addition **(227)** to ketone **(228)**. Formation of **(230)** via intramolecular Pinner reaction followed by Dimroth rearrangement yielded the product **(226)**.

**Scheme 67:**
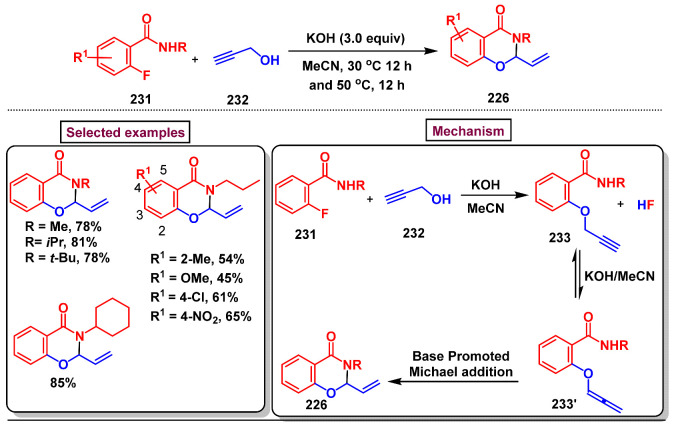
KOH-promoted intermolecular S_N_Ar/cyclization of 2-propyn-1-ol and ortho fluorobenzamides.

A chemodivergent synthesis of 2,3-dihydro-4*H*-benzo[*e*][1,3]oxazin-4-ones **(226)** was realized via KOH-promoted intermolecular S_N_Ar/cyclization of 2-propyn-1-ol **(227)** and ortho-fluorobenzamides **(231)** (Scheme 67) [140]. *N*-alkyl-2-fluorobenzamides bearing 1^o^, 2^o^, 3^o^, cyclic alkyl groups at *N*-center were transformed into 1,3-benzoxazin-4-(4*H*)-ones in good yields (up to 85%). Notably, electron-withdrawing groups such as -NO_2_, -CF_3_ displayed higher reactivity than electron-donating groups (-Me, -OMe). 1-alkyl or 1-phenyl-substituted propargyl alcohols were sluggish and trace product formation was observed. Mechanistically, key intermediate **(233)** was formed via an intermolecular nucleophilic substitution and subsequently isomerized into allenyl species **(233′)**. Further intramolecular aza-Michael-addition delivered the product **(226)**. Based on real-time NMR studies, authors explained that the formation of **(226)** was a slow process due to the poor solubility of KOH in acetonitrile, whereas high solubility in DMSO led to quick formation of 1,4-benzoxazepine-5(4*H*)-ones.

**Scheme 68:**
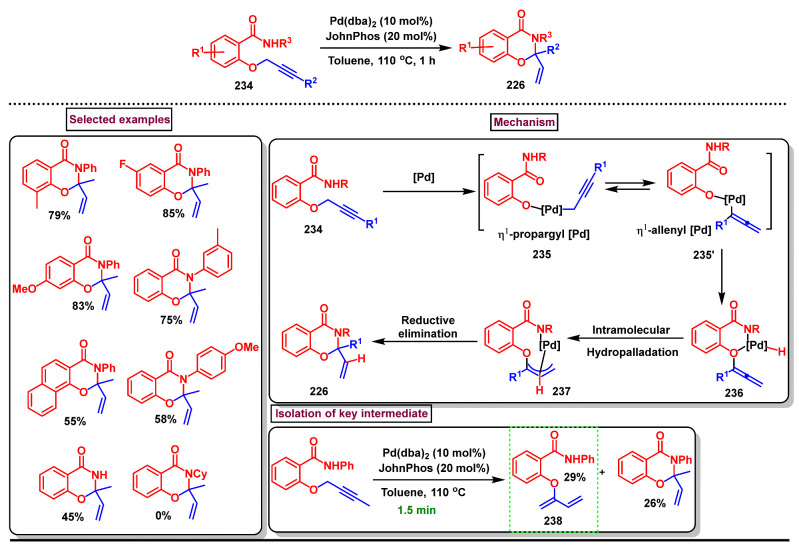
Pd-catalyzed cyclization of alkyne tethered benzamide derivatives.

In 2018, Sakai and Ogiwara et al. described a Pd-catalyzed cyclization of alkyne tethered benzamide derivatives **(234)** to produce 2,3-dihydro-4*H*-benzo[*e*][1,3]oxazin-4-ones **(226)** containing fully substituted allylic carbon (Scheme 68) [141]. This process proceeded under base or oxidant free conditions to deliver the corresponding products in moderate to good yields (up to 97%). Diverse range of salicylamide derivatives possessing halogens, acetyl, -CF_3_ groups were found compatible under these conditions and the corresponding products were isolated in good yields (up to 97%). Secondary aryl amides were efficient whereas primary amide with free -NH was sluggish and poor yield was obtained (20%). On the other hand, 1,3-dienes were formed in the case of *N*-alkyl protected salicylamides. It should be noted that terminal alkynes yielded the 7-*exo*-*dig* adduct. Possible pathway explained that oxidative addition of Pd(0) to the C-O bond of alkyne precursor **(234)** resulted in η^1^-propargyl Pd-complex **(235)** which would be in equilibrium with η^1^-allenyl Pd-complex **(235′)**. Further formation of O-allenyl-Pd complex and intramolecular hydropalladation generated the complex **(237)**. Reductive elimination of allylic C-N bond generated the product **(226)**. With shortened reaction time key intermediate 1,3-diene **(238)** was isolated and converted into product **(226)** with 81% GC yield.

**Scheme 69:**
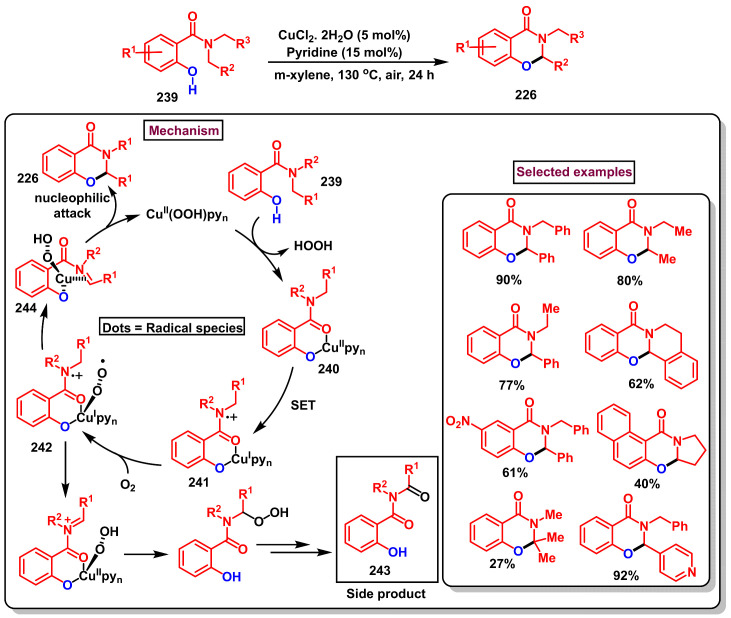
Cu-catalyzed cross dehydrogenative sp^3^(C-O) bond formation in salicylamides.

Formation of intramolecular C-O bond between an amide and phenolic OH via an iminium ion is challenging owing to the delocalization of *N*-lone pair electrons towards carbonyl group. By employing a reactive Cu-catalyzed system cross dehydrogenative sp^3^(C-O) intramolecular bond formation in functionalized salicylamides **(239)** was achieved to generate 2,3-dihydro-4*H*-benzo[*e*][1,3]oxazin-4-ones **(226)** (Scheme 69) [142]. Interestingly, in the case of unsymmetrically substituted amides site selective C-O bond formation preferentially occurred at electron-rich site (2^o^ C-H > 1^o^ C-H). This method featured excellent selectivity towards sterically less hindered C-H bonds (-ethyl > -isopropyl). When electronically different *N*-picolyl and *N*-benzyl amines were used, cyclization occurred towards *N*-picolyl amine, thus demonstrating electronic selectivity. *N*-phenyl amides failed to react due to the *N*-lone pair delocalization into the aryl ring leading to the instability of imine intermediate. Efficiency of the reaction was reduced by the presence of electron-withdrawing substituents (-NO_2_, 35%) due to the deactivation of nucleophile. No product was formed when -OH group was replaced by -SH. Mechanistically, initially generated Cu(II)-substrate complex **(240)** oxidized the nitrogen atom of the amide via single electron transfer and led to complex **(241)**. Subsequent formation of **(242)** through the interaction of Cu(I)/O_2_ and H-abstraction provided Cu(II)-complex **(244)**. Further intramolecular cyclization of hydroperoxo complex **(244)** offered the product **(226)**. A minor amount of ketone **(243)** was observed in a few cases through the reaction of water present in solvent with **(242)**. A gram-scale experiment was performed to show the practical application of this method. 

**Scheme 70:**
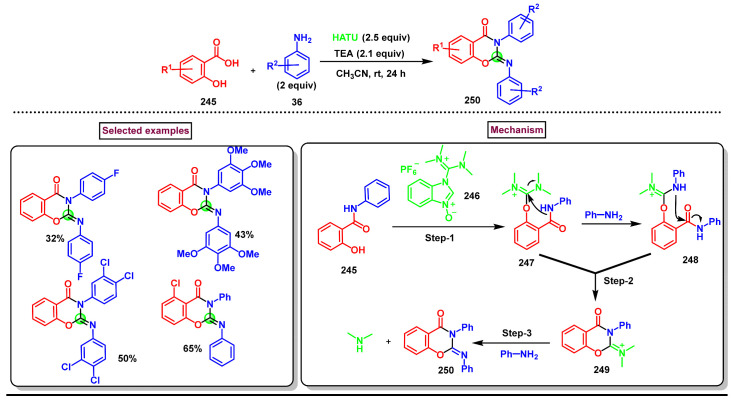
HATU-mediated carbon transfer process to access 2-imino benzo[*e*]-1,3-oxazin-4-ones.

In 2018, Dong et al. reported a HATU-promoted novel synthesis of 2-imino benzo[*e*]-1,3-oxazin-4-ones **(250)** from anilines **(36)** and salicylic acids **(245)** via HATU-mediated carbon transfer process (Scheme 70) [143]. No desired products were formed when anilines were substituted with electron-withdrawing groups such as -CN, -CF_3_. However, moderate yields (32–65%) were obtained in most of the other cases. In a few cases, DMA was used as an alternative solvent to achieve the desired product probably to overcome the solubility issues. Mechanistic studies revealed that HATU **(246)** played a crucial role in the formation of tetramethylisouronium intermediate **(247)** via carbon transfer to salicylanilide **(245)** (generated through HATU-mediated coupling of salicylic acid and aniline) which further resulted in the *N*-acyl(dimethyl)isouronium intermediate **(248)** through the reaction with an aniline precursor. Subsequent imine-iminium exchange afforded the desired products **(250)**. Though the reaction yields are low (32–65%), mild reaction conditions and readily available precursors are the appreciable merits of this report.

**Scheme 71:**
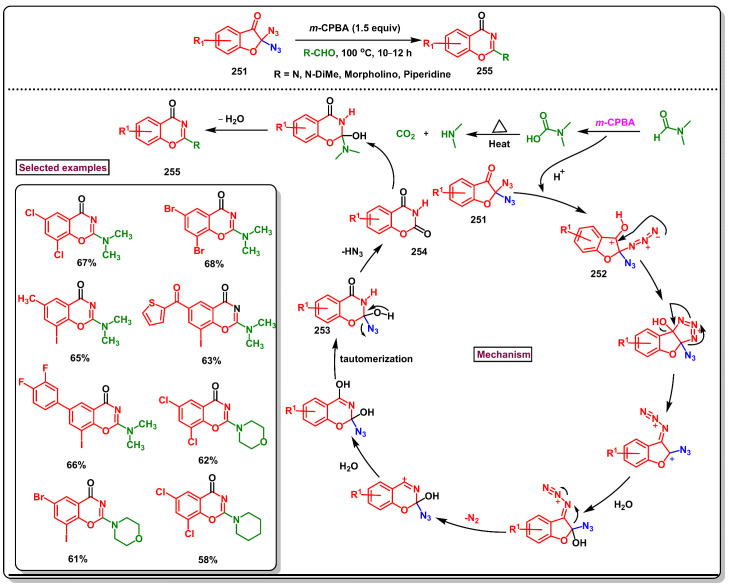
M-CPBA-promoted synthesis of 2-amino-benzo[*e*][1,3]oxazin-4-ones from 2,2-diazidobenzofuran-3(2*H*)-ones.

An interesting one-pot method to synthesize 2-amino- benzo[*e*][1,3]oxazin-4-ones from 2,2-diazidobenzofuran-3(2*H*)-ones **(255)** and corresponding amino precursors in the presence of an oxidant (*m*-CPBA) was reported [144]. This method is a complementary novel approach to typical ortho-functionalized benzoic acid-based methods. Importantly, 2-(dimethylamino)/2-(piperidin-1-yl)/2-morpholino groups can be incorporated by employing suitably formylated amino reagents (Scheme 71). When replaced with a mixture of HCHO and dimethyl amine (1:1 ratio), no desired product was obtained revealing the crucial role of formylated amino reagents. 2,2-diazidobenzofuran-3(2*H*)-ones **(251)** with broad range of substituents participated well in this transformation and the corresponding products **(255)** were obtained in moderate yields (up to 69%). Good functional group tolerance was observed with a variety of acylated, halogenated and methoxylated diazidobenzofuran-3(2*H*)-ones **(251)**. Particularly, strong electron-withdrawing -NO_2_ group also reacted smoothly with DMF to afford the product in 55% yield. Gratifyingly, *N*-formyl morpholine and *N-*formyl piperidine were also suitable for this transformation and the corresponding products **(255)** were obtained in moderate yields (54–62%). In a few cases -F atom on the aryl ring was nucleophilically displaced by amine group which might originate from hydrolyzed component of hydrazoic acid. Outlined mechanism revealed that in situ-generated *m*-chlorobenzoic acid promoted the formation of carbocation **(252)** which underwent cyclization, intramolecular Schmidt type rearrangement and nitrogen extrusion to give intermediate **(253)**. Further elimination of hydrazoic acid generated isolable **(254)** which reacted with in situ-generated dimethyl amine and eliminated water to provide the desired product **(255)**. Further synthetic transformation of 2-aminobenzoxazin-4-ones were also demonstrated by converting them into 2-hydroxyphenyl-substituted oxadiazole, triazole and triazines. 

**Scheme 72:**
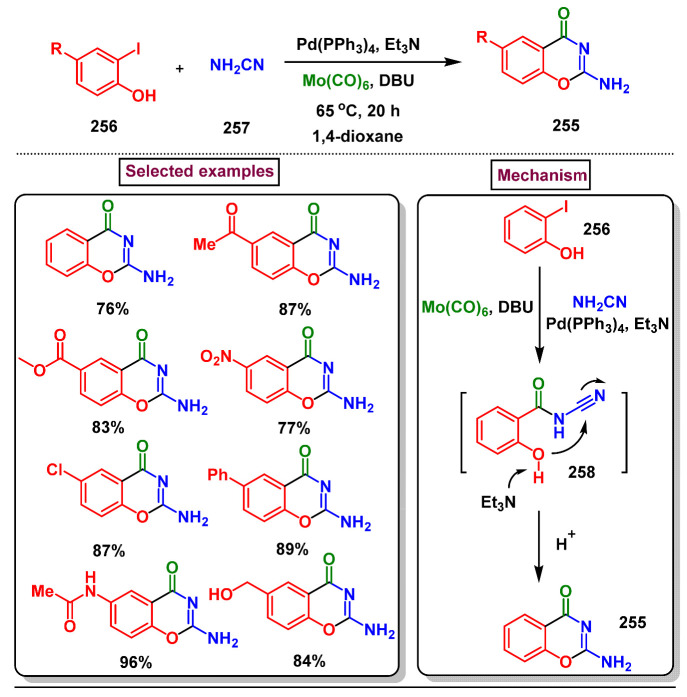
Pd-catalyzed carbonylation/cyclization approach to prepare 2-amino-4*H*-1,3-benzo[*e*]oxazin-4-ones.

Larhed and Odell et al. combinedly reported a domino, one-pot Pd-catalyzed carbonylation/cyclization strategy to prepare 2-amino-4*H*-1,3-benzo[*e*]oxazin-4-ones **(255)** from ortho-iodophenols **(256)** and cyanamides **(257)** (Scheme 72) [145]. Mo(CO)_6_ was employed as a convenient CO-releasing agent which offered a great advantage of avoiding high-pressure toxic CO gas. The intermediacy of *N*-cyanobenzamide **(258)** was confirmed by initiating a reaction of the *O*-protected precursor under the standard conditions and isolating the corresponding *O*-protected-*N*-cyanobenzamide. Excellent chemo selectivity was observed and sensitive functional groups such as ketone, ester and halogens were intact during this transformation afforded the corresponding 1,3-benzo[*e*]oxazin-4-ones **(255)** were obtained in good to excellent yields (76–96%). Strong electron-withdrawing -NO_2_ group was installed without any reduction process. It should be noted that free NH_2_ and -OH groups could also be incorporated without any appreciable side reactions. Ortho-bromophenols were found to be less reactive than ortho-iodo analogues **(256)** as the products were obtained in the range of 20–46% yields. Overall, this non-gaseous carbonylation process was proceeded with readily available precursors and stable CO source.

**Scheme 73:**
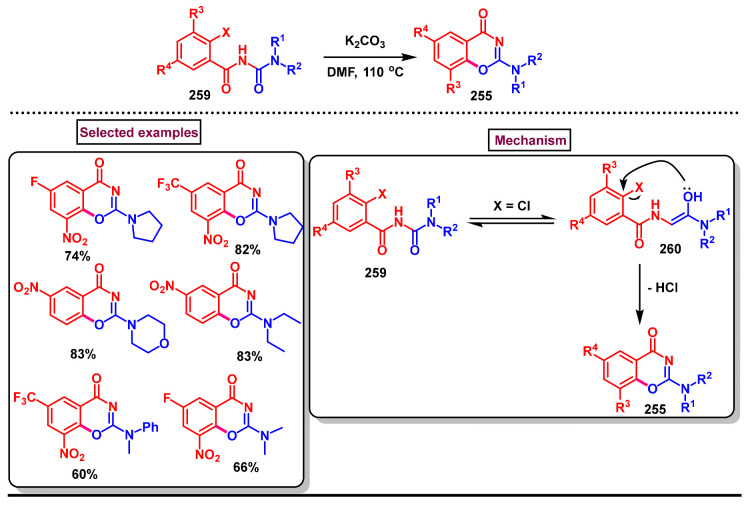
Heterocyclization of secondary amine-derived ortho halobenzoyl urea.

Heterocyclization of secondary amine-derived ortho-halobenzoyl urea **(259)** in the presence of a base delivered 2-amino-4*H*-1,3-benzo[*e*]oxazin-4-ones **(255)** under transition metal-free conditions (Zhang and Huang et al. 2015) (Scheme 73) [146]. Electron-withdrawing substituents such as F, CF_3_, NO_2_ on the aryl ring was mandatory for this transformation as unsubstituted substrates either led to trace yield or no product formation. Benzoyl ureas derived from primary amines were found ineffective under these conditions. Diverse range of amino moieties such as pyrrolidino, morpholino, *N*-methylpiperazino, dimethylamino and *N*-methylanilino groups were successfully incorporated at the C2 position. Worth mentioning, ortho-chlorobenzoyl urea exhibited higher reactivity than bromo or iodo analogues. Mechanistically, this reaction proceeded through an initial formation of benzoyl urea **(259)** then to its stable tautomer imidic acid **(260)** followed by intramolecular aromatic nucleophilic substitution.

**Scheme 74:**
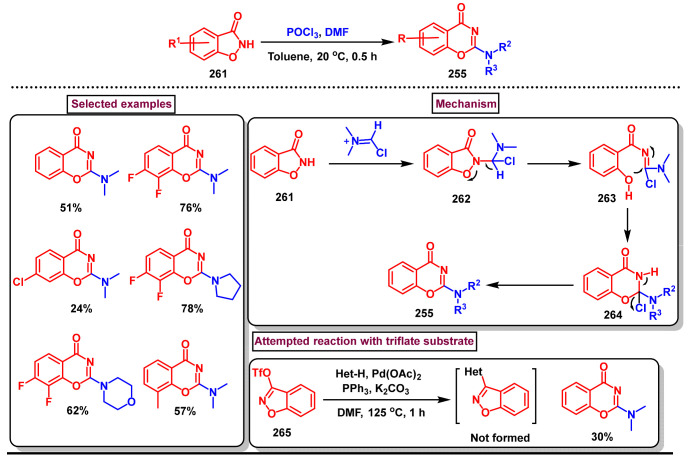
Preparation of 2-amino-4*H*-1,3-benzo[*e*]oxazin-4-ones via a ring expansion strategy.

A ring expansion strategy to access 2-amino-4*H*-1,3-benzo[*e*]oxazin-4-ones **(255)** from 1,2-benzisoxazol-3-ones **(261)** was reported by Raw et al. (2011) [147]. According to the literature reports, pinacol acetal embedded 1,2-benzisoxazol-3-ones **(261)** in the presence of Vilsmeier reagent system (POCl_3_/DMF) delivered the chlorination products, whereas in this report a ring expanded 2-amino-4*H*-1,3-benzo[*e*]oxazin-4-ones **(255)** were obtained under slightly modified reaction conditions (Scheme 74). This ring expansion strategy moderately produced the benzoxazine-4-ones **(255)** in moderate yields (24–76%) from 1,2-benzisoxazol-3-ones. No appreciable product formation observed in case of 1,2-isoxazol-3-ones, 1,2-benzisothiazol-3-one and indazol-3-ones. Other formamides such as *N*-formyl pyrrolidine, morpholine, and piperidine aldehydes resulted the corresponding amino-products in good yields (up to 76%). In the case of **(265)**, triflate group migration to DMF occurred to form Vilsmeier-type iminium ions which underwent ring expansion and resulted in **(255)**. Reaction pathway revealed that **(261)** nucleophilically reacted with in situ-generated chloro-iminium species to give **(262)**. Sequential ring opening/cyclization/dehydrochlorination offered the product **(264)**.

**Scheme 75:**
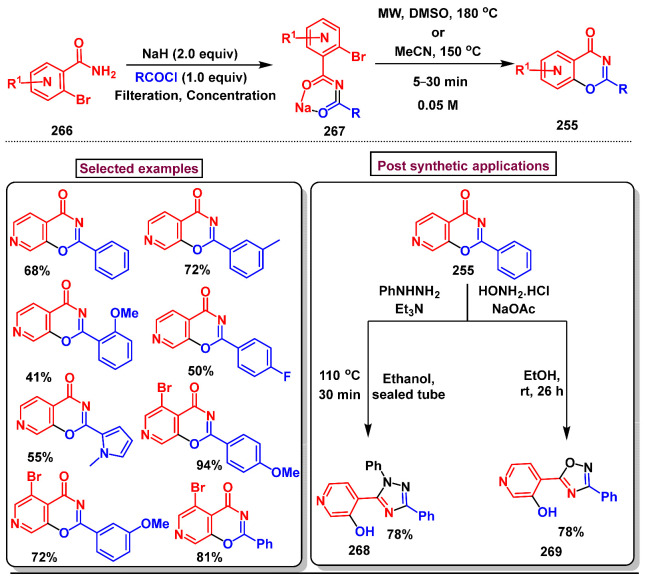
Microwave-assisted two-step synthesis of 4*H*-pyridio [1,3]-benzo[*e*]oxazine-4-ones.

An expeditive, microwave-assisted two-step synthesis of 4*H*-pyridio [1,3]-benzo[*e*]oxazine-4-ones **(255)** via an intramolecular *O*-arylation of *N*-aroyl-(iso)nicotinamides **(266)** was developed by Thorimbert et al. (Scheme 75) [148,149]. Sodium salt of *N*-aroyl-bromo(iso)nicotinamide **(267)** was isolated in the first step which simultaneously underwent intramolecular *O*-arylation and furnished desired pyrido-fused benzoxazine-4-ones **(255)** under microwave irradiation. A wide range of bromo nicotinamides and benzoyl chlorides were screened under the optimized conditions. Alkoyl chlorides were not reactive under these conditions. Similarly, 3-bromopicolinamide led to decomposition of either imide or cyclized products. Albeit lower reactivity, pyridine core involved aromatic nucleophilic substitutions and produced the benzoxazine-4-ones **(255)** in moderate to good yields (68–76%). Significant steric effects were observed for ortho-substituted aroyl chlorides (41%). In the case of 2-bromopicolinamide, due to the rapid degradation of intermediate (imide) a freshly prepared solution of intermediate was engaged in the reaction to afford the products **(255)** in good yields (63–89%). The chemical potential was further showcased by derivatization to obtain useful 1,2,4-oxadiazaole **(269)** and 1,2,4-triazole **(268)** derivatives in good yields.

## 11. Applications in N-Directed Ortho-Functionalizations Via C-H Activation

Highly selective and atom-economical, transition metal-catalyzed direct functionalization of organic molecules via C-H activation are widely recognized [150,151]. On the other hand, functionalization of unactivated C-H bond is challenging. To this end, directing groups assisted with selective C-H functionalization is a promising tool and resulted in numerous research developments [152,153]. Among the various *N-*based directing groups (pyridyl, amines, amides, oxazolines) known, benzo[*d*]oxazin-4-ones are endowed with excellent coordinating abilities that could promote regioselective C-H activation under transition metal-catalyzed conditions. In recent years, the tunable directing ability of benzoxazinones enabled various elegant methods via C-H activation and enhanced the molecular diversity and structural complexity of these ubiquitous core. Importantly, these transformations of benzo[*d*]oxazin-4-ones could occur selectively in the presence of various unactivated C-H bonds and proceed without any external coordinating species or protecting groups. Herein, we discuss a few recently developed site- selective C-H functionalization by using benzo[*d*]oxazin-4-one as a directing group.

### 11.1. Pd-Catalyzed C(sp^2^)–H Monofluorination of 2-Arylbenzo[d]oxazin-4-one

**Scheme 76:**
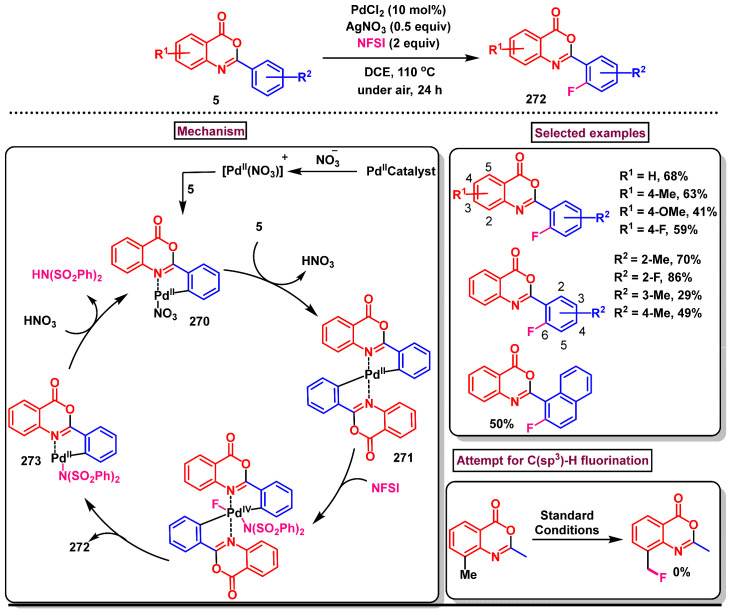
Selective mono-ortho-fluorination of 2-aryl-1,3-benzo[*d*]oxazin-4-one using NFSI.

Fluorinated compounds are known to have increased effects on the lipophilicity and biological activities of various compounds [154,155]. Generation of C-halogen bonds via Pd-catalyzed C-H activation is an effective way known in recent years [156]. However, C-F bond formation is challenging owing to the electronegativity of fluorine. By exploiting 1,3-benzo[*d*]oxazin-4-one **(5)** as a directing group, highly selective ortho-mono fluorination of 2-aryl group **(272)** was achieved under Pd-catalyzed conditions (Scheme 76) [157]. Inexpensive and non-toxic *N*-fluorobenzenesulfonimide was used as a fluorination reagent. Interestingly, C(sp^3^)-H fluorination was not detected under these conditions. Benzo ring of 2-aryl benzoxazine-4-ones **(5)** was tested with various substituents such as -Me, -OMe, -Br, -Cl, -F and the corresponding fluorinated products **(272)** were achieved in moderate yields (41–68%). 2-aryl group bearing -NO_2_, -CF_3_, halogens and ester groups worked well to afford the products in acceptable yields (up to 86%). However, heteroaryl analogues failed to yield the products. Notably, when 8-methylated benzoxazine-4-one was used no fluorination occurred at the methyl group proving the site selective nature of this method. Mechanistic proposal revealed that initially formed active cationic Pd-catalyst coordinated with **(5)** and generated the cyclo-palladium intermediates **(270)** and **(271)**. Oxidative addition of NFSI and reductive elimination of product **(272)** released the intermediate **(273)** which further reacted with HNO_3_ and regenerated the catalytic cycle.

### 11.2. Pd-Catalyzed Selective Acetoxylation, Halogenation and Hydroxylation of 2-Arylbenzo[d]oxazin-4-ones

**Scheme 77:**
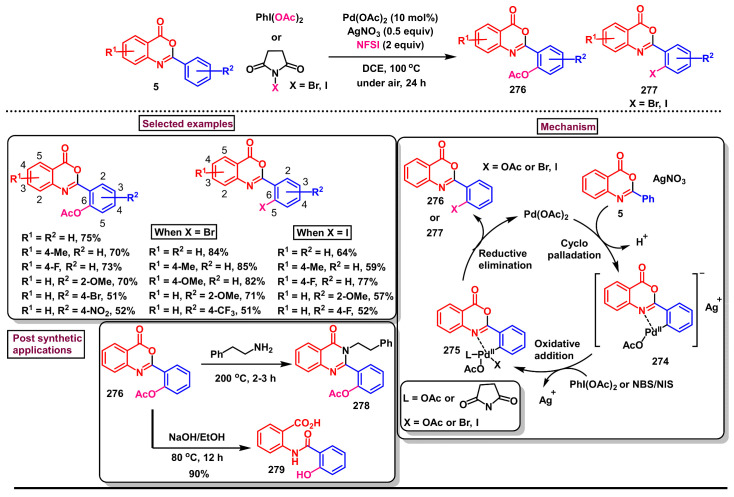
Selective mono-ortho-acetoxylation and halogenation of 2-aryl-1,3-benzo[*d*]oxazin-4-ones.

By exploiting the inherent directing ability of benzoxazinone **(5)**, a regioselective ortho-C-H actoxylation and halogenation was realized under Pd(II)/AgNO_3_ catalytic system (Scheme 77) [158]. *Ortho*-acetoxylated products **(276)** were obtained from PhI(OAc)_2_, whereas mono-halogenated products **(277)** were achieved from readily available *N*-halosuccinimides (NBS or NIS) which acted as both halogen source and oxidant. A range of unsubstituted and halogenated 2-arylbenzoxazin-4-ones **(5)** were subjected to site selective acetoxylation under the optimized conditions. It was observed that electron-donating groups (-OMe, -Me) groups led to the acetoxylated product in higher yields (70–73%) than electron-withdrawing groups (-NO_2_, -CO_2_Me) (52–58%). A gram-scale synthesis further proved synthetic efficacy. By utilizing NBS and NIS as a halogen source, diverse sets of mono-halogenated products **(277)** were obtained in moderate to good yields. As like acetoxylation, electronic nature of the substituents played a crucial role in this process. Electron donating groups favored mono halogenation. Transformation of the products into synthetically useful quinazolin-4(3*H*)-one **(278)** and ring opened derivatives **(279)** showcased the application. Initial formation of anionic cyclopalladium (II) complex **(274)** was followed by the oxidative addition of iodobenzene diacetate of *N*-halosuccinimide to generate the Pd(IV) intermediate **(275)**. Further reductive elimination afforded the product **(276)** or **(277)** and regenerated the Pd(II) catalyst.

**Scheme 78:**
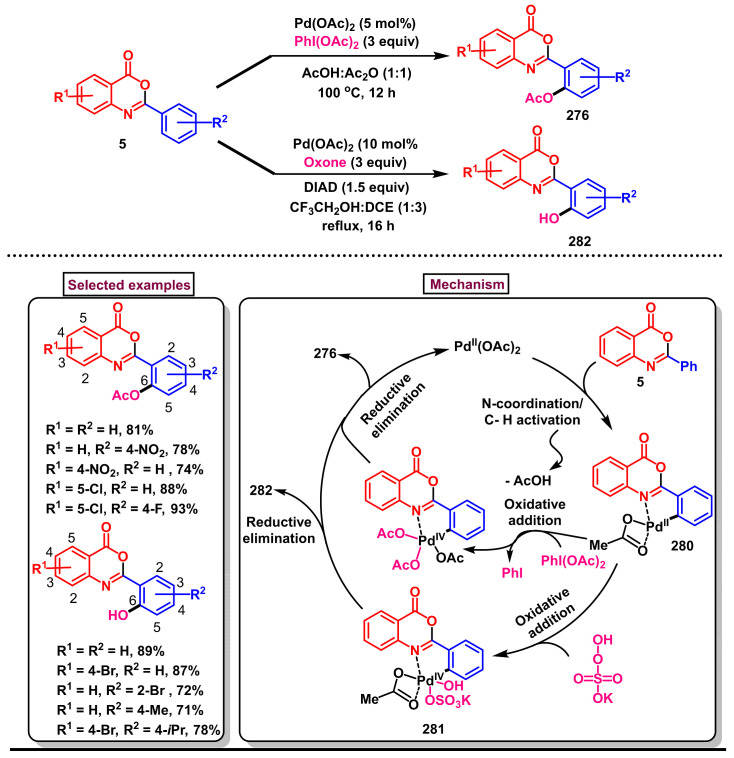
Selective mono-ortho-acetoxylation and hydroxylation of 2-aryl-1,3-benzo[*d*]oxazin-4-ones.

Another efficient strategy for the formation of carbon-hetero atom bond was realized via Pd-catalyzed direct introduction of acetoxy and hydroxy groups on benzo[*d*]oxazin-4-one derivatives **(5)** through ortho C-H activation (Scheme 78) [159]. Variously functionalized ortho-acetoxy **(276)** and hydroxy benzoxazine-4-ones **(282)** were obtained in good to moderate yields. A wide range of 2-aryl and 2-heteroaryl benzoxazine-4-ones with electronically and sterically distinct functional groups offered the mono-acetylated products **(276)** in good to excellent yields (up to 89%). By utilizing three equivalents of oxone and 1.5 equivalents of DIAD, a Pd-catalyzed ortho-hydroxylation was also achieved in good to excellent yields. It should be noted that both the processes delivered the desired products with excellent functional group tolerance. Mechanistically, coordination of Pd(II)-to the *N*-atom of benzoxazinone **(5)** and a C-H activation resulted in the Pd-complex **(280)**. Oxidative insertion of acetyl group followed by a reductive elimination furnished **(276)**. On the other hand, oxidative addition of in situ-generated hydroxy radical generated the complex which upon migratory insertion and reductive elimination offered the ortho-hydroxylated benzoxazinones **(282)**. 

**Scheme 79:**
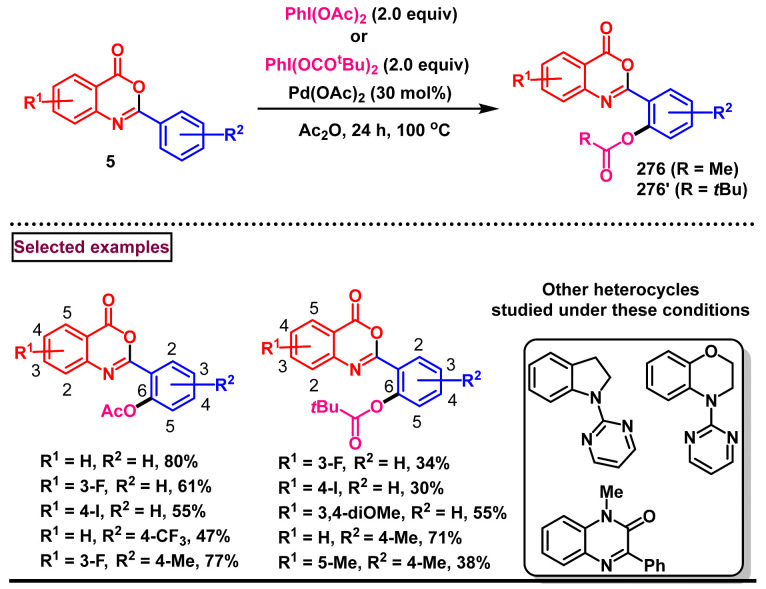
Pd-catalyzed regioselective synthesis of ortho-acyloxy benzoxazine-4-ones.

By using PhI(OAc)_2_ or PhI(OCO*^t^*Bu)_2_ as acyloxy agents, a regioselective method was developed to prepare a series of ortho-acyloxy benzoxazine-4-ones **(271** or **271′)** under Pd-catalyzed conditions (Scheme 79) [160]. As revealed by the optimizational studies, extended reaction time (30 h) resulted in the formation of diacyloxy products in significant quantities. Benzo ring of 2-aryl-4*H*-benzo[*d*][1,3]oxazin-4-ones bearing electron-donating groups resulted lower yields (44–48%), whereas higher product yields were obtained with electron-withdrawing groups (-Br, -Cl, I, -NO_2_). Reverse trend was observed for the substitutents on the 2-aryl unit of the benzoxazin-4-ones. Owing to the bulkiness, *tert*-butylacetoxylation **(271′)** was achieved in lower yields compared to the acetoxylation. Substrate scope was further successfully extended to other heterocycles such as indolines, quinoxaline-2(1*H*)-ones, and 3,4-dihydro-2*H*-benzo[*b*][1,4]oxazines. Mechanistic discussions were similar to those of Scheme 78.

### 11.3. Pd-Catalyzed Selective Benzoxylation of 2-Arylbenzo[d]oxazin-4-ones

**Scheme 80:**
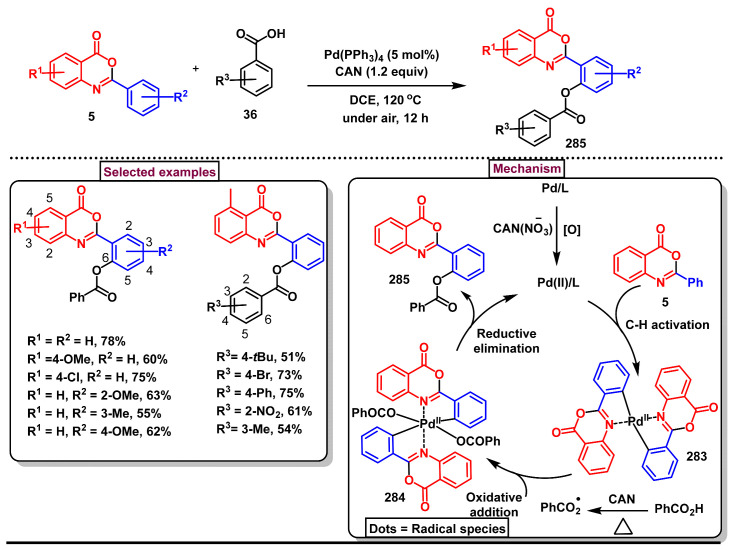
Selective mono-ortho-benzoylation of 2-aryl-1,3-benzo[*d*]oxazin-4-ones.

Direct oxygenation of C(aryl)-H bond by employing aryl carboxylic acid was reported under Pd(0)/CAN-catalyzed conditions (Scheme 80) [161]. Cerium (IV) ammonium nitrate was used as a single electron oxidant and the formation of aroyl radical was confirmed by ESI-MS analysis. This C-H benzoxylation process was investigated with a wide range of benzoic acids **(36)**. Versatility of this method was tested with various substitutions on both the aryl rings of 2-aryl-benzoxazin-4-ones **(5)**. It was observed that benzo ring with mild electron-withdrawing groups (-Br, -Cl) provided the mono-benzoxylated products **(285)** in higher yields (72–75%) compared to electron-donating groups (-Me, -OMe). In contrast, -Me, -OMe groups on the C2-aryl group delivered the products in good yields (63–71%) compared to -F, -Br, -Cl groups (38–61%). Furthermore, ortho-benzoxylation was also tested with various benzoic acid derivatives **(36)**. Irrespective of the electronic nature and substitution pattern, moderate to good yields (43–73%) were obtained in most of the cases studied. In situ*-generated* Pd(II)-species effected the C-H activation of two molecules of **(5)** to generate the complex **(283)**. Oxidatively generated aroyl radical interacted with **(283)** and subsequent reductive elimination released the product **(285)** via intermediate **(284)**. Overall, this method utilized the non-toxic, stable and easily available aryl carboxylic acid as an acylating agent and avoided sensitive acid halides or anhydrides.

### 11.4. Pd-Catalyzed Selective Olefination of 2-Arylbenzo[d]oxazin-4-ones

**Scheme 81:**
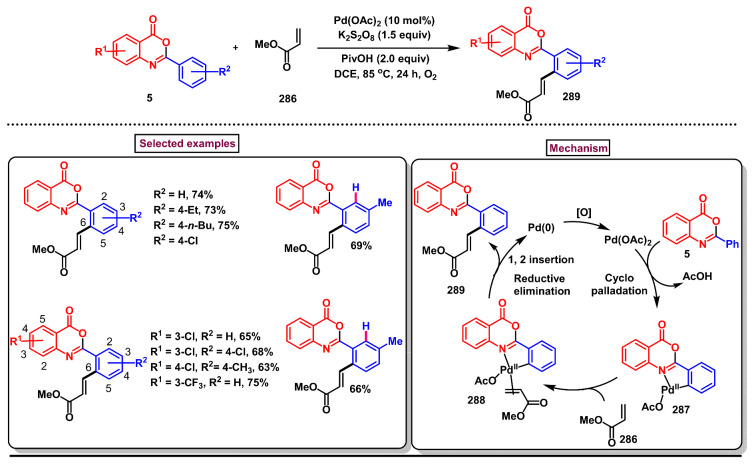
Selective mono-ortho-alkenylation of 2-aryl-1,3-benzo[*d*]oxazin-4-ones.

Direct olefination is often achieved either by Pd-catalyzed Mizorki-Heck reaction [162,163] or Fujiwara-Moritani reaction [164]. However, it either requires aryl halides or excess arene precursors. On the other hand, directing group assisted C(aryl)-H olefination is advantageous in terms of regio selectivity and practicality. To this end, olefinated-benzo[*d*]oxazin-4-ones **(289)** were prepared via Pd-catalyzed chelation assisted olefination process using activated alkenes **(286)** [165]. In the cases of ortho and meta-substituted arenes, olefination occurred at sterically less hindered position to produce single product (Scheme 81). A library of 2-aryl benzoxazin-4-ones **(5)** were subjected to alkenylation with *tert*-butyl acrylate. It was observed that electron-donating groups (-Me, -OMe, *t*Bu, *n*-Bu, Ph) and halogens (-Br, -Cl) were led to the ortho-alkenylated products **(289)** in good yields (up to 79%). Profound steric effect was observed with ortho substitutents on both the aryl rings. In case of heteroaryl bound benzoxazin-4-ones desired products were obtained in moderate yields (56–66%). Regioselective alkenylation was occurred at less hindered site in case of meta-substituted substrates. Other olefinating agents such as acrylamide, styrene, acetophenone were failed to react under these conditions. A gram-scale was demonstrated successfully in a comparable yield (61%). DFT calculations revealed that the energy of *N*-centered metallocycle was found to be 12.5 kcal/mol which is more stable compared to -O or -CO centered metallocycles. Mechanistically, the interaction of alkene **(286)** with initially formed Pd^II^cycle **(287)** generated the intermediate **(288)**. Sequential alkene insertion, *β*-H elimination and reductive elimination resulted in the product **(289)** with the oxidative regeneration of Pd(II).

### 11.5. Pd-Catalyzed Selective Amidation of 2-Arylbenzo[d]oxazin-4-ones

**Scheme 82:**
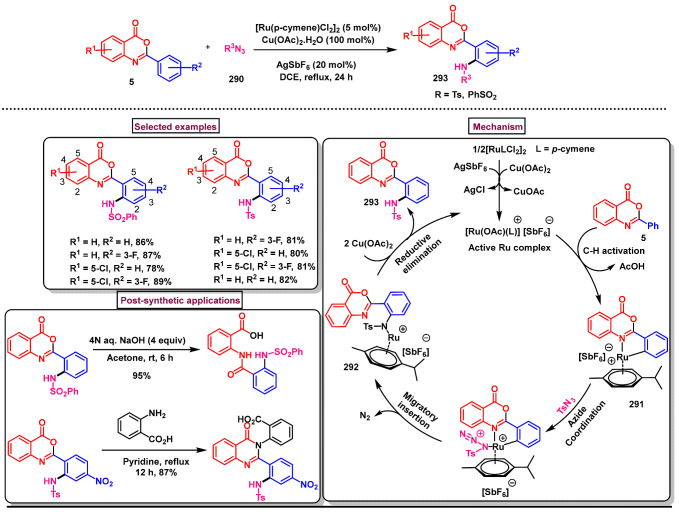
Selective mono-ortho-amidation of 2-aryl-1,3-benzo[*d*]oxazin-4-ones.

By using sulfonyl azides **(290)** as an amidating agent, a series of ortho-amido benzoxazinones **(293)** were obtained under ruthenium-catalyzed conditions via C-H activation (Scheme 82) [166]. Exclusive formation of desired mono amidated products **(293)** was supported by the chemoselective disappearance of ortho C-H which in turn was confirmed by NMR studies. Several ortho-amidated products were achieved in a chemo and site selective fashion using benzenesulfonylazides or tosyl azides. There was no remarkable difference observed between electron-donating and withdrawing groups. Especially, strongly electron-withdrawing -NO_2_, -CF_3_ groups were not found deteriorative. Various post-synthetic functionalizations such as acid/or base-mediated hydrolysis, conversion into quinazolinone were performed to showcase the synthetic potential of this method. Coordination of active Ru-species to the imine *N*-atom of **(5)** and C-H activation generated the five-member ruthenacycle **(291)**. Further coordination of tosyl azide **(290)** and migratory insertion of in situ-generated tosyl amine led to complex **(292)**. Reductive elimination of product **(293)** and reoxidation of Ru(0) completed the catalytic cycle. Overall, this direct ortho-amidation strategy proved straightforward and no undesired by-products were formed.

## 12. Biological Significance of Benzo[*d*]-Oxazin-4-Ones

Owing to the incredible structural features, benzoxazinones are attractive class of benzo fused N, O-heterocyclic scaffold which exhibit remarkable pharmacological behaviors such as anti-human coronavirus [167], anti-cathepsin G [168], inhibitor of human leucocyte elastase [169], α-chymotrypsin antagonist [170] and Protoporphyrinogen IX Oxidase [171]. Benzoxazinones when embedded with other heterocyclic skeletons such as thiazole, pyrazole, 1,2,3-triazole, 1,3,4-oxadiazole exhibit excellent anticancer activity towards various cell lines [172,173,174,175]. As an important structural congener of benzoxazinone family, benzo[*d*]oxazin-4-ones are also widely investigated for their rhomboid protease inhibition ability [176,177], intestinal epithelial anion exchanger (SLC26A3) inhibition [178], anti-inflammatory activity [179]. In this context, we focus on the anticancer activity exhibited by benzo[*d*]oxazin-4-one cores in recent years. Structural examples of potent compounds, their binding modes, in silico studies are discussed wherever applicable.

### Anticancer Activity of Benzo [1,3]oxazin-4-ones

**Figure 5:**
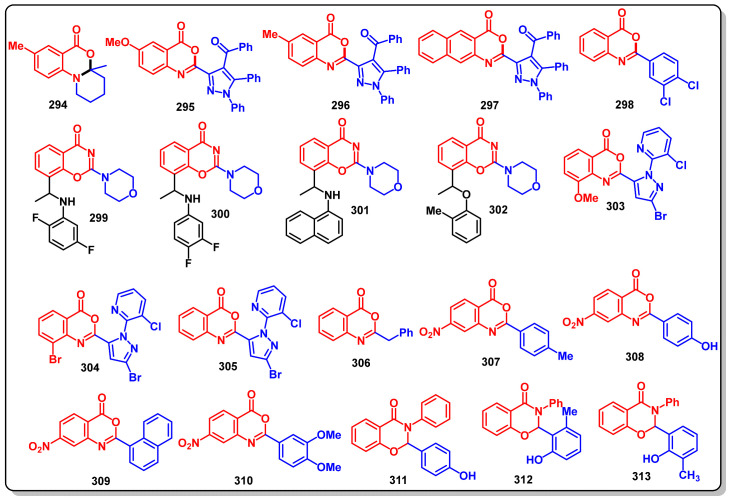
Anticancer activity of Benzo [1,3]oxazin-4-ones.

Nama and Gajula et al. [116] prepared a series of pyrrolo- and pyridobenzoxazin-4-ones and tested their in vitro anticancer potential against mouse breast cancer (4T-1), human cervical epithelioid carcinoma (HeLa) and human lung adenocarcinoma (A-549) cancer cell lines (Figure 5). In vitro cytotoxicity data revealed that compound **294** displayed distinct activities against HeLa human cervical epithelioid carcinoma cells with IC_50_ value of 25.12 μg/mL. Against 4T-1 cells, **294** exhibited strong cytotoxic potential with IC_50_ of 10.14 μg/mL. Additionally, **294** was weakly effective towards A-549 cells (IC_50_ = 80.43 μg/mL). Other potential compounds studied were from the fused pyrrolo/pyrido quinazolinone category. Tutar and Koca’s team observed that pyrazole tethered benzoxazine-4-ones effectively bound to heat shock proteins (Hsp90 and Hsp70) and inhibited their activity against selected cancer cells [180]. In vitro anticancer activity was tested against human breast cancer cells lines MCF-7 at various concentrations (12.5 μM to 100 μM) via XTT cell proliferation assay method. Molecular docking studies revealed that the best binding was obtained between compound **295** and HSP70/PDB:1S3X receptors with the binding energy of 11.20 kcal/mol and Ki value of 0.006170 μm. On the other hand, strong binding between HSP90/PDB:1YC4 receptor and compound **297** was observed with 11.20 kcal/mol binding energy and 0.039504 μm of Ki value. Preferred docking poses were stabilized by Vander Waals forces, π-anion/cation/donor-H bond and π-alkyl interactions. Further, fluorescence binding analysis showed that compound **296** bound to Hsp70 and Hsp90α with 4.91 and 4.01 μM concentration. Importantly, cell cytotoxic studies showed that compounds **295** and **296** exhibited similar potential both at 24 hours (IC_50_ = 24/21 μM) and 48 hours (IC_50_ = 19/20 μM). It should be emphasized that theoretical ADMET calculations agreed with Lipinski’ five rule. Dihalo-2-phenyl-4*H*-benzo[*d*][1,3]oxazin-4-ones were prepared and tested in vitro against breast cancer cell lines MCF-7 by MTT assay method [181]. Of all the compounds tested, 2-(3,4-dichlorophenyl)-4*H*-benzo[*d*][1,3]oxazin-4-one **(298)** showed good activity against MCF-7 (IC_50_ = 70.74 ± 3.95 μg/mL) with low cytotoxicity (50–100 μg/mL). Unsubstituted 2-phenyl benzo[*d*][1,3]oxazin-4-one displayed activity against A549 cancer cells with IC50 of 65.43 ± 2.7 μg/mL. In silico molecular docking studies towards methionyl-tRNA synthetase (MRS) showed that docking score −74.74 ± 0.05 kcal/mol was observed for 2-(3,4-dichlorophenyl)-4*H*-benzo[*d*][1,3]oxazin-4-one **(298)**. All the halogenated compounds had steric interaction with the receptor due to which high docking scores were observed. A novel isostere class of benzoxazine-4-ones which are structurally analogues to TGX-221 were prepared in which 4*H*-benzo[*e*][1,3]oxazin-4-one was placed instead of 4*H*-pyrido[1,2-*a*] pyrimidin-4-one [182]. These compounds were tested for their efficiency in inhibiting PI3Kβ enzymes. To resemble the structural features of TGX-221, these compounds were installed -CH(CH_3_)-NH-type and -CH(CH_3_)-O-type linkers. 2,4- and 3,4-difluorinated compounds **(299)** and **(300)** displayed efficient inhibition with IC_50_ of 70 nM and 63 nM. Among the compounds tested, napththyl analogue **301** was found to be efficient (IC_50_ = 49 nM) which is better than TGX-221 (IC_50_ = 65 nM). Para-substituted compounds in this series exhibited poor PI3Kβ inhibition. On the other hand, aryloxy ethyl analogues exerted low reactivity towards PI3Kβ inhibition. Compounds **302** showed comparable reactivity and found better among the compounds studied (Figure 5). Four 1,3-benzoxazin-4-one derivatives were prepared and evaluated against H7402, HeLa, SGC7901, SK-RC-42, and A549 cancer cell lines [7]. By inhibiting the overexpression of c-Myc via the interaction with G-quadruplex, the expected anticancer effects were achieved. Cytotoxicity assay of normal cell (L02) and all these five cell lines were performed at a concentration of 2.5 to 30 μM. Cell viability was reduced to 20.4% at 10 μM drug concentration when **303** was exposed to H7402 cells (IC_50_ = 12.5 ± 0.8 μM). Compound **304** led to rapid reduction in cell viability of HeLa cell lines at 15 μM for 24 h. In addition, **304** showed potential activity against SGC7901 by reducing the cell viability to 60.2% at 30 μM for 12 h (IC_50_ = 9.55 ± 0.37 μM). Notably, all the four compounds displayed strong inhibition against A549 cells with IC50 values ranging from 3.80 ± 0.12 μM to 7.35 ± 0.27 μM. Compared to the normal L02 human cell lines, compounds **303**, **304**, **305** shown several fold higher inhibitions towards all the five tumor cell lines (3.18-73.9 folds). RT-PCR based investigation of c-Myc mRNA expression on the cancer cells was carried out in the absence and presence of compounds. It was revealed that the observed inhibitory effects were due to the downregulation of c-Myc gene expression by the examined compounds either indirectly or directly through the interaction of benzoxazinone derivative with G-quadruplex. 

**Figure 6:**
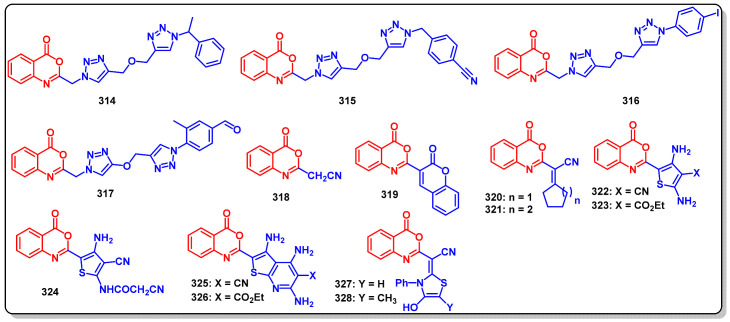
Anticancer Activity of Benzo [1,3]oxazin-4-ones.

Radwan et al. tested a series of benzoxazinones for their anticancer potential against human HepG2 cancer cell lines using SRB assay and MTT assay methods [183]. Under SRB assay method, benzoxazinone **(306)** displayed higher activity with cell viability of 76.24%. Superior activity of benzoxazinone was related to the electronegativity of the -O and the H-bond accepting nature of carbonyl group. Mechanistically, benzoxazinone **(306)** could react nucleophilically with the hydroxyl group of the enzyme and lead to the ring opened/acylated enzyme which is an inactive form. The activity of benzoxazinone was tested at various concentrations such as 0.01, 0.1,1.0, 10, 100 μg/mL against HepG2 cancer cells. Various other heterocycles were also prepared and tested against cancer cell lines. It was observed that quinazolinone-hydrazide was potential against human epithelioid carcinoma cancer cells (Hela). The same research group demonstrated the anticancer effects of 2-benzyl-3,1-benzoxazin-4-one against human lung cancer cells (A549) with IC_50_ value of 53.9 μM [184]. Shahzad’s team prepared a series of benzoxazinone substrates and screened their in vitro cytotoxic activity effects on the HeLa cancer cell lines with reference to the standard Doxorubicin drug [15]. Poential compounds of this series were found to be **307**, **308**, **309** and **310**. Upon interaction with selected benzoxazinones, cell viability of Hela cells were in the range of 28.53% to 87.22%. However, the standard drug displayed 19.98% of cytotoxic effect. To understand the mode of binding and active sites molecuar docking studies were performed. Results showed that various interactions between the target compounds and the capase protein receptors occurred. Especially, the amino acid residue Leucine was involved in the binding majorly. In silico studies confirmed that H-bond between electronegative atom of benzoxazinone and the Leuicine residue, π-π, and π-alkyl interactions were found predominantly. Compounds **309** and **307** exerted similar binding affinities (−7.4 and −7.3) which are comparable to standard drug doxorunicin (−7.4). On the other hand, experimentl data described that **308** and **310** bearing -OH and -3, 4-dimethoxy groups exhibited higher activity. Overall, activating groups such as -Me, -OMe, -OH at the para-positions demonstrated superior activity. Kesuma and coworkers studied the anticancer activity of 2-Phenyl-4*H*-benzo[*d*][1,3]oxazin-4-one **(5)** towards human lung cancer cell lines (A549) via MTT assay method with reference to standard drug Doxorubicin [185]. In silico docking studies revealed that the benzoxazinone was bound to the active sites of methionyl-tRNA synthetase receptor. Compound **5** exhibited activity against A549 with IC_50_ value of 65.43 ± 2.7 μg/mL (IC_50_ = 14.61 ± 2.3 μg/mL for Doxorubicin) Analysis of interaction of **5** with MRS receptor only displayed steric interaction with amino acid residues (Gly 23; His 24, His 323, Gly 324; Val 326) and the obtained binding score was -76.04 ± 0.03 Kcal/mol. Kandale et al. reported a series of 2-(2,3-disubstituted phenyl)-1,3-benzo[e]oxazin-4-ones and evaluated their in vitro anticancer activity against MCF-7 cell lines via MTT assay method [186]. The percentage inhibition of benzoxazine-4-one derivatives at various time intervals showed that **311**, **312** and **313** showed stronger activities with the IC_50_ values in the range of 0.89 µg, 1.02 µg and 1.19 µg. After 48 hours incubation, **311** displayed 98.18% inhibition at a dose of 100 µg. Kishore et al. prepared a new series of 1,2,3-tetrazole fused benzo[*d*][1,3]oxazinones and studied for their potential in vitro anticancer effectiveness against human colorectal carcinoma (HCT-116) and human lung cancer cells (A549) via MTT assay method [187]. The assessment was performed at various concentrations such as 2, 6.75, 12.5, 25, 50 µM with reference to the standard drug doxorubicin. Compounds **314** and **315** displayed maximum activity with the IC_50_ values of 2.45 ± 0.14 µM and 3.02 ± 1.04 µM for both the cell lines studied (Figure 6). In comparison to the standard drug (IC_50_ = 2.45 ± 0.14 µM), **316** and **315** showed up a significant cytotoxicity towards human colorectal carcinoma cancer cells (HCT-116) with IC_50_ of 6.92 ± 0.26 µM and 19.4 ± 0.22 µM. Mode of binding and binding patterns are studied by in silico molecular docking experiments. Compounds **304** and **305** were perfectly bound the receptor protein pocket with the docking score of −7.99 kcal/mol and −7.95 kcal/mol, whereas **317** had a score of −6.71 kcal/mol. Hydrogen bonding interactions and various hydrophobic interactions (π-alkyl, π-π, π-σ) and Van der Waals interactions were observed between the amino acid residues in the protein pocket and the potential compounds. Identified potential compounds were investigated for their physiochemical and ADMET properties. Mohareb’s team prepared a series of benzo[*d*][1,3]oxazin-4-ones embedded with coumarin, thiophene, ylidene, thiazole, thieno[2,3-*b*]pyridine and pyrazole derivatives [188]. These novel compounds were tested for their in vitro anticancer activity against human cancer cells such as HEPG2, human breast cancer (MCF) and nasopharyngeal carcinoma (HONE1) along with normal fibroblast cell lines (WI38). Investigations revealed that benzo[*d*][1,3]oxazin-4-one **318** displayed maximum inhibition against HEPG2 with the IC_50_ value of 365 nM where as low inhibition was found towards HONE1 and MCF-7. 3-chromenyl derivative **319** exhibited strong inhibitory effects against all three cancer cell lines with IC_50_ value ranging from 168 to 410 nms. Notably, ylidene analogues **320** exerted high inhibition for HONE1 cancer cells (IC_50_ = 220 nM), whereas **321** showed good effect towards HEPG2 (IC_50_ = 399 nM). In case of thiophene based derivatives, **322**, **323** and **324** were shown promising effects against all three cells (IC_50_ = 128 to 766 nM). Thieno[2,3-*b*]pyridines **325** and **326** were found effective against MCF cell lines (IC_50_ = 264 to 212 nM) (Figure 6). On the other hand, thiazole **328** showed high inhibition for MCF-7 (IC_50_ = 116 nM), whereas **327** was effective towards HONE1 cells (IC_50_ = 262 nM). In silico docking studies revealed that the selected compounds were docked into the active ATP binding sites of EGFR kinases. Hydrogen bonding and aromatic interactions were involved during the binding process.

## 13. Conclusions

Benzoxazin-4-ones are of high-value N, O-heterocyclic compounds with promising therapeutic properties as well as crucial in polymers, optoelectronics, fluorescent materials, cosmetics and food industries. Owing to their structural features, they are effectively utilized as a versatile synthon in several chemical transformations. Numerous synthetic methodologies were reported in recent years Via transition-metal-catalyzed carbonylation, dipolar cycloaddition, cascade annulations, cross-dehydrogenative coupling, isocyanide insertion reactions and more. In this account, we have systematically reviewed the synthetic advancements towards the benzoxazine-4-ones from substrates such as anthranilic acids, amides, isatoic anhydrides, isatins, indoles, aryl halides and benzoic acids under various conditions. Detailed schematic representations, selected examples, mechanistic description, and post-synthetic applications are discussed wherever applicable. Synthetic strategies developed from 2010 onwards to access benzo[*d*][1,3]oxazin-4-ones, benzo[*e*][1,3]oxazin-4-ones, 1,2-dihydo-benzo[*d*][1,3]oxazin-4-ones, and fused benzo[*d*][1,3]oxazin-4-ones are given specific importance. Due to the inspiring structural features this scaffold has been exploited in various directing group assisted ortho functionalizations via C-H activations. In the later part of this review, a series of site-selective ortho functionalizations of 2-arylbenzoxazin-4-ones are discussed. In future perspective, due to the amplified utility of benzoxazine-4-one derivatives in drug chemistry, material and organometallic applications, these skeletons are crucial choice of N, O-heterocyclic templates. Additionally, to showcase the biological importance of these ubiquitous cores, we have also emphasized the recent studies on anticancer evaluations. Synthetic methods presented in this review demonstrate that remarkable efforts have been made to develop unique routes to access the benzoxazinone cores. Though heteroannulations or cross-dehydrogenative coupling strategies are still a selective and prominent methods to produce benzoxazinone skeletons, they often demand pre-functionalized substrates that require lengthy preparatory process and expensive transition metal catalysts (Au, Rh, etc.). On the other hand, oxidative cascade annulations from commercially available, bench stable precursors, reusable ionic liquid-mediated cyclization are more general, promising and appealing green alternatives. Advantages of these synthetic methods are that they do not require pre-functionalized reactants and proceed under mild conditions with higher yields. These merits enable the preparation of benzoxazinone scaffolds with broad structural diversity and scalability. Further refinement to these eco-friendly methods would also facilitate the researchers towards the convenient access of these ubiquitous cores and study the inherent challenges in N-directed ortho functionalization of benzoxazinones. Overall, we hope that the innovative approaches, applications, and biological information discussed in this report will serve as a reference source and enable the researchers to contribute to the synthesis of related N, O -heterocycles and their biological investigations.

## Data Availability

No data were used for the research described in the article.
